# Synthesis and Photophysical Properties of β-Alkenyl-Substituted BODIPY Dyes by Indium(III)-Catalyzed Intermolecular Alkyne Hydroarylation

**DOI:** 10.1021/acs.joc.3c02951

**Published:** 2024-03-19

**Authors:** Ana Da Lama, José Pérez Sestelo, Luis A. Sarandeses, M. Montserrat Martínez

**Affiliations:** CICA—Centro Interdisciplinar de Química e Bioloxía and Departamento de Química, Universidade da Coruña, 15071 A Coruña, Spain

## Abstract

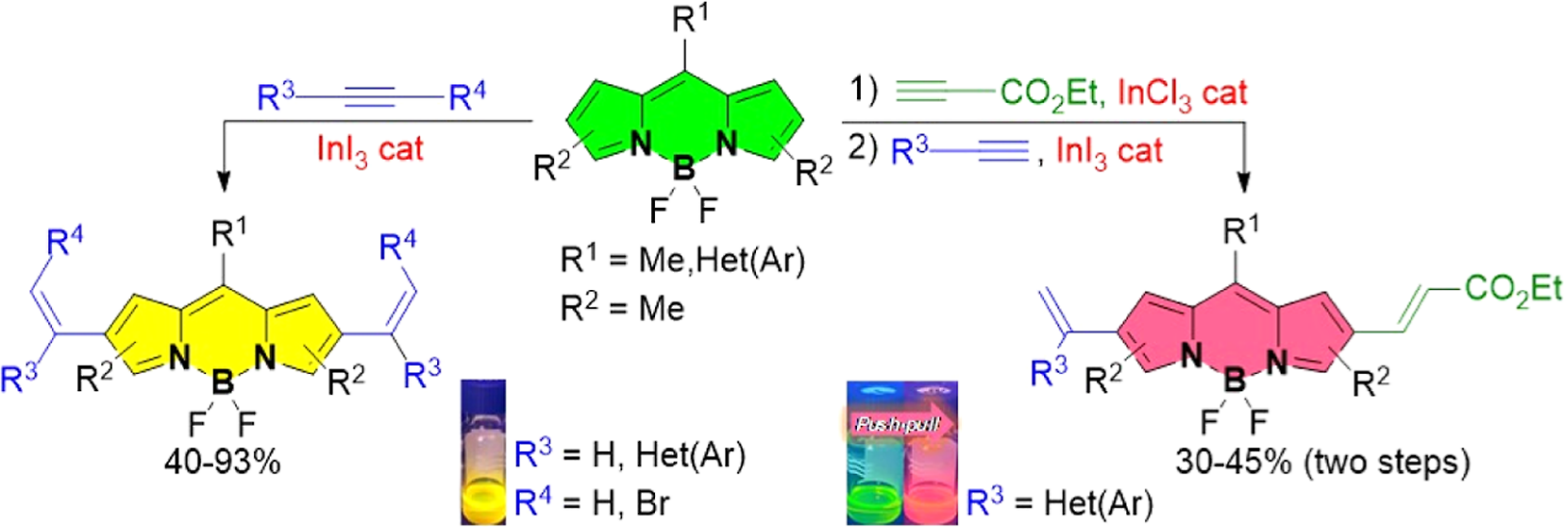

A new atom-economical synthesis of β-alkenyl-substituted BODIPYs via indium(III)-catalyzed intermolecular alkyne hydroarylation with *meso*-substituted BODIPYs is described. While catalysis with InI_3_ allows the double β-functionalization of BODIPY, resulting in regioselectively branched β,β′-disubstituted alkenyl BODIPYs, catalytic InCl_3_ enables the formation of linear β-substituted alkenyl BODIPYs. Subsequent In(III)-catalyzed intermolecular alkyne hydroarylation allows the synthesis of unsymmetrical *push*–*pull* BODIPY derivatives. Therefore, indium catalysis offers complementary regioselectivity in good chemical yields and functional group tolerance. The resulting BODIPY dyes displayed bathochromically shifted absorption and emission according to the electron-nature of the substituents in the alkenyl moiety with high molar extinction coefficients (ε up to 88,200 M^–1^ cm^–1^) and quantum yields (0.14–0.96).

## Introduction

4,4-Difluoro-4-bora-3a,4a-diaza-*s*-indacene (BODIPY) and derivatives are a class of valuable small-molecule fluorophores of interest in diverse research areas like bioimaging,^1^ fluorescent probes,^2^ photodynamic therapy,^3^ photocatalysis,^4^ optoelectronic devices,^5^ and so forth.^6^ The growing success of these rigid π-conjugated complexes lies to their excellent features such as narrow absorption and emission bands, high fluorescent quantum yield, good photostability, and solubility.^7^ Hitherto, a plethora of efforts have been made to fine-tuning their properties by introduction of suitable substituents at the different positions of the BODIPY scaffold and in particular, conjugated substituents at C2 and C6 (β) positions through alkenyl extension usually led to red-shifted emission bands with application as fluorescence probes to biothiols,^8^ DNA,^9^ or as photosensitizer.^10^

**Scheme 1:**
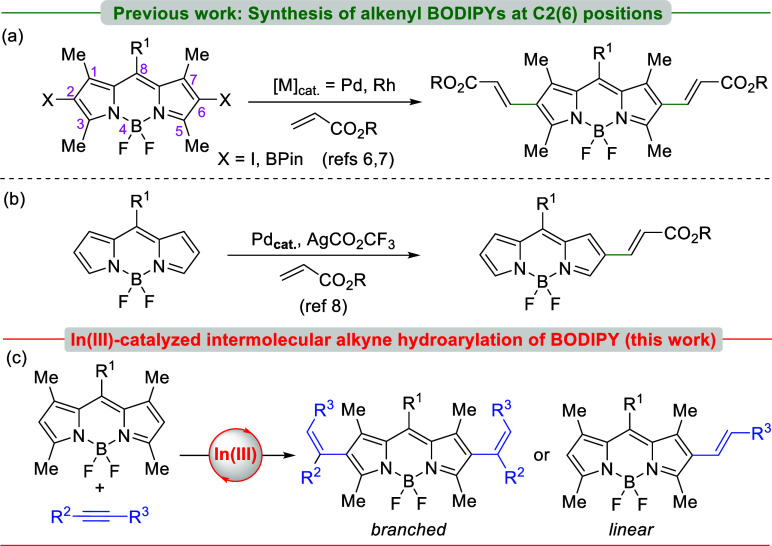
Synthetic Approaches to Obtain β-Alkenyl BODIPYs

Among the different synthetic approaches so far employed to obtain β-alkenyl BODIPYs, the metal-catalyzed cross-coupling reactions from halogenated or borylated derivatives represent a straightforward methodology (Scheme 1a).^11,12^ Late-stage functionalization of the BODIPY core has also been exploited via C–H functionalization using palladium-mediated oxidative olefination (Scheme 1b)^13^ To the best of our knowledge, the synthesis of branched and linear β-alkenyl BODIPYs through intermolecular metal-catalyzed alkyne hydroarylation with BODIPY is unknown, despites its great potential in terms of economy. By taking advantage of the reactivity of C2(6) positions of the BODIPY framework,^7^ we envisioned the synthesis of alkenyl-substituted BODIPYs through intermolecular In(III)-catalyzed alkyne hydroarylation (Scheme 1c).

Since Shirakawa and Kawakami reported the first examples of Friedel–Crafts alkenylation of arenes using alkynes with indium(III) triflate as the catalyst,^14^ this transformation has received much attention, because this approach offers an attractive alternative to Heck and cross-coupling reactions, as can be catalyzed by different transition metals such as palladium, platinum, gold, and iron,^15^ that was later expanded to aromatic heterocycles.^16^ Indium(III) has also proven to be an efficient π-Lewis acid in inter- and intramolecular hydroarylation transformations which offers advantages in terms of cost and low toxicity.^17^ In this research area, we have developed In(III)-catalyzed intramolecular hydroarylation reaction of arenes and hydroalkoxylation and hydroamination reactions,^17a^ including tandem cycloisomerization reactions.^18^ Herein, we report the indium(III)-catalyzed intermolecular alkyne hydroarylation with BODIPY dyes to access to β-alkenyl BODIPYs and the study of their photophysical properties (Scheme 1c).

## Results and Discussion

At the outset of this investigation, a variety of *meso*-substituted BODIPYs **1a–d** were synthesized through our recently reported efficient microwave-assisted one-pot pathway.^19^ Initially, based on our previous experience, commercially available InI_3_ was chosen as the catalyst. Treatment of BODIPY **1a** with phenylacetylene (molar ratio of 1:5) in the presence of InI_3_ (10 mol %) in DCE (∼0.1 M) at 80 °C afforded branched β-alkenyl BODIPY **2** in 11% yield, as a result of intermolecular hydroarylation with Markovnikov regioselectivity, along with a large amount of remaining unreacted **1a** (entry 1, Table 1). No other regioisomer was detected in the reaction mixture. Likewise, when the molar ratio was increased from 1:5 to 1:10 and to 1:14, compound **2** was obtained in 21% and 38% yields, respectively, accompanied by the formation of 2,6-dialkenyl BODIPY **2a** (entries 2–3, respectively). Other solvents such as toluene and CH_3_CN were less effective (entries 4–5). Moreover, the use of 10 mol % of Au(III), Au(I), or Pt(II) salts was ineffective catalysts recovering in all cases the starting compound **1a** (entries 6–8). However, using 20 mol % of InI_3_, **2a** was isolated in 90% yield after 8 h (entry 9). It was found that both InBr_3_ and InCl_3_ catalysts allowed a mixture of **2** and **2a** in moderate yields (entries 10–11, respectively). On the other hand, the use of In(OTf)_3_ (20 mol %) in DCE at 80 °C or toluene at 100 °C did not afford the target product **2a** (entries 12–13).

**Table 1:**
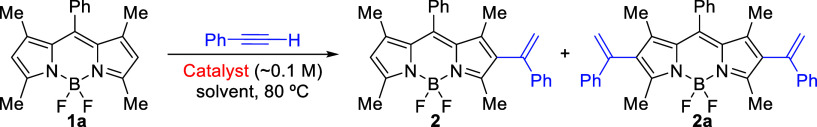
Intermolecular In(III)-Catalyzed Double Hydroarylation with Phenylacetylene with BODIPY **1a**

entry	catalyst (mol %)	solvent^a^	time (h)	yield (%)^b^	
				**2**	**2a**
**1**^c^	InI_3_ (10)	DCE	48	11	-
**2**^d^	InI_3_ (10)	DCE	24	21	4
**3**	InI_3_ (10)	DCE	24	38	14
**4**	InI_3_ (10)	toluene	24	25	1
**5**	InI_3_ (10)	CH_3_CN	24	nr	nr
**6**	AuCl_3_ (10)	DCE	48	nr	nr
**7**	PtCl_2_ (10)	DCE	48	nr	nr
**8**^e^	Au(PPh_3_)Cl (10)	DCE	48	nr	nr
**9**	InI_3_ (20)	DCE	8 h	-	90^f^
**10**	InBr_3_ (20)	DCE	48	36	29
**11**	InCl_3_ (20)	DCE	48	39	10
**12**	In(OTf)_3_ (20)	DCE	72	12	-
**13**^g^	In(OTf)_3_ (20)	toluene	48	nr	nr

a Reactions were carried out using **1a** (1 mmol, ∼ 0.1 M) and 14 equiv of phenylacetylene.

b Yields estimated by ^1^H NMR using CH_2_Br_2_ as an internal standard.

c Reaction using 5 equiv of phenylacetylene.

d Reaction using 10 equiv of phenylacetylene.

e AgSbF_6_ (10 mol %) was also used.

f Isolated yield.

g Reaction carried out at 100 °C.

**Scheme 2:**
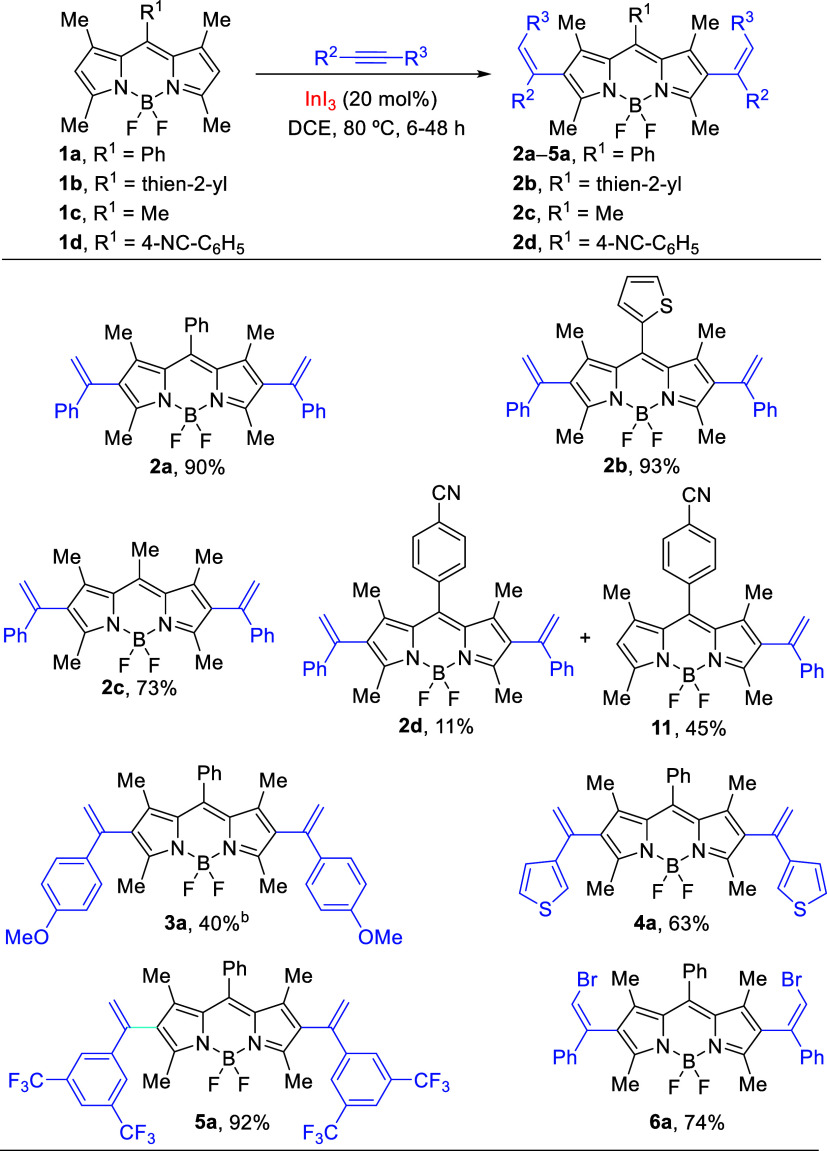
Synthesis of Branched 2,6-Dialkenyl BODIPY Dyes through π-acid Catalysis Reactions were carried out using **1a–1d** (1 mmol, ∼ 0.1 M) and 14 equiv of arylacetylene. Monohydroarylated product was also obtained in 32% yield.

After the optimal reaction conditions were determined, we examined the scope of the reaction by using *meso*-substituted BODIPYs **1b–d** (Scheme 2). The reaction of **1b**, with a thien-2-yl group at the *meso* position, using 20 mol % of InI_3_ in DCE at 80 °C gave the double hydroarylation product **2b** in 93% yield. In a similar way, *meso*-methyl BODIPY **1c** also produced the double hydroarylated product **2c** in 73% yield. The role of electronic effects was also explored using BODIPY **1d** bearing a 4-cyanophenyl substituent at the *meso* position. Under the previous reaction conditions, the reaction of **1d** with phenylacetylene provided only 2,6-dialkenyl BODIPY **2d** in 11% along with the monosubstituted BODIPY **11** in 45% yield. This result could be attributed to the basic nitrogen of cyano group or to the low reactivity of BODIPY, resulting in the electron-withdrawing effect of the cyano group.

To further extend the scope of this transformation, the electronic effect of the substituents with respect to the alkyne component was explored. In this endeavor, the reaction of **1a** with 4-methoxyphenylacetylene provided **3a** in moderate yield (40%) accompanied by monosubstituted BODIPY (32% yield). In addition, treatment of **1a** with 3-ethynylthiophene afforded **4a** in 63% yield. The indium(III)-catalyzed procedure with BODIPY **1a** also proved efficient using 1-ethenyl-3,5-bis(trifluoromethyl)benzene as the alkyne partner, arising selectively the dihydroarylated product **5a** in 92% yield. Interestingly, the polyfluorinated fluorophores based on the BODIPY framework are of particular interest for the preparation of fluorescent fluorocarbon nanoemulsions, which can be used for simultaneous fluorescent and ^19^F MRI imaging.^20^

During our research, we also examined (bromoethynyl)benzene^21^ as an alkyne partner in the InI_3_-catalyzed reaction with BODIPY **1a**. The participation of haloalkynes in the metal-catalyzed nucleophilic addition^22^ to generate alkenyl-halides constitutes a transformation with significant synthetic value.^23^ Alkenyl halides are also a pivotal class of compounds for chemical transformations in natural product total synthesis^24^ and also have found applications in photodynamic therapy when are linked to a BODIPY scaffold.^25^ In the event, the reaction of **1a** with (bromoethynyl)benzene under the stablished reaction conditions (20 mol % of InI_3_, DCE, 80 °C) gave selectively (*Z*)-2,6-dialkenyl BODIPY **6a** in 74% yield (Scheme 2). The Markovnikov regioselectivity was determined in **6a** by the NOE experiment (see the Supporting Information).

Remarkably, indium(III) demonstrated a good ability to promote intermolecular alkyne hydroarylation with *meso*-substituted BODIPY dyes, yielding branched 2,6-alkenyl BODIPYs with Markovnikov regioselectivity independent of the electronic nature of the alkyne and BODIPY moieties.

**Scheme 3:**
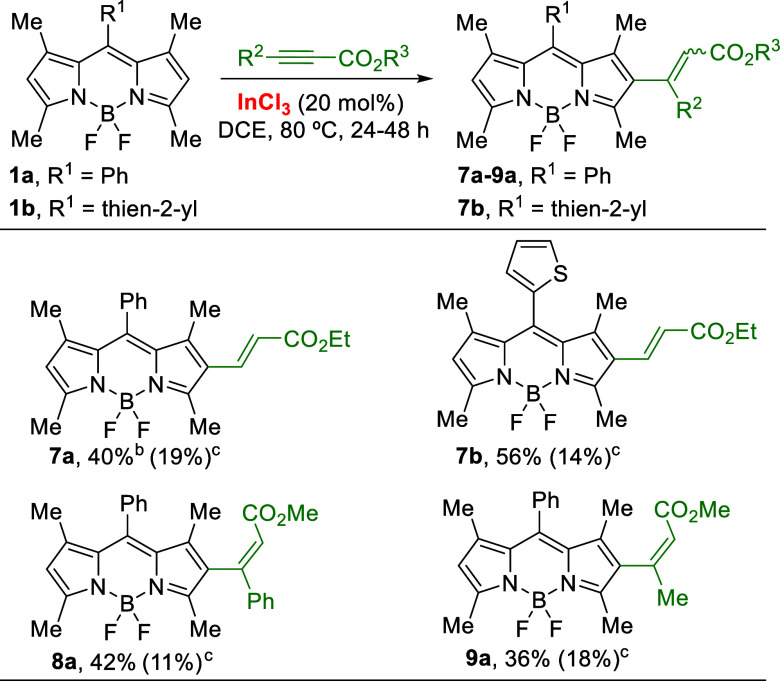
Synthesis of Linear 2-Alkenyl BODIPY Dyes through σ-Catalysis Reactions were carried out using **1a–1b** (1 mmol, ∼ 0.1 M) and 14 equiv of alkyne. InI_3_ (20 mol %) was used. In parentheses, yield of the 2,6-dialkenyl products.

As the next step, we studied the hydroarylation reaction of BODIPY with electron deficient alkynes under indium(III) catalysis. In this endeavor, we found that **1a** reacts regioselectively with ethyl propiolate under the optimal reaction conditions (20 mol % of InI_3_, DCE, 80 °C), affording the linear β-addition product **7a** in 20% yield (Scheme 3). The stereochemistry of (*E*)-2-alkenyl BODIPY **7a** was assigned by ^1^H NMR spectroscopy on the basis of the coupling constants of alkenyl hydrogens (*J* = 16.2 Hz, see the Supporting Information). Interestingly, we found that using InCl_3_ (20 mol %) as the catalyst in DCE at 80 °C, the yield increased up to 40%. This result could be attributed to the oxophilic σ-Lewis acid character of the InCl_3_,^26^ and therefore, we hypothesized that the coordination mode of ethyl propiolate to InCl_3_ implies a σ-catalysis instead of π-catalysis. Furthermore, the reaction of **1b** with ethyl propiolate afforded the expected β-addition, yielding (*E*)-2-alkenyl BODIPY **7b** (56% yield). In addition, the reactions of **1a** with 3-substituted propiolates, under the same reaction conditions, provided the trisubstituted alkenyl BODIPYs **8a** and **9a** in 42% and 36% yields, respectively, with (*Z*)-stereochemistry (Scheme 3). The assignment of stereochemistry of both compounds was confirmed by NOESY spectra (see the Supporting Information). Interestingly, the products obtained containing ester moieties are synthetically useful and might provide opportunities to development of BODIPY conjugates.^27^

These experimental findings revealed that the branched 2,6-dialkenyl BODIPY dyes can be obtained regioselectively as a result of an initial activation of alkyne by π-coordination to InI_3_, followed by the *anti*-addition of the BODIPY through the β-position, further aromatization and protodemetalation, although a mechanism involving an alkenyl cation intermediate could not be discarded.^17b,17d^ On the other hand, linear (*E*)-β-alkenyl BODIPYs could be formed through an initial σ-coordination of InCl_3_ with the carbonyl moiety followed by the β-conjugated addition of the BODIPY to form a zwitterionic allenyl enolate. The formation of (*Z*)-trisubstituted alkenes in the reaction of substituted propiolates can be attributed to the high Lewis acidity of InCl_3_, which would induce an alkene isomerization equilibrium.^17d^

**Scheme 4:**
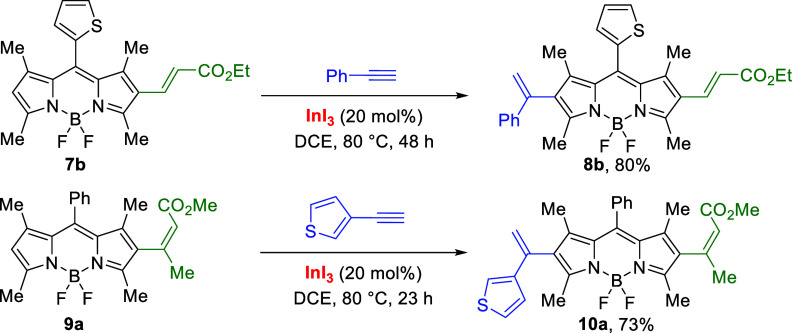
Synthesis of *push*–*pull* 2,6-Dialkenyl BODIPY Dyes

At this point, we explored the synthesis of unsymmetrical 2,6-dialkenyl BODIPYs by sequential hydroarylation reactions. In the event, the reaction of β-alkenyl BODIPY **7b** with phenylacetylene in the presence of InI_3_ (20 mol %) in DCE at 80 °C allowed the synthesis of unsymmetrical 2,6-dialkenyl BODIPY **8b** in 80% yield (Scheme 4). Analogously, we carried out the InI_3_-catalyzed intermolecular hydroarylation with BODIPY **9a** with 3-ethynylthiophene to give compound **10a** in 73% yield. It is remarkably to note that the electron-withdrawing effect of the alkenyl group at C2 does not affect the reactivity in the hydroarylation reaction at C6 in contrast with substitution at the *meso* position.

**Figure 1:**
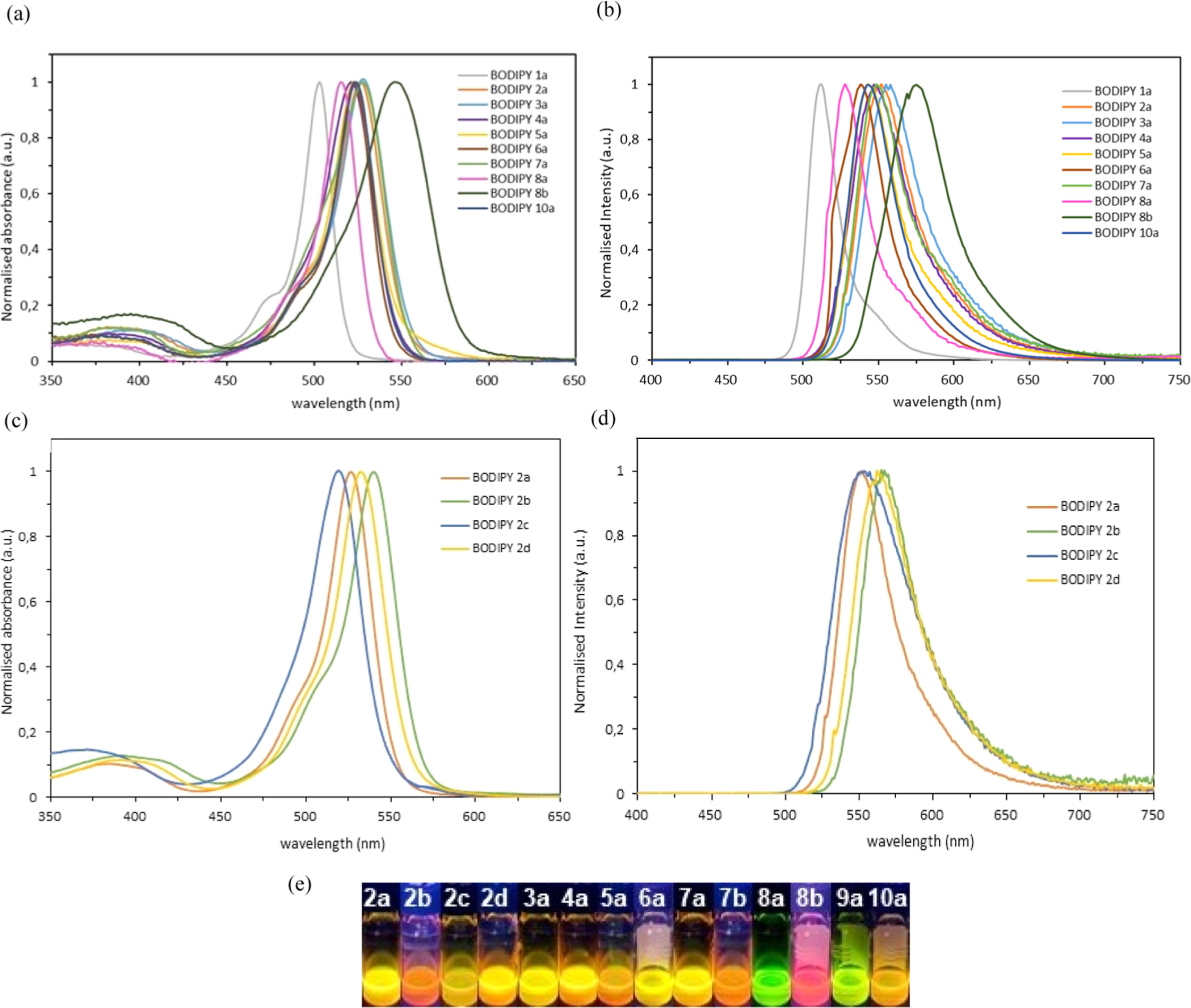
Normalized (a) absorbance and (b) emission spectra of BODIPY dyes **1a–8a**, **8b**, and **10a** in CHCl_3_ (7.5 × 10^–7^ M, excited at the respective under λ_max_). (c) Absorbance and (d) emission spectra of BODIPY dyes **2a–d** in CHCl_3_ (7.5 × 10^–7^ M, excited at the respective under λ_max_). (e) Photograph of the BODIPYs **2a–10a** and **7b–8b** under UV irradiation (λ = 365 nm).

The optical properties of synthesized fluorophores containing substituted alkenyl moieties at C2(6) positions of the BODIPYs **1a–d** were measured in CHCl_3_ (Table 2). In general, BODIPY dyes are characterized by a narrow absorption band assigned to the strong S_1_ ← S_0_ transition. Indeed, the absorption maxima of the linear β-alkenyl and branched β,β′-dialkenyl BODIPY dyes **2a–10a**, **2b–d**, and **7b–8b** induced a bathochromic shift (12–31 nm) by the electron-donating or electron-withdrawing substituents (Figure 1a,c). Besides, most compounds present high extinction coefficients (ε) such as the *meso*-phenyl-BODIPYs **3a** and **4a** (up to 88,200 M^–1^ cm^–1^) as the electron-donating ability at C2 and C6 positions increases, a required property in practical applications such as photodynamic therapy and photocatalysis.^6b,28^

**Table 2:** UV–Vis and PL Data of Compounds **2a–10a**, **2b–d**, and **7b–8b** in CHCl_3_

comp.^a^	λ_max_^Abs^ (nm) [ε × 10^3^(M^–1^ cm^–1^)]	λ_max_^PL^ (nm)	Stokes shift (nm)	Φ_F_^b^
**1a**	503 (74.4)	512	9	nd
**1b**	515 (66.5)	522	7	nd
**1c**	499 (54.4)	508	9	nd
**1d**	507 (47.4)	519	12	nd
**2a**	526 (64.9)	552	26	0.72
**2b**	540 (77.7)	565	25	0.14
**2c**	520 (38.4)	554	34	0.96
**2d**	532 (64.8)	562	30	0.49
**3a**	528 (83.3)	558	30	0.82
**4a**	521 (88.2)	547	26	0.72
**5a**	523 (79.2)	543	20	0.79
**6a**	523 (55.0)	538	15	0.38
**7a**	528 (56.1)	549	21	0.79
**7b**	541(43.2)	566	25	0.18
**8a**	515 (40.3)	528	13	0.69
**8b**	546 (48.5)	575	29	0.16
**9a**	514 (75.3)	526	12	0.61
**10a**	524 (51.6)	544	20	0.77

a All spectra were recorded in CHCl_3_ solutions at room temperature at 7.5 × 10^–7^ M for UV–vis and PL spectra, excited at the respective under λ_max_.

b Fluorescence quantum yields determined relative to rhodamine 6G in as standard (Φ_F_ = 0.94 in EtOH).^32^

2-Alkenyl and 2,6-dialkenyl BODIPYs also show narrow emission bandwidths covering the spectral range from 526 to 575 nm, exhibiting a more pronounced bathochromic effect (14–53 nm), related to the electron richness of the substituent on the alkenyl moiety (Figure 1b,d and Table 2). Interestingly, branched alkenyl BODIPY **2d** equipped with a 4-cyanophenyl group at the *meso* position also showed a bathochromic shift, that could be attributed to a stabilization of the LUMO level due to the electron-withdrawing effect of the cyano group.^29^

Besides, 2-alkenyl BODIPY **7b**, with extending conjugation containing an acrylate moiety, is bathochromically shifted (44 nm) related to **1b** due to its D-π-A character. Moreover, when a second alkenyl substituent is placed at the C6 position, the Stokes shift was increased to 53 nm, which confirmed the results from a strong internal charge transfer (ICT) effect in unsymmetrical BODIPY **8b** induced by greater *push*–*pull* electron movement.

Remarkably, the BODIPY dyes showed in general good-to-excellent quantum yields (up to Φ_F_ = 0.96), suggesting a very rigid structures (Table 2). The quenched fluorescence observed for **2b**, **7b**, and **8b** is basically due to the greater freedom of rotation of thien-2-yl group at the *meso* position, increasing the energy lost to nonradiative decay.^30^ In the case of **2d**, the electron-withdrawing effect of the cyano group could lead to a deactivation process through photoinduced electron-transfer (PET) from the electron-rich BODIPY core to the electron poor cyano moiety, reducing the quantum yield.^7,31^ Meanwhile, the low Φ_F_ of BODIPY **6a** possessing heavy atoms (bromine) could be attributed that might arise an intersystem crossing from an excited singlet state to an excited triplet state, induced by the halogens linked to the BODIPY core through the alkenyl moiety.^6^

In general, the BODIPYs equipped with substituted alkenyl groups showed a remarkable effect on the absorption and fluorescence properties. Indeed, these compounds exhibit greater photophysical properties compared to the reported BODIPY dyes containing a *p*-dimethylamino substituent at the α-position of the alkene moiety.^11d^ The molar absorption coefficients and quantum yield values tend to be excellent in some dyes. Therefore, compounds exhibit brightness when irradiated, as shown in Figure 1e, and show properties of great interest in fluorescence bioimaging.

## Conclusions

A new method for the synthesis of β-alkenyl-substituted BODIPYs by the indium(III)-catalyzed intermolecular alkyne hydroarylation reaction of *meso*-substituted BODIPYs has been developed. Depending on the different nature of the interaction of the alkyne to the indium(III) salt resulted in two different types of products. The double intermolecular hydroarylation reaction of phenylacetylene and analogues with *meso*-substituted-BODIPYs takes place through the electrophilic π-activation mode using indium triiodide as the catalyst, to provide branched 2,6-dialkenyl BODIPYs with Markovnikov regioselectivity. Conversely, when ethyl propiolate was used, the hydroarylation occurs through the σ-activation mode in the presence of indium trichloride as the catalyst, to give rise linear (*E*)-2-alkenyl BODIPYs coming from a β-addition. This atom-economical dual catalysis allowed the synthesis of *push*–*pull* dialkenyl BODIPY dyes with tuneable spectral properties. The majority of compounds synthesized have brilliant fluorescence with emissions that span from 526 to 575 nm with high quantum yields (0.14–0.96). Further research will be focused on exploring further functionalization, photophysical properties, and applications in bioimaging.

## Experimental Section

### General Methods

All reactions were carried out in dried glassware, under the argon atmosphere, using standard gastight syringes and septa. Dry toluene, DCE, CH_3_CN, and other commercially available reagents were used as received. Reaction temperatures refer to external bath temperatures. All indium(III) salts were used as received under argon in a glovebox system. The BODIPY substrates (**1a–d**) were prepared according to the literature.^19^ Reactions were monitored by thin-layer chromatography, in precoated silica gel foils, using UV light as the visualizing agent and ethanolic phosphomolybdic acid as the developing agent. Flash column chromatography was performed using 230–400 mesh silica gel. ^1^H NMR, ^19^F{H} NMR, and ^13^C{H} NMR spectra were recorded at room temperature in CDCl_3_ using a 300 or 500 MHz spectrometer and calibrated to the solvent peak. DEPT data were used to assign carbon types. Chemical shifts are reported in ppm (δ) relative to the solvent CDCl_3_ (δ_H_ 7.26 ppm and δ_C_ 77.1 ppm). ^19^F NMR are internally referenced with respect to CFCl_3_ (δ_F_ 0.0 ppm). Structural assignments were made with additional information from NOESY, COSY, HSQC, and HMBC experiments. Mass spectra were recorded on a Magnetic Sector EI spectrometer or a QSTAR ESI mass spectrometer, operating in the positive ionization mode. The IR spectra were recorded with attenuated total reflectance (ATR). Ultraviolet/visible (UV/vis) absorption and fluorescence spectra were recorded with standard 1 cm quartz cells. Emission spectra were recorded using a spectrofluorometer equipped with a pulsed xenon flash-lamp as a light source. Compounds were excited at their excitation maxima (band of lowest energy) to record the emission spectra. The concentration of the compound solutions (in CHCl_3_) was adjusted to 7.5 × 10^–7^ M. Fluorescence quantum yield (Φ_F_) values were determined by comparison with rhodamine 6G in ethanol as a reference (Φ_F_ = 0.94)^32^ using the equation below, where Φ_ST_ is the quantum yield of the reference, *m* is the slope of the line obtained from the best linear fit of the integrated fluorescence intensity versus absorbance data, and η is the refractive index of the solvent.
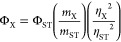


### General Procedure for the Indium(III)-Catalyzed Intermolecular Alkyne Hydroarylation Reaction with BODIPYs

A Schlenk tube equipped with a magnetic stir bar was charged with the indium salt (0.04 mmol, 0.2 equiv) inside a glovebox. Then, the corresponding BODIPY (0.2 mmol, 1 equiv), DCE (2 mL), and the corresponding alkyne (2.8 mmol, 14 equiv) were added. The reaction mixture was heated in an oil bath at 80 °C and monitored by TLC until the starting material was consumed. The reaction mixture was cooled to rt, and the solvent was removed under reduced pressure. The resulting crude was purified by flash chromatography on silica gel (EtOAc/hexanes) to afford after concentration, and high vacuum drying, the corresponding 2-alkenyl or 2,6-dialkyne BODIPYs.

#### 5,5-Difluoro-1,3,7,9-tetramethyl-10-phenyl-2,8-bis(1-phenylvinyl)-5*H*-4λ^4^,5λ^4^-dipyrrolo[1,2-*c*:2′,1′-*f*][1,3,2]diazaborinine (**2a**)

Following the general procedure, the reaction of **1a** (49.9 mg, 0.154 mmol) with InI_3_ (15.3 mg, 0.081 mmol) and phenylacetylene (0.24 mL, 2.16 mmol) in DCE (1.5 mL) was heated at 80 °C for 8 h. After purification by chromatography (2–4% EtOAc/hexanes), compound **2a** (73.2 mg, 90%) was obtained as a pink solid: mp = 106–107 °C; λ_max_^abs^ (CHCl_3_) = 526 nm (ε = 64,944 M^–1^ cm^–1^); λ_max_^em^ (CHCl_3_) = 552 nm; Φ_F_ (CHCl_3_) = 0.72; IR (ATR) ν_max_ = 2957, 2924, 2853, 1529, 1176 cm^–1^; ^1^H NMR (300 MHz, CDCl_3_) δ 7.43–7.49 (m, 3H), 7.34–7.36 (m, 2H), 7.26–7.31 (m, 10H), 5.85 (d, *J* = 1.5 Hz, 2H), 5.11 (d, *J* = 1.5 Hz, 2H), 2.40 (s, 6H), 1.23 (s, 6H); ^13^C{1H} NMR (75 MHz, CDCl_3_) δ 154.9 (2 × C), 141.9 (2 × C), 141.0 (C), 140.5 (2 × C), 140.2 (2 × C), 135.3 (2 × C), 133.2 (2 × C), 131.3 (C), 129.2 (2 × CH), 129.0 (CH), 128.5 (4 × CH), 128.1 (2 × CH), 127.8 (2 × CH), 126.3 (4 × CH), 117.3 (2 × CH_2_), 13.3 (2 × CH_3_), 12.8 (2 × CH_3_); ^19^F{1H} NMR (282 MHz, CDCl_3_) δ −145.80 (q, *J*_B–F_ = 32.5 Hz, 2F); HRMS (ESI) *m*/*z*: [M + Na]^+^ calculated for C_35_H_31_BF_2_N_2_Na, 551.2446, found, 551.2441.

#### 5,5-Difluoro-1,3,7,9-tetramethyl-2,8-bis(1-phenylvinyl)-10-(thiophen-2-yl)-5*H*-4λ^4^,5λ^4^-dipyrrolo[1,2-*c*:2′,1′-*f*][1,3,2]diazaborinine (**2b**)

Following the general procedure, the reaction of **1b** (40.1 mg, 0.121 mmol) with InI_3_ (12.0 mg, 0.024 mmol) and phenylacetylene (0.19 mL, 1.69 mmol) in DCE (1.5 mL) was heated at 80 °C for 8 h. After purification by chromatography (2% EtOAc/hexanes), compound **2b** (59.8 mg, 93%) was obtained as a pink solid: mp = 112–113 °C. λ_max_^abs^ (CHCl_3_) = 540 nm (ε = 77,698 M^–1^ cm^–1^); λ_max_^em^ (CHCl_3_) = 565 nm; Φ_F_ (CHCl_3_) = 0.14; IR (ATR) ν_max_ = 2962, 2918, 2851, 1259, 1023 cm^–1^; ^1^H NMR (300 MHz, CDCl_3_) δ 7.47 (dd, *J* = 1.3, 5.1 Hz, 1H), 7.26–7.32 (m, 10H), 7.10 (dd, *J* = 3.4, 5.1 Hz, 1H), 7.04 (dd, *J* = 1.2, 3.4 Hz, 1H), 5.87 (d, *J* = 1.5 Hz, 2H), 5.14 (d, *J* = 1.5 Hz, 2H), 2.40 (s, 6H), 1.44 (s, 6H); ^13^C{1H} NMR (75 MHz, CDCl_3_) δ 155.6 (2 × C), 140.8 (2 × C), 140.7 (2 × C), 140.1 (2 × C), 135.0 (2 × C), 134.1 (C), 133.5 (2 × C), 132.3 (C), 128.5 (4 × CH), 128.0 (CH), 127.9 (2 × CH), 127.6 (CH), 127.5 (CH), 126.3 (4 × CH), 117,4 (2 × CH_2_), 13.4 (2 × CH_3_), 12.0 (2 × CH_3_); ^19^F{1H} NMR (282 MHz, CDCl_3_) δ −145.74 (q, *J*_B–F_ = 32.6 Hz, 2F); HRMS (ESI) *m*/*z*: [M + Na]^+^ calculated for C_33_H_29_BF_2_N_2_SNa, 557.2010, found, 557.2001.

### Scale-Up Experiment for **2b**

A Schlenk tube equipped with a magnetic stir bar was charged with InI_3_ (120 mg, 0.242 mmol) inside a glovebox. Then, a solution of **1b** (400 mg, 1.21 mmol) in DCE (5.6 mL) and phenylacetylene (1.86 mL, 16.9 mmol) were added. The resulting mixture was heated in an oil bath at 80 °C for 8 h. The reaction mixture was cooled to rt, and the solvent was removed under reduced pressure. The residue was purified by flash chromatography on silica gel (2 to 8% EtOAc/hexanes) to afford after concentration, and high vacuum drying, **2b** (517.4 mg, 80%) as a pink solid.

#### 5,5-Difluoro-1,3,7,9,10-pentamethyl-2,8-bis(1-phenylvinyl)-5*H*-4λ^4^,5λ^4^-dipyrrolo[1,2-*c*:2′,1′-*f*][1,3,2]diazaborinine (**2c**)

Following the general procedure, the reaction of **1c** (40.1 mg, 0.153 mmol) with InI_3_ (15.1 mg, 0.031 mmol) and phenylacetylene (0.24 mL, 2.14 mmol) in DCE (1.5 mL) was heated at 80 °C for 24 h. After purification by chromatography (2% EtOAc/hexanes), compound **2c** (51.6 mg, 73%) was obtained as a pink solid: mp = 168–169 °C. λ_max_^abs^ (CHCl_3_) = 520 nm (ε = 38,421 M^–1^ cm^–1^); λ_max_^em^ (CHCl_3_) = 554 nm; Φ_F_ (CHCl_3_) = 0.96; IR (ATR) ν_max_ = 2956, 2920, 2851, 1544, 1193 cm^–1^; ^1^H NMR (300 MHz, CDCl_3_) δ 7.29–7.36 (m, 10H), 5.95 (d, *J* = 1.5 Hz, 2H), 5.18 (d, *J* = 1.5 Hz, 2H), 2.67 (s, 3H), 2.37 (s, 6H), 2.27 (s, 6H); ^13^C{1H} NMR (75 MHz, CDCl_3_) δ 153.1 (2 × C), 141.5 (C), 141.0 (2 × C), 140.3 (2 × C), 138.3 (2 × C), 133.0 (2 × C), 132.2 (2 × C), 128.5 (4 × CH), 127.9 (2 × CH), 126.3 (4 × CH), 117.4 (2 × CH_2_), 17.1 (2 × CH_3_), 15.5 (2 × CH_3_), 13.2 (CH_3_); ^19^F{1H} NMR (282 MHz, CDCl_3_) δ −146.21 (q, *J*_B–F_ = 32.8 Hz, 2F); HRMS (IE) *m*/*z*: [M]^+^ calculated for C_30_H_29_BF_2_N_2_, 466.2392, found, 466.2376.

#### 4-(5,5-Difluoro-1,3,7,9-tetramethyl-2,8-bis(1-phenylvinyl)-5*H*-4λ^4^,5λ^4^-dipyrrolo[1,2-*c*:2′,1′-*f*][1,3,2]diazaborinin-10-yl)benzonitrile (**2d**)

Following the general procedure, the reaction of **1d** (80.4 mg, 0.229 mmol) with InI_3_ (22.7 mg, 0.046 mmol) and phenylacetylene (0.35 mL, 3.21 mmol) in DCE (2 mL) was heated at 80 °C for 48 h. After purification by chromatography (4% EtOAc/hexanes), compound **2d** (13.7 mg, 11%) and 2-alkenyl BODIPY **11** (46.4 mg, 45%) were obtained as pink and orange solids, respectively.

##### Data for **2d**

mp = 92–93 °C; λ_max_^abs^ (CHCl_3_) = 532 nm (ε = 64,780 M^–1^ cm^–1^); λ_max_^em^ (CHCl_3_) = 562 nm; Φ_F_ (CHCl_3_) = 0.49; IR (ATR) ν_max_ = 2957, 2922, 2851, 2325, 1587, 1180 cm^–1^; ^1^H NMR (300 MHz, CDCl_3_) δ 7.79 (d, *J* = 8.0 Hz, 2H), 7.54 (d, *J* = 8.0 Hz, 2H), 7.28 (s, 10H), 5.86 (s, 2H), 5.11 (s, 2H), 2.40 (s, 6H), 1.20 (s, 6H); ^13^C{1H} NMR (75 MHz, CDCl_3_) δ 156.2 (C), 140.7 (2 × C), 140.4 (2 × C), 140.0 (2 × C), 139.8 (2 × C), 138.8 (C), 133.8 (C), 133.0 (2 × CH), 130.6 (C), 129.6 (2 × CH), 128.6 (4 × CH), 128.1 (2 × CH), 126.3 (4 × CH), 118.0 (2 × C), 117.6 (2 × CH_2_), 113.4 (2 × C), 13.5 (2 × CH_3_), 13.1 (2 × CH_3_); ^19^F{1H} NMR (282 MHz, CDCl_3_) δ −145.78 (q, *J*_B–F_ = 33.2 Hz, 2F); HRMS (ESI) *m*/*z*: [M + Na]^+^ calculated for C_36_H_30_BF_2_N_3_Na, 576.2399, found, 576.2390.

##### Data for **11**

mp = 77–78 °C; IR (ATR) ν_max_ = 2954, 2921, 2851,2338, 1462, 1377 cm^–1^; ^1^H NMR (300 MHz, CDCl_3_) δ 7.80 (d, *J* = 8.1 Hz, 2H), 7.50 (d, *J* = 8.0 Hz, 2H), 7.28 (s, 5H), 6.03 (s, 1H) 5.85 (s, 1H), 5.09 (s, 1H), 2.57 (s, 3H), 2.38 (s, 3H), 1.37 (s, 3H), 1.19 (s, 3H); ^13^C{1H} NMR (75 MHz, CDCl_3_) δ 156.7 (C), 156.0 (C), 142.6 (C), 140.7 (C), 140.2 (2 × C), 139.9 (2 × C), 139.7 (C), 138.7 (C), 132.9 (2 × CH), 129.5 (2 × CH), 129.4 (C), 128.5 (2 × CH), 128.0 (CH), 126.3 (2 × CH), 121.9 (CH), 118.1 (C), 117.6 (CH_2_), 113.3 (C), 14.7 (2 × CH_3_), 13.4 (CH_3_), 13.0 (CH_3_); ^19^F{1H} NMR (282 MHz, CDCl_3_) δ −145.96 (q, *J*_B–F_ = 32.3 Hz, 2F); HRMS (ESI) *m*/*z*: [M + Na]^+^ calculated for C_28_H_24_BF_2_N_3_Na, 474.1929, found, 474.1918.

#### 5,5-Difluoro-2,8-bis(1-(4-methoxyphenyl)vinyl)-1,3,7,9-tetramethyl-10-phenyl-5*H*-4λ^4^,5λ^4^-dipyrrolo[1,2-*c*:2′,1′-*f*][1,3,2]diazaborinine (**3a**)

Following the general procedure, the reaction of **1a** (79.9 mg, 0.247 mmol) with InI_3_ (24.5 mg, 0.05 mmol) and 1-ethynyl-4-methoxybenzene (0.45 mL, 3.46 mmol) in DCE (2 mL) was heated at 80 °C for 24 h. After purification by chromatography (1–3% EtOAc/hexanes), compound **3a** (57.9 mg, 40%) was obtained as a pink solid: mp = 107–108 °C; λ_max_^abs^ (CHCl_3_) = 528 nm (ε = 83,256 M^–1^ cm^–1^); λ_max_^em^ (CHCl_3_) = 558 nm; Φ_F_ (CHCl_3_) = 0.82; IR (ATR) ν_max_ = 2956, 2925, 2837, 1533, 1172 cm^–1^; ^1^H NMR (300 MHz, CDCl_3_) δ 7.36–7.39 (m, 3H), 7.26–7.28 (m, H), 7.14–7.18 (m, 4H), 6.73 (d, *J* = 8.6 Hz, 4H), 5.67 (s, 2H), 4.92 (s, 2H), 3.72 (s, 6H), 2.32 (s, 6H), 1.15 (s, 6H); ^13^C{1H} NMR (75 MHz, CDCl_3_) δ 160.0 (2 × C), 155.4 (C), 142.3 (C), 140.9 (C), 140.8 (4 × C), 135.9 (C), 134.0 (C), 133.3 (4 × C), 131.8 (C), 129.7 (2 × CH), 129.5 (CH), 128.6 (2 × CH), 128.0 (4 × CH), 115.8 (2 × CH_2_), 114.3 (4 × CH), 55.8 (2 × CH_3_), 13.9 (2 × CH_3_), 13.3 (2 × CH_3_); ^19^F{1H} NMR (282 MHz, CDCl_3_) δ −145.83 (q, *J*_B–F_ = 32.9 Hz, 2F); HRMS (ESI) *m*/*z*: [M + Na]^+^ calculated for C_37_H_35_BF_2_N_2_O_2_Na, 611.2657, found, 611.2650.

#### 5,5-Difluoro-1,3,7,9-tetramethyl-10-phenyl-2,8-bis(1-(thiophen-3-yl)vinyl)-5*H*-4λ^4^,5λ^4^-dipyrrolo[1,2-*c*:2′,1′-*f*][1,3,2]diazaborinine (**4a**)

Following the general procedure, the reaction of **1a** (79.8 mg, 0.247 mmol) with InI_3_ (24.5 mg, 0.05 mmol) and 3-ethynylthiophene (0.34 mL, 3.46 mmol) in DCE (2 mL) was heated at 80 °C for 23 h. After purification by chromatography (1–3% EtOAc/hexanes), compound **4a** (83.8 mg, 63%) was obtained as a pink solid: mp = 98–100 °C; λ_max_^abs^ (CHCl_3_) = 521 nm (ε = 88,163 M^–1^ cm^–1^); λ_max_^em^ (CHCl_3_) = 547 nm; Φ_F_ (CHCl_3_) = 0.72; IR (ATR) ν_max_ = 2956, 2929, 2853, 1536, 1178 cm^–1^; ^1^H NMR (300 MHz, CDCl_3_) δ 7.35–7.42 (m, 3H), 7.23–7.27 (m, 2H), 7.16–7.19 (m, 2H), 7.11–7.13 (m, 2H), 6.83–6.85 (m, 2H), 5.71 (s, 2H), 4.95 (s, 2H), 2.37 (s, 6H), 1.17 (s, 6H); ^13^C{1H} NMR (75 MHz, CDCl_3_) δ 155.2 (2 × C), 142.7 (C), 142.5 (C), 140.7 (2 × C), 136.3 (2 × C), 135.7 (2 × C), 133.9 (C), 131.7 (C), 129.7 (2 × CH), 129.7 (C), 129.6 (CH), 128.6 (2 × CH), 128.5 (C), 126.6 (2 × CH), 125.9 (2 × CH), 123.2 (2 × CH), 116.5 (2 × CH_2_), 13.8 (2 × CH_3_), 13.3 (2 × CH_3_); ^19^F{1H} NMR (282 MHz, CDCl_3_) δ −145.88 (q, *J*_B–F_ = 32.7 Hz, 2F); HRMS (ESI) *m*/*z*: [M + Na]^+^ calculated for C_31_H_27_BF_2_N_2_S_2_Na, 563.1574, found, 563.1568.

#### 2,8-Bis(1-(3,5-bis(trifluoromethyl)phenyl)vinyl)-5,5-difluoro-1,3,7,9-tetramethyl-10-phenyl-5*H*-4λ^4^,5λ^4^-dipyrrolo[1,2-*c*:2′,1′-*f*][1,3,2]diazaborinine (**5a**)

Following the general procedure, the reaction of **1a** (44.8 mg, 0.139 mmol) with InI_3_ (13.8 mg, 0.028 mmol) and 1-ethynyl-3,5-bis(trifluoromethyl)benzene (0.34 mL, 1.94 mmol) in DCE (2 mL) was heated at 80 °C for 6 h. After purification by chromatography (2% EtOAc/hexanes), compound **5a** (102.1 mg, 92%) was obtained as a pink solid: mp = 106–107 °C; λ_max_^abs^ (CHCl_3_) = 523 nm (ε = 79,174 M^–1^ cm^–1^); λ_max_^em^ (CHCl_3_) = 543 nm; Φ_F_ (CHCl_3_) = 0.79; IR (ATR) ν_max_ = 2926,2854, 1535, 1279, 1131, 1136 cm^–1^; ^1^H NMR (300 MHz, CDCl_3_) δ 7.78 (s, 2H), 7.72 (s, 4H), 7.46–7.54 (m, 3H), 7.35–7.38 (m, 2H), 6.00 (s, 2H), 5.35 (s, 2H), 2.44 (s, 6H), 1.21 (s, 6H); ^13^C{1H} NMR (75 MHz, CDCl_3_) δ 154.8 (2 × C), 143.0 (C), 142.4 (2 × C), 141.0 (2 × C), 138.7 (2 × C), 134.8 (2 × C), 132.0 (q, ^2^*J*_CF_ = 33.4 Hz, 4 × C), 131.6 (C), 131.4 (C), 129.4 (2 × CH), 129.3 (CH), 128.0 (C), 127.8 (2 × CH), 126.2 (4 × CH), 123.2 (q, ^1^*J*_CF_ = 272.3 Hz, 4 × CF_3_), 121.6 (2 × CH), 120.9 (2 × CH_2_), 13.4 (CH_3_), 13.0 (3 × CH_3_); ^19^F{1H} NMR (282 MHz, CDCl_3_) δ −62.86 (s, 4CF_3_), −145.73 (q, *J*_B–F_ = 33.1 Hz, 2F); HRMS (ESI) *m*/*z*: [M + Na]^+^ calculated for C_39_H_27_BF_14_N_2_Na, 823.1941, found, 823.1941.

#### 2,8-Bis((*Z*)-2-bromo-1-phenylvinyl)-5,5-difluoro-1,3,7,9-tetramethyl-10-phenyl-5*H*-4λ^4^,5λ^4^-dipyrrolo[1,2-*c*:2′,1′-*f*][1,3,2]diazaborinine (**6a**)

Following the general procedure, the reaction of **1a** (20.1 mg, 0.062 mmol) with InI_3_ (6.1 mg, 0.012 mmol) and (bromoethynyl)benzene (156.4 mg, 0.86 mmol) in DCE (0.7 mL) was heated at 80 °C for 3 h. After purification by chromatography (3% EtOAc/hexanes), compound **6a** (31.3 mg, 74%) was obtained as a pink solid: mp = 105–106 °C; λ_max_^abs^ (CHCl_3_) = 523 nm (ε = 54,973 M^–1^ cm^–1^); λ_max_^em^ (CHCl_3_) = 538 nm; Φ_F_ (CHCl_3_) = 0.38; IR (ATR) ν_max_ = 2955, 2923, 2851, 1461, 1377 cm^–1^; ^1^H NMR (300 MHz, CDCl_3_) δ 7.38–7.49 (m, 5H), 7.27–7.31 (m, 6H), 7.20–7.24 (m, 4H), 6.95 (s, 2H), 2.41 (s, 6H), 1.23 (s, 6H); ^13^C{1H} NMR (75 MHz, CDCl_3_) δ 154.6 (2 × C), 142.2 (C), 141.0 (C), 139.6 (C), 139.6 (C), 139.0 (2 × C), 138.9 (2 × C), 135.0 (2 × C), 131.5 (C), 130.8 (C), 129.4 (CH), 129.2 (CH), 129.1 (CH), 128.8 (CH), 128.37 (CH), 128.19 (CH), 128.1 (CH), 128.0 (4 × CH), 126.4 (4 × CH), 109.4 (CH), 109.4 (CH), 13.4 (2 × CH_3_), 12.9 (2 × CH_3_); ^19^F{1H} NMR (282 MHz, CDCl_3_) δ −145.67 (q, *J*_B–F_ = 33.1 Hz, 2F); HRMS (ESI) *m*/*z*: [M + Na]^+^ calculated for C_35_H_29_BF_2_N_2_Br_2_Na, 707.0656, found, 707.0653.

#### Ethyl (*E*)-3-(5,5-difluoro-1,3,7,9-tetramethyl-10-phenyl-5*H*-4λ^4^,5λ^4^-dipyrrolo[1,2-*c*:2′,1′-*f*][1,3,2]diazaborinin-2-yl)acrylate (**7a**)

Following the general procedure, the reaction of **1a** (44.8 mg, 0.138 mmol) with InCl_3_ (6.1 mg, 0.028 mmol) and ethyl propiolate (0.20 mL, 1.93 mmol) in DCE (2 mL) was heated at 80 °C for 48 h. After purification by chromatography (5–20% EtOAc/hexanes), compound **7a** (23.4 mg, 40%) was obtained as a pink solid: mp = 155–156 °C; λ_max_^abs^ (CHCl_3_) = 528 nm (ε = 56,128 M^–1^ cm^–1^); λ_max_^em^ (CHCl_3_) = 549 nm; Φ_F_ (CHCl_3_) = 0.79; IR (ATR) ν_max_ = 2955, 2919, 2851, 1711, 1540, 1166 cm^–1^; ^1^H NMR (300 MHz, CDCl_3_) δ 7.55 (d, *J* = 16.5 Hz, 1H), 7.45–7.47 (m, 3H), 7.21–7.26 (m, 2H), 6.01 (s, 1H), 5.98 (d, *J* = 16.5 Hz, 1H), 4.17 (q, *J* = 7.1 Hz, 2H), 2.65 (s, 3H), 2.53 (s, 3H), 1.40 (s, 3H), 1.34 (s, 3H), 1.25 (t, *J* = 7.2 Hz, 3H); ^13^C{1H} NMR (75 MHz, CDCl_3_) δ 167.6 (C), 158.5 (C), 154.45 (C), 145.4 (C), 142.4 (C), 140.5 (C), 135.8 (CH), 134.7 (C), 132.7 (C), 130.6 (C), 129.3 (2 × CH), 129.3 (CH), 128.0 (2 × CH), 125.0 (C), 122.7 (CH), 117.5 (CH), 60.4 (CH_2_), 14.8 (CH_3_), 14.7 (CH_3_), 14.4 (CH_3_), 14.0 (CH_3_), 12.7 (CH_3_); ^19^F{1H} NMR (282 MHz, CDCl_3_) δ −144.94 (q, *J*_B–F_ = 32.9 Hz, 2F); HRMS (ESI) *m*/*z*: [M + Na]^+^ calculated for C_24_H_25_BF_2_N_2_O_2_Na, 445.1875, found, 445.1869.

#### Ethyl (*E*)-3-(5,5-difluoro-1,3,7,9-tetramethyl-10-(thiophen-2-yl)-5*H*-4λ^4^,5λ^4^-dipyrrolo[1,2-*c*:2′,1′-*f*][1,3,2]diazaborinin-2-yl)acrylate (**7b**)

Following the general procedure, the reaction of **1b** (40.1 mg, 0.121 mmol) with InCl_3_ (5.4 mg, 0.024 mmol) and ethyl propiolate (0.17 mL, 1.69 mmol) in DCE (2 mL) was heated at 80 °C for 48 h. After purification by chromatography (3–15% EtOAc/hexanes), compound **7b** (29.2 mg, 56%) was obtained as a pink solid: mp = 155–156 °C; λ_max_^abs^ (CHCl_3_) = 541 nm (ε = 43,246 M^–1^ cm^–1^); λ_max_^em^ (CHCl_3_) = 566 nm; Φ_F_ (CHCl_3_) = 0.18; IR (ATR) ν_max_ = 2956, 2924, 2852, 1708, 1539, 1284, 1164 cm^–1^; ^1^H NMR (300 MHz, CDCl_3_) δ 7.61 (d, *J* = 16.3 Hz, 1H), 7.53 (d, *J* = 4.8 Hz, 1H), 7.16 (t, *J* = 4.1 Hz, 1H), 7.01 (d, *J* = 3.2 Hz, 1H), 6.08 (s, 1H), 6.03 (d, *J* = 16.3 Hz, 1H), 4.23 (q, *J* = 7.1 Hz, 2H), 2.69 (s, 3H), 2.58 (s, 3H), 1.65 (s, 3H), 1.61 (s, 3H), 1.31 (t, *J* = 7.2 Hz, 3H); ^13^C{1H} NMR (75 MHz, CDCl_3_) δ 167.5 (C), 159.2 (C), 155.0 (C), 145.7 (C), 140.7 (C), 135.7 (CH), 134.8 (C), 134.3 (2 × C), 131.4 (C), 128.2 (CH), 127.9 (CH), 127.8 (CH), 125.3 (C), 123.0 (CH), 117.8 (CH), 60.4 (CH_2_), 14.9 (CH_3_), 14.4 (CH_3_), 14.1 (CH_3_), 13.9 (CH_3_), 12.0 (CH_3_); ^19^F{1H} NMR (319 MHz, CDCl_3_) δ −144.85 (q, *J*_B–F_ = 32.4 Hz, 2F); HRMS (ESI) *m*/*z*: [M + Na]^+^ calculated for C_22_H_23_BF_2_N_2_O_2_SNa, 451.1439, found, 451.1431.

#### Methyl (*Z*)-3-(5,5-difluoro-1,3,7,9-tetramethyl-10-phenyl-5*H*-4λ^4^,5λ^4^-dipyrrolo[1,2-*c*:2′,1′-*f*][1,3,2]diazaborinin-2-yl)-3-phenylacrylate (**8a**)

Following the general procedure, the reaction of **1a** (60.1 mg, 0.185 mmol) with InCl_3_ (8.2 mg, 0.037 mmol) and ethyl 3-phenylpropiolate (0.38 mL, 2.59 mmol) in DCE (2 mL) was heated at 80 °C for 24 h. Then, the temperature was raised to 100 °C, and the reaction was left for 2 more days. After purification by chromatography (5–20% EtOAc/hexanes), compound **8a** (37.2 mg, 42%) was obtained as a pink solid: mp = 101–102 °C; λ_max_^abs^ (CHCl_3_) = 515 nm (ε = 40,299 M^–1^ cm^–1^); λ_max_^em^ (CHCl_3_) = 528 nm; Φ_F_ (CHCl_3_) = 0.69; IR (ATR) ν_max_ = 3059, 2951, 1722, 1615, 1157 cm^–1^; ^1^H NMR (300 MHz, CDCl_3_) δ 7.43–7.47 (m, 3H), 7.29–7.37 (m, 7H), 6.49 (s, 1H), 6.01 (s, 1H), 3.68 (s, 3H), 2.58 (s, 3H), 2.34 (s, 3H), 1.39 (s, 3H), 1.11 (s, 3H); ^13^C{1H} NMR (75 MHz, CDCl_3_) δ 165.7 (C), 155.9 (C), 154.4 (C), 148.7 (2 × C), 143.5 (C), 141.9 (C), 140.4 (C), 139.6 (2 × C), 135.0 (2 × C), 129.7 (CH), 129.2 (CH), 129.1 (CH), 129.0 (CH), 128.8 (2 × CH), 128.2 (CH), 128.1 (CH), 128.0 (CH), 127.4 (2 × CH), 119.7 (CH), 49.9 (CH_3_), 14.7 (CH_3_), 14.5 (CH_3_), 13.3 (CH_3_), 12.8 (CH_3_); ^19^F{1H} NMR (282 MHz, CDCl_3_) δ −146.00 (dq, *J*_F–F_ = 253.9 Hz, *J*_B–F_ = 33.0 Hz, 2F); HRMS (ESI) *m*/*z*: [M + Na]^+^ calculated for C_29_H_27_BF_2_N_2_O_2_Na, 507.2031, found, 507.2028.

#### Methyl (*Z*)-3-(5,5-difluoro-1,3,7,9-tetramethyl-10-phenyl-5*H*-4λ^4^,5λ^4^-dipyrrolo[1,2-*c*:2′,1′-*f*][1,3,2]diazaborinin-2-yl)but-2-enoate (**9a**)

Following the general procedure, the reaction of **1a** (80.1 mg, 0.247 mmol) with InCl_3_ (10.9 mg, 0.049 mmol) and methyl but-2-ynoate (0.35 mL, 3.46 mmol) in DCE (2.5 mL) was heated at 80 °C for 24 h. Then, the temperature was raised to 100 °C, and the reaction was left for 2 more days. After purification by chromatography (3–10% EtOAc/hexanes), compound **9a** (37.5 mg, 36%) was obtained as a pink solid: mp = 134–135 °C; λ_max_^abs^ (CHCl_3_) = 514 nm (ε = 75,310 M^–1^ cm^–1^); λ_max_^em^ (CHCl_3_) = 526 nm; Φ_F_ (CHCl_3_) = 0.61; IR (ATR) ν_max_ = 2954, 2921, 2851, 1783, 1542, 1462 cm^–1^; ^1^H NMR (300 MHz, CDCl_3_) δ 7.37–7.42 (m, 3H), 7.25–7.29 (m, 1H), 7.18–7.22 (m, 1H), 5.96 (q, *J* = 1.4 Hz, 1H), 5.89 (s, 1H), 3.50 (s, 3H), 2.47 (s, 3H), 2.36 (s, 3H), 1.90 (t, *J* = 1.3 Hz, 3H), 1.29 (s, 3H), 1.15 (s, 3H); ^13^C{1H} NMR (75 MHz, CDCl_3_) δ 165.3 (C), 155.3 (C), 152.8 (C), 148.5 (2 × C), 143.0 (C), 141.6 (C), 138.2 (C), 135.1 (2 × C), 145.1 (C), 129.1 (2 × CH), 129.1 (CH), 128.9 (CH), 128.2 (CH), 128.0 (CH), 121.2 (CH), 51.2 (CH_3_), 26.3 (CH_3_), 14.6 (CH_3_), 14.3 (CH_3_), 13.1 (CH_3_), 12.5 (CH_3_); ^19^F{1H} NMR (282 MHz, CDCl_3_) δ −146.12 (dq, *J*_F–F_ = 308.9, *J*_B–F_ = 33.2 Hz, 2F); HRMS (ESI) *m*/*z*: [M + Na]^+^ calculated for C_24_H_25_BF_2_N_2_O_2_Na, 445.1875, found, 445.1876.

#### Ethyl (*E*)-3-(5,5-difluoro-1,3,7,9-tetramethyl-8-(1-phenylvinyl)-10-(thiophen-2-yl)-5*H*-4λ^4^,5λ^4^-dipyrrolo[1,2-*c*:2′,1′-*f*][1,3,2]diazaborinin-2-yl)acrylate (**8b**)

Following the general procedure, the reaction of **7b** (24.2 mg, 0.058 mmol) with InI_3_ (5.6 mg, 0.012 mmol) and phenylacetylene (0.090 mL, 0.82 mmol) in DCE (1 mL) was heated at 80 °C for 48 h. After purification by chromatography (2–5% EtOAc/hexanes), compound **8b** (23.9 mg, 80%) was obtained as a pink solid: mp = 96–97 °C; λ_max_^abs^ (CHCl_3_) = 546 nm (ε = 48,515 M^–1^ cm^–1^); λ_max_^em^ (CHCl_3_) = 575 nm; Φ_F_ (CHCl_3_) = 0.16; IR (ATR) ν_max_ = 2955, 2923, 2852, 1733, 1532, 1461 cm^–1^; ^1^H NMR (300 MHz, CDCl_3_) δ 7.63 (d, *J* = 16.3 Hz, 1H), 7.53 (dd, *J* = 1.1, 5.1 Hz, 1H), 7.27–7.33 (m, 5H), 7.13–7.16 (m, 1H), 7.04 (dd, *J* = 1.2, 3.5 Hz, 1H), 6.08 (d, *J* = 16.3 Hz, 1H), 5.89 (d, *J* = 1.3 Hz, 1H), 5.14 (d, *J* = 1.25 Hz, 1H), 4.25 (q, *J* = 7.1 Hz, 2H), 2.71 (s, 3H), 2.40 (s, 3H), 1.67 (s, 3H), 1.46 (s, 3H), 1.32 (t, *J* = Hz, 3H);^13^C{1H} NMR (75 MHz, CDCl_3_) δ 167.5 (C), 158.8 (C), 155.0 (C), 142.5 (C), 140.7 (C), 140.3 (2 × C), 139.6 (2 × C), 135.7 (CH), 134.8 (C), 134.5 (C), 128.6 (2 × CH), 128.5 (C), 128.2 (CH), 128.1 (CH), 127.9 (CH), 127.8 (CH), 126.9 (C), 126.3 (CH), 126.2 (2 × CH), 117.8 (CH_2_), 60.4 (CH_2_), 14.4 (CH_3_), 14.1 (CH_3_), 13.7 (CH_3_), 12.2 (CH_3_), 12.1 (CH_3_); ^19^F{1H} NMR (282 MHz, CDCl_3_) δ −144.59 (q, *J*_B–F_ = 31.7 Hz, 2F); HRMS (ESI) *m*/*z*: [M + Na]^+^ calculated for C_30_H_29_BF_2_N_2_O_2_SNa, 553.1909, found, 553.1890.

#### Methyl (*Z*)-3-(5,5-difluoro-1,3,7,9-tetramethyl-10-phenyl-8-(1-(thiophen-3-yl)vinyl)-5*H*-4λ^4^,5λ^4^-dipyrrolo[1,2-*c*:2′,1′-*f*][1,3,2]diazaborinin-2-yl)but-2-enoate (**10a**)

Following the general procedure, the reaction of **9a** (29.0 mg, 0.069 mmol) with InI_3_ (6.8 mg, 0.014 mmol) and 3-ethynylthiophene (0.10 mL, 0.96 mmol) in DCE (1 mL) was heated at 80 °C for 23 h. After purification by chromatography (3–10% EtOAc/hexanes), compound **10a** (26.3 mg, 73%) was obtained as a pink solid: mp = 102–104 °C; λ_max_^abs^ (CHCl_3_) = 524 nm (ε = 51,560 M^–1^ cm^–1^); λ_max_^em^ (CHCl_3_) = 544 nm; Φ_F_ (CHCl_3_) = 0.77; IR (ATR) ν_max_ = 2955, 2922, 2851, 1536, 1463 cm^–1^; ^1^H NMR (300 MHz, CDCl_3_) δ 7.36–7.41 (m, 3H), 7.27–7.32 (m, 1H), 7.22–7.26 (m, 1H), 7.17–7.19 (m, 1H), 7.10–7.12 (m, 1H), 6.84 (dd, *J* = 1.3, 3.0 Hz, 1H), 5.97 (q, *J* = 1.5 Hz, 1H), 5.71 (d, *J* = 1.5 Hz, 1H), 4.93 (d, *J* = 1.5 Hz, 1H), 3.52 (s, 3H), 2.38 (s, 3H), 2.36 (s, 3H), 1.92 (s, 3H), 1.16 (s, 3H), 1.15 (s, 3H); ^13^C{1H} NMR (75 MHz, CDCl_3_) δ 165.4 (C), 154.3 (C), 153.1 (C), 148.5 (C), 142.2 (C), 141.8 (C), 140.0 (C), 138.3 (C), 135.9 (C), 135.2 (2 × C), 133.2 (C), 132.3 (C), 131.0 (C), 129.1 (CH), 129.1 (CH), 129.0 (CH), 128.2 (CH), 128.1 (CH), 126.0 (CH), 125.4 (CH), 122.6 (CH), 121.3 (CH), 115.9 (CH_2_), 51.2 (CH_3_), 26.2 (2 × CH_3_), 13.1 (CH_3_), 12.7 (CH_3_), 12.6 (CH_3_); ^19^F{1H} NMR (282 MHz, CDCl_3_) δ −145.97 (dq, *J*_F–F_ = 262.9, *J*_B–F_ = 33.2 Hz, 2F); HRMS (ESI) *m*/*z*: [M + Na]^+^ calculated for C_30_H_29_BF_2_N_2_O_2_SNa, 553.1909, found, 553.1909.

## Data Availability

The data underlying this study are available in the published article and its Supporting Information.

## Supporting Information Available

Copies of ^1^H NMR, ^13^C NMR, and ^19^F NMR spectra of all compounds; NOE and 2D NMR spectra for compounds **6a**, **7a**, **8a**, and **9a**; and UV–vis and emission spectra for all compounds (PDF)
