# Supplementary material for: Synthesis and Photophysical Properties of β-Alkenyl-Substituted BODIPY Dyes by Indium(III)-Catalyzed Intermolecular Alkyne Hydroarylation
Source: J Org Chem. 2024 Mar 19;89(7):4702–11. doi: 10.1021/acs.joc.3c02951 (PMC11002825; doi:10.1021/acs.joc.3c02951)

Supporting Information

for

Synthesis and photophysical properties of β-alkenyl-substituted BODIPY dyes by indium(III)-catalyzed intermolecular alkyne hydroarylation

Ana Da Lama, José Pérez Sestelo, Luis A. Sarandeses,* and M. Montserrat Martínez *

CICA – Centro Interdisciplinar de Química e Bioloxía and Departamento de Química, Universidade da Coruña, 15071 A Coruña, Spain. Phone: (+34) 881012165. E-mail: monserrat.martinez.cebeira@udc.es, luis.sarandeses@udc.es

Table of Contents

Figure S1. Absorption and Emission spectra of BODIPY **1a** registered in $CHCl_3$……….....……S1

Figure S2. Absorption and Emission spectra of BODIPY **1b** registered in $CHCl_3$……….....……S1

Figure S3. Absorption and Emission spectra of BODIPY **1c** registered in $CHCl_3$……….....……S2

Figure S4. Absorption and Emission spectra of BODIPY **1d** registered in $CHCl_3$……....………S2

Figure S5. Absorption and Emission spectra of BODIPY **2a** registered in $CHCl_3$……….....……S3

Figure S6. Absorption and Emission spectra of BODIPY **2b** registered in $CHCl_3$……….....……S3

Figure S7. Absorption and Emission spectra of BODIPY **2c** registered in $CHCl_3$……..........……S4

Figure S8. Absorption and Emission spectra of BODIPY **2d** registered in $CHCl_3$……........……S4

Figure S9. Absorption and Emission spectra of BODIPY **3a** registered in $CHCl_3$……….....……S5

Figure S10. Absorption and Emission spectra of BODIPY **4a** registered in $CHCl_3$……...…...…S5

Figure S11. Absorption and Emission spectra of BODIPY **5a** registered in $CHCl_3$……......……S6

Figure S12. Absorption and Emission spectra of BODIPY **6a** registered in $CHCl_3$……......……S6

Figure S13. Absorption and Emission spectra of BODIPY **7a** registered in $CHCl_3$……......……S7

Figure S14. Absorption and Emission spectra of BODIPY **7b** registered in $CHCl_3$……......……S7

Figure S15. Absorption and Emission spectra of BODIPY **8a** registered in $CHCl_3$……….……S8

Figure S16. Absorption and Emission spectra of BODIPY **9a** registered in $CHCl_3$……......……S8

Figure S17. Absorption and Emission spectra of BODIPY **8b** registered in $CHCl_3$……......……S9

Figure S18. Absorption and Emission spectra of BODIPY **10a** registered in $CHCl_3$……...……S9

Copies of the ^{1}H NMR, ^{13}C NMR ^{19}F-NMR, nOe and 2D NMR spectra...,,...............S10

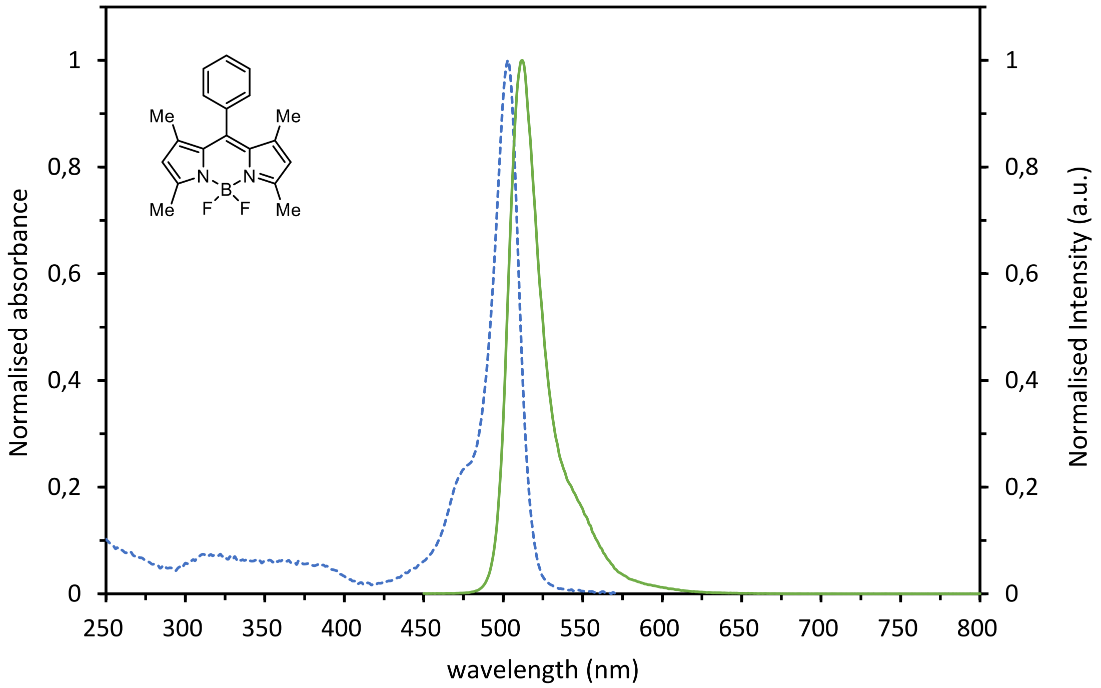

Figure S1. Absorption and Emission spectra of BODIPY **1a** registered in $CHCl_3$ ([**1a**]= $7.5 \cdot 10^{-7}$ M). λ_{max}(abs)= 503 nm; λ_{max}(em)= 512 nm.

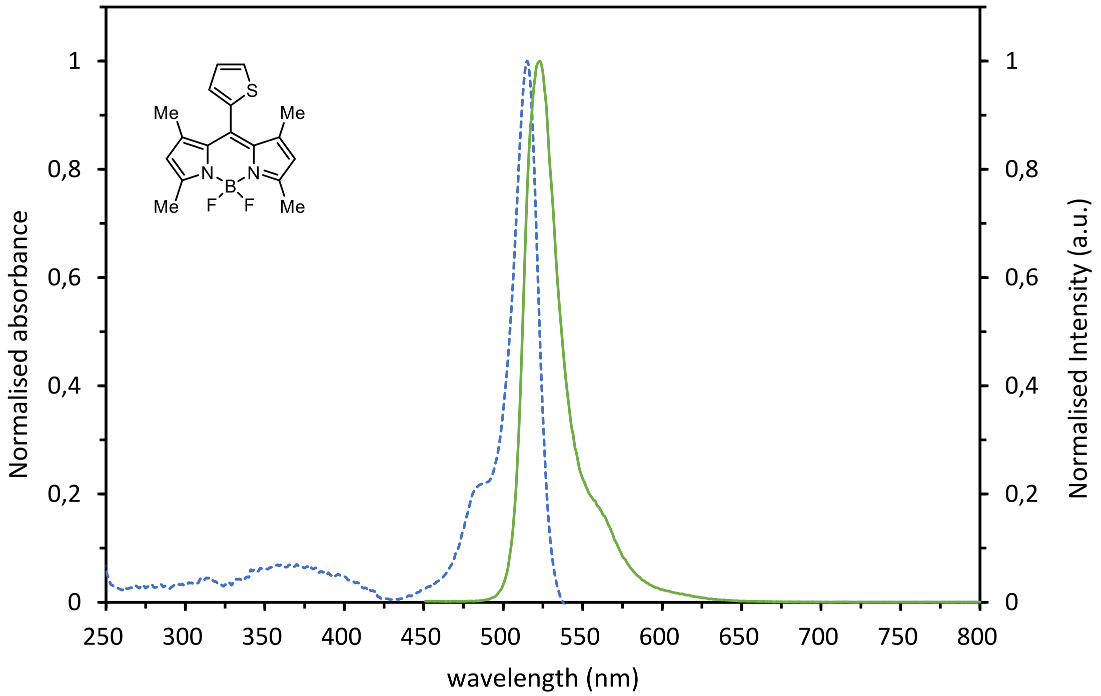

Figure S2. Absorption and Emission spectra of BODIPY **1b** registered in $CHCl_3$ ([**1b**]= $7.5 \cdot 10^{-7}$ M). λ_{max}(abs)= 515 nm; λ_{max}(em)= 522 nm.

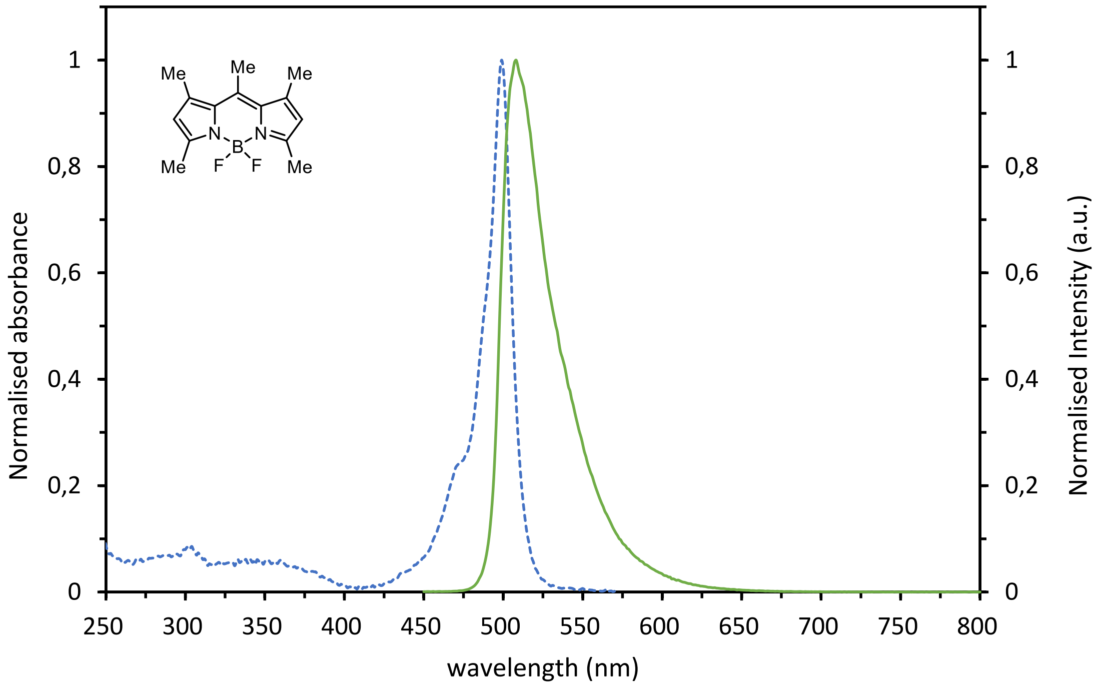

Figure S3. Absorption and Emission spectra of BODIPY **1c** registered in $CHCl_3$ ([**1c**]= $7.5 \cdot 10^{-7}$ M). λ_{max}(abs)= 499 nm; λ_{max}(em)= 508 nm.

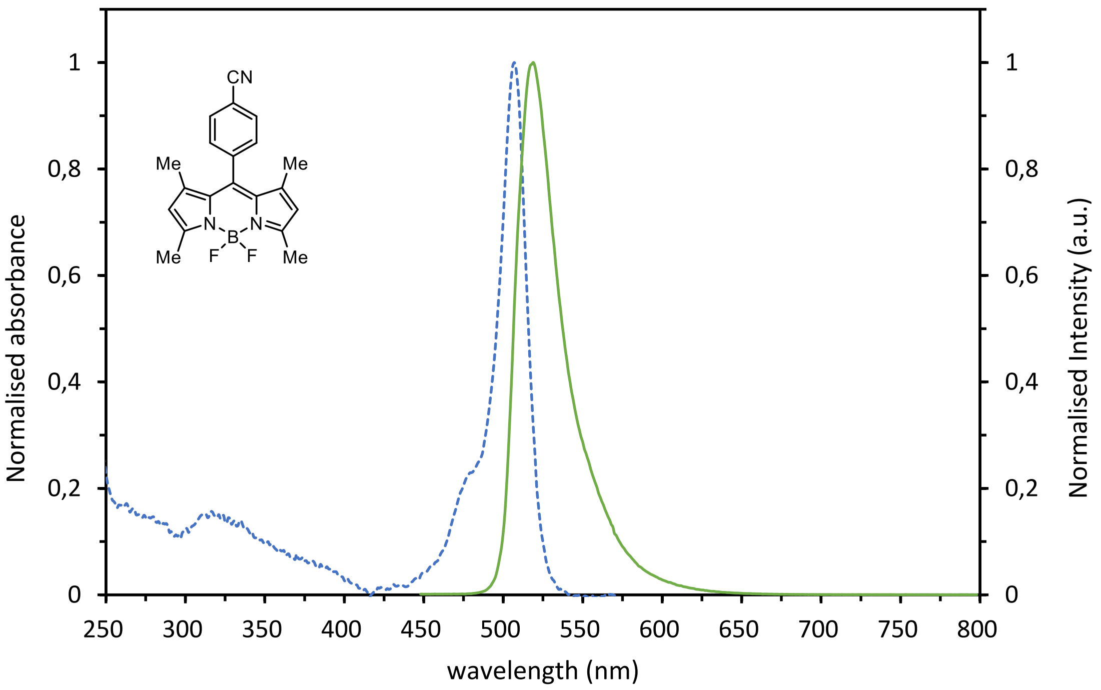

Figure S4. Absorption and Emission spectra of BODIPY **1d** registered in $CHCl_3$ ([**1d**]= $7.5 \cdot 10^{-7}$ M). λ_{max}(abs)= 507 nm; λ_{max}(em)= 519 nm.

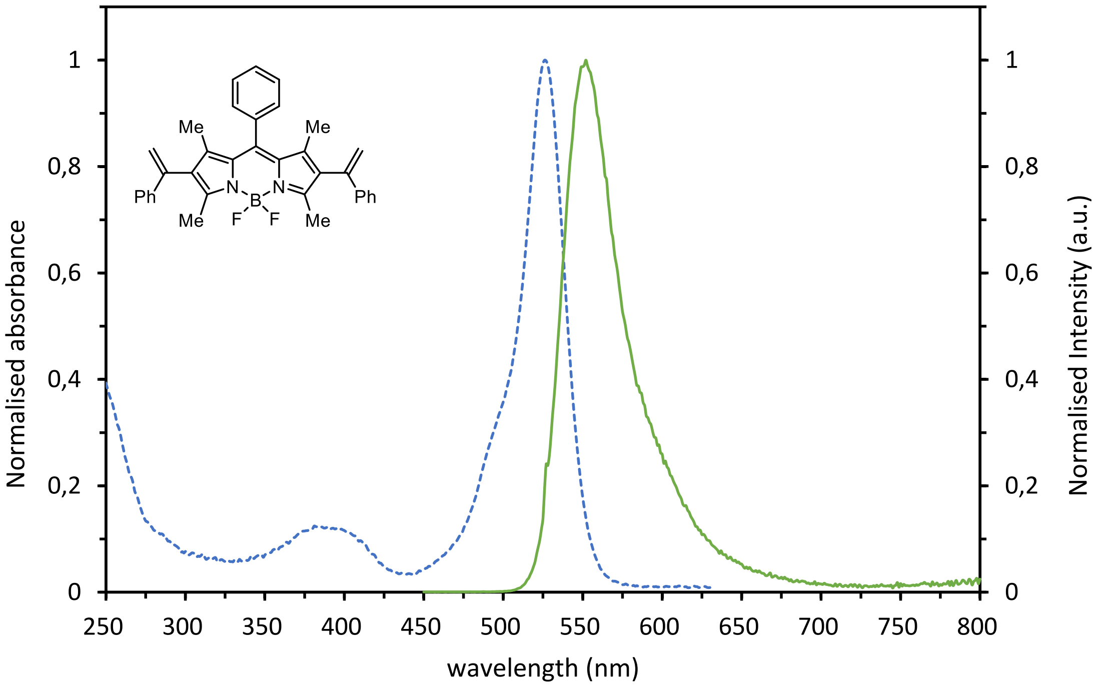

Figure S5. Absorption and Emission spectra of BODIPY **2a** registered in $CHCl_3$ ([**2a**]= $7.5\cdot10^{-7}$ M). λ_{max}(abs)= 526 nm; λ_{max}(em)= 552 nm.

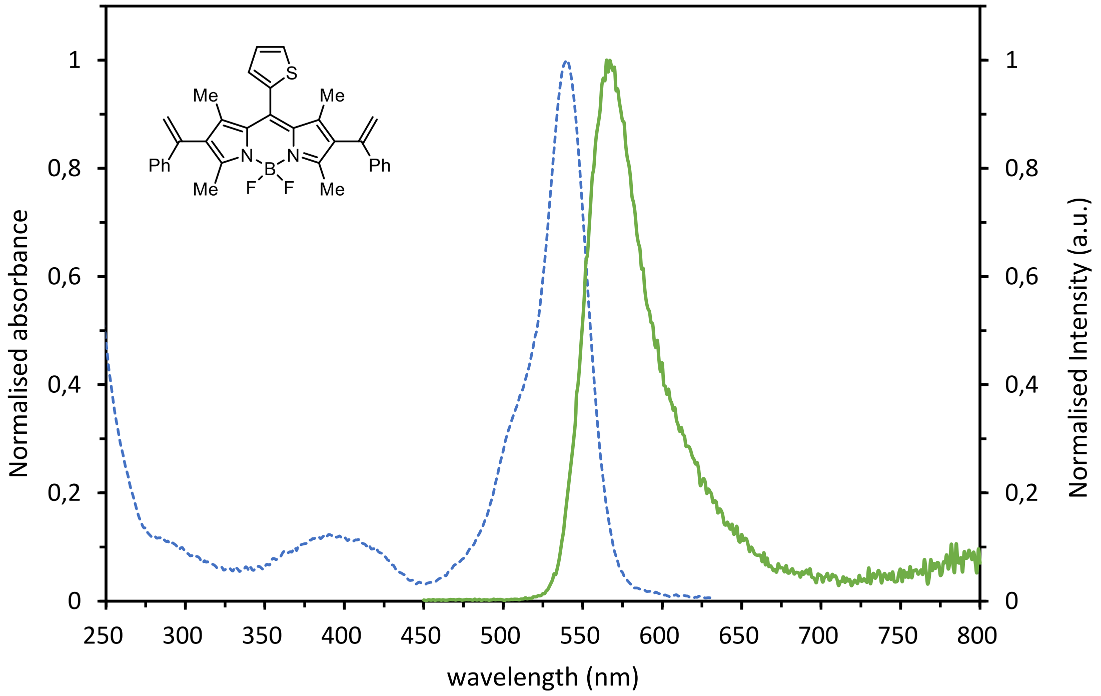

Figure S6. Absorption and Emission spectra of BODIPY **2b** registered in $CHCl_3$ ([**2b**]= $7.5\cdot10^{-7}$ M). λ_{max}(abs)= 540 nm; λ_{max}(em)= 565 nm.

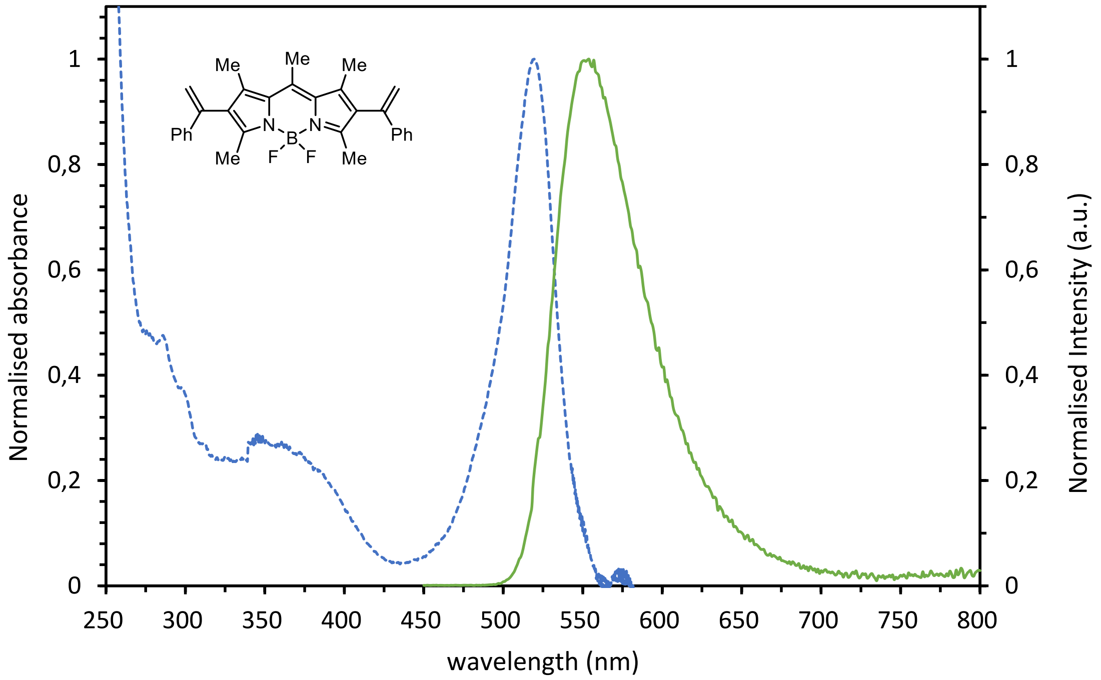

Figure S7. Absorption and Emission spectra of BODIPY **2c** registered in $CHCl_3$ ([**2c**]= $1.0 \cdot 10^{-6}$ M). λ_{max}(abs)= 520 nm; λ_{max}(em)= 554 nm.

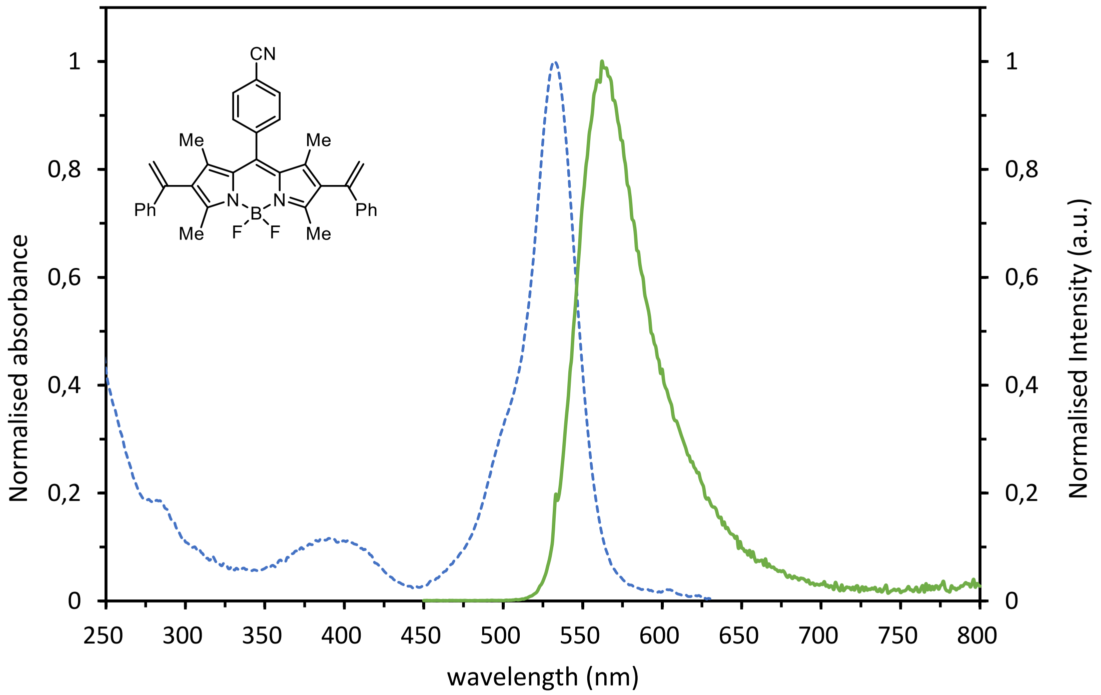

Figure S8. Absorption and Emission spectra of BODIPY **2d** registered in $CHCl_3$ ([**2d**]= $7.5 \cdot 10^{-7}$ M). λ_{max}(abs)= 532 nm; λ_{max}(em)= 562 nm.

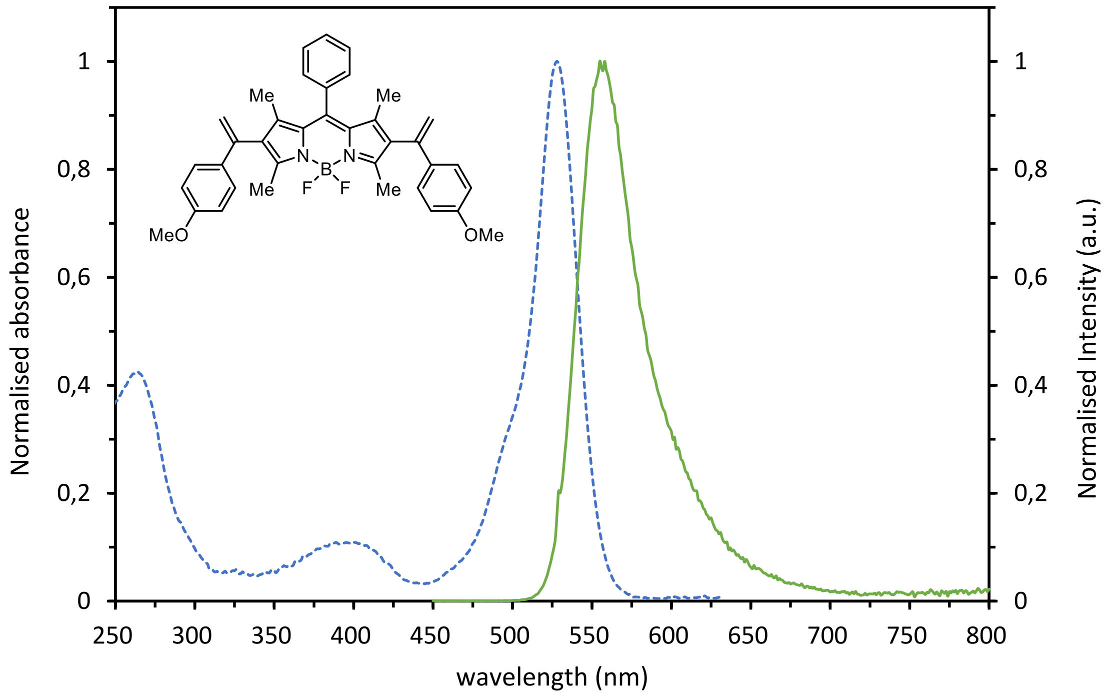

Figure S9. Absorption and Emission spectra of BODIPY **3a** registered in $CHCl_3$ ([**3a**]= $7.5 \cdot 10^{-7}$ M). λ_{max}(abs)= 528 nm; λ_{max}(em)= 558 nm.

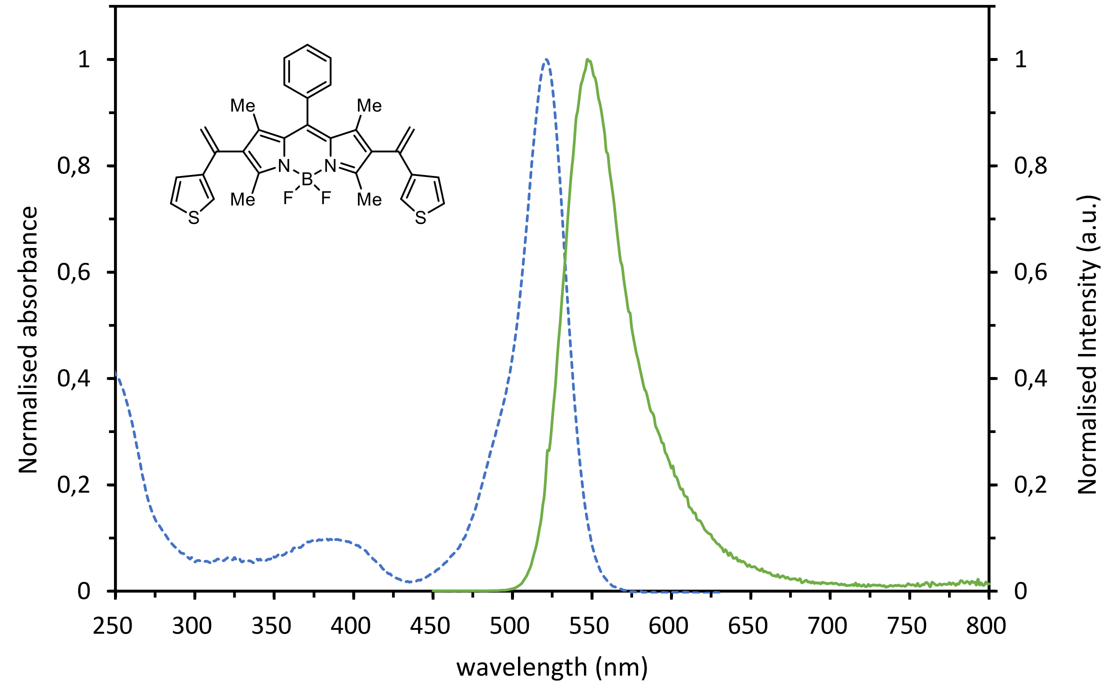

Figure S10. Absorption and Emission spectra of BODIPY **4a** registered in $CHCl_3$ ([**5a**]= $7.5 \cdot 10^{-7}$ M). λ_{max}(abs)= 521 nm; λ_{max}(em)= 547 nm.

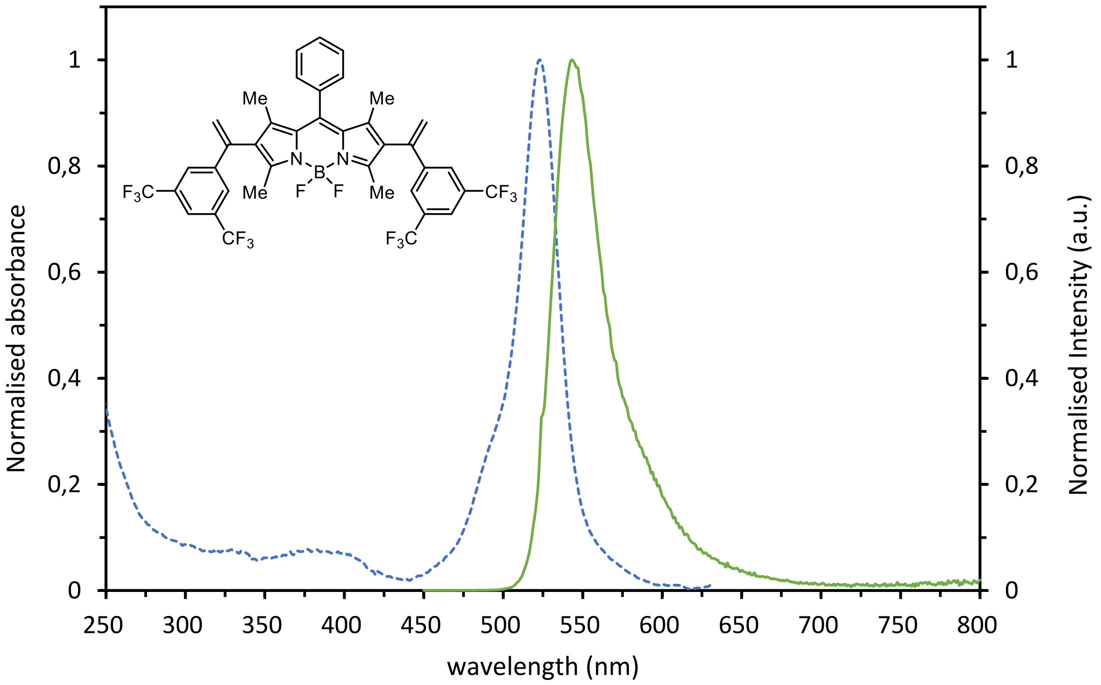

Figure S11. Absorption and Emission spectra of BODIPY **5a** registered in $CHCl_3$ ([**4a**]= $7.5\cdot10^{-7}$ M). λ_{max}(abs)= 523 nm; λ_{max}(em)= 543 nm.

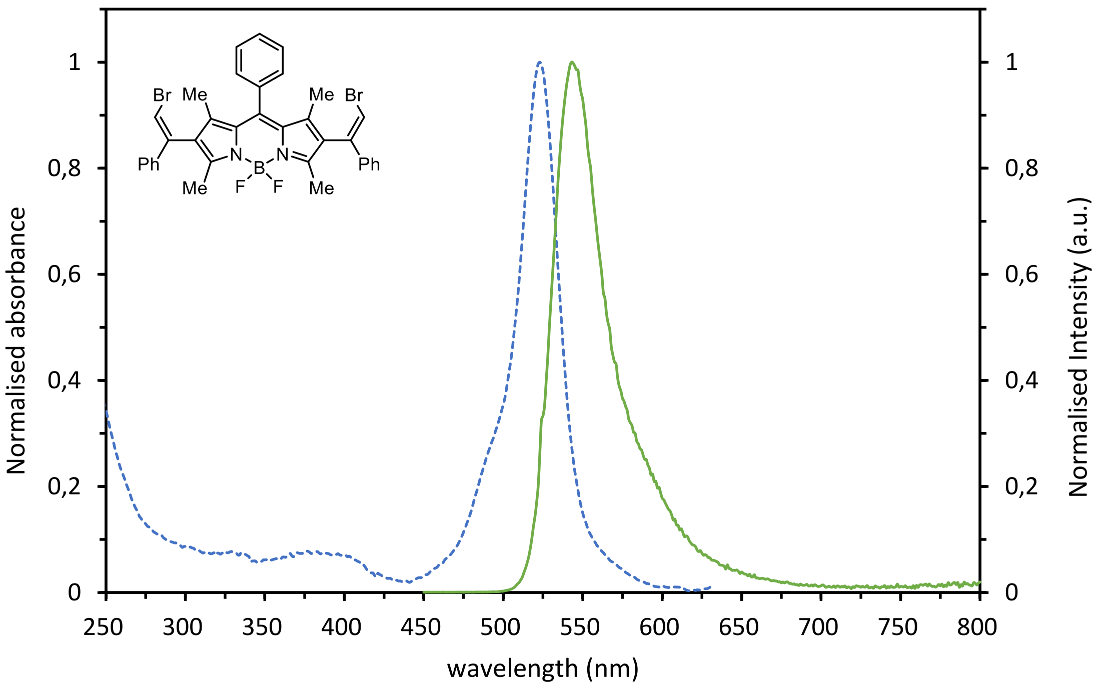

Figure S12. Absorption and Emission spectra of BODIPY **6a** registered in $CHCl_3$ ([**6a**]= $7.5\cdot10^{-7}$ M). λ_{max}(abs)= 523 nm; λ_{max}(em)= 538 nm.

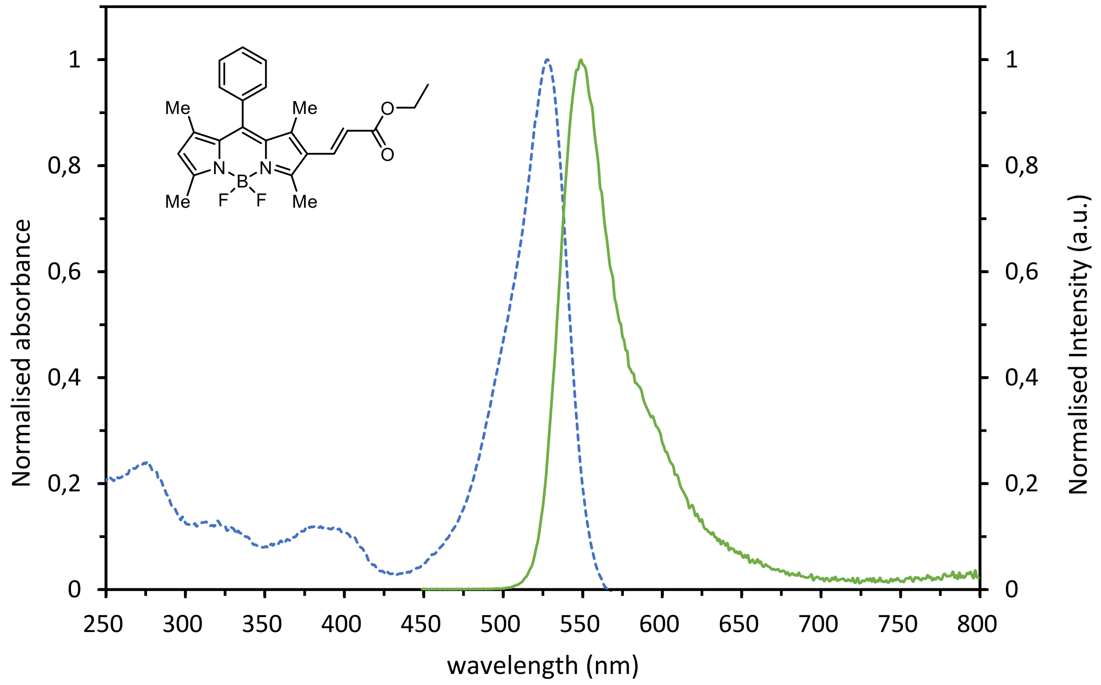

Figure S13. Absorption and Emission spectra of BODIPY **7a** registered in $CHCl_3$ ([**7a**]= $7.5\cdot10^{-7}$ M). λ_{max}(abs)= 528 nm; λ_{max}(em)= 549 nm.

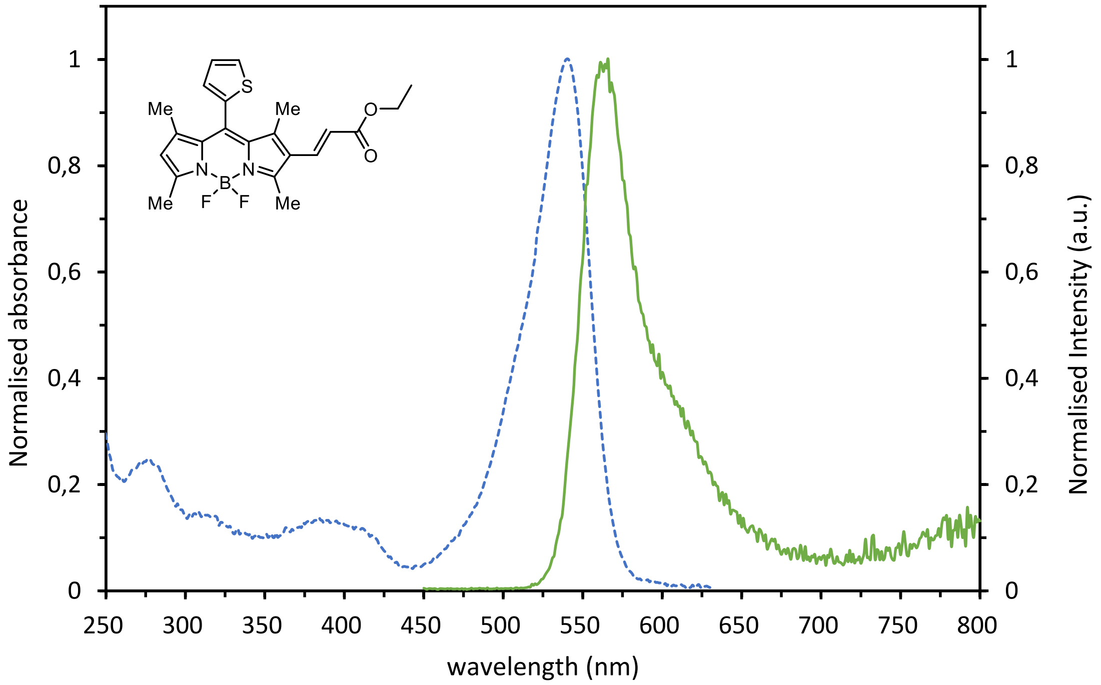

Figure S14. Absorption and Emission spectra of BODIPY **7b** registered in $CHCl_3$ ([**7b**]= $7.5\cdot10^{-7}$ M). λ_{max}(abs)= 541 nm; λ_{max}(em)= 566 nm.

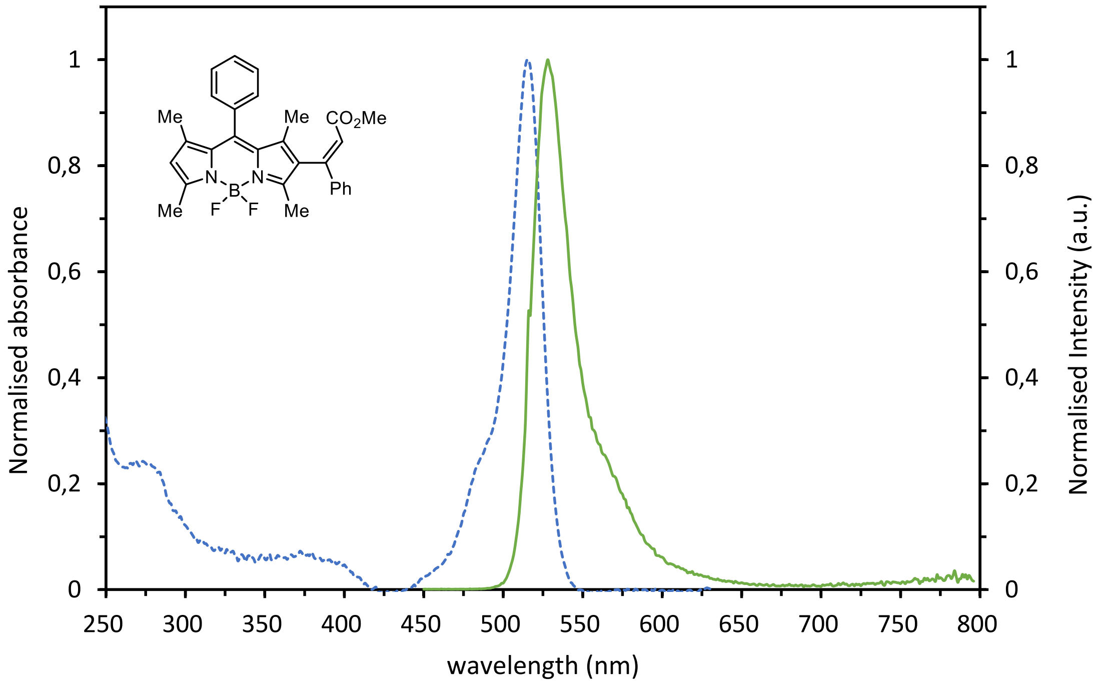

Figure S15. Absorption and Emission spectra of BODIPY **8a** registered in $CHCl_3$ ([**8a**]= $7.5 \cdot 10^{-7}$ M). λ_{max}(abs)= 515 nm; λ_{max}(em)= 528 nm.

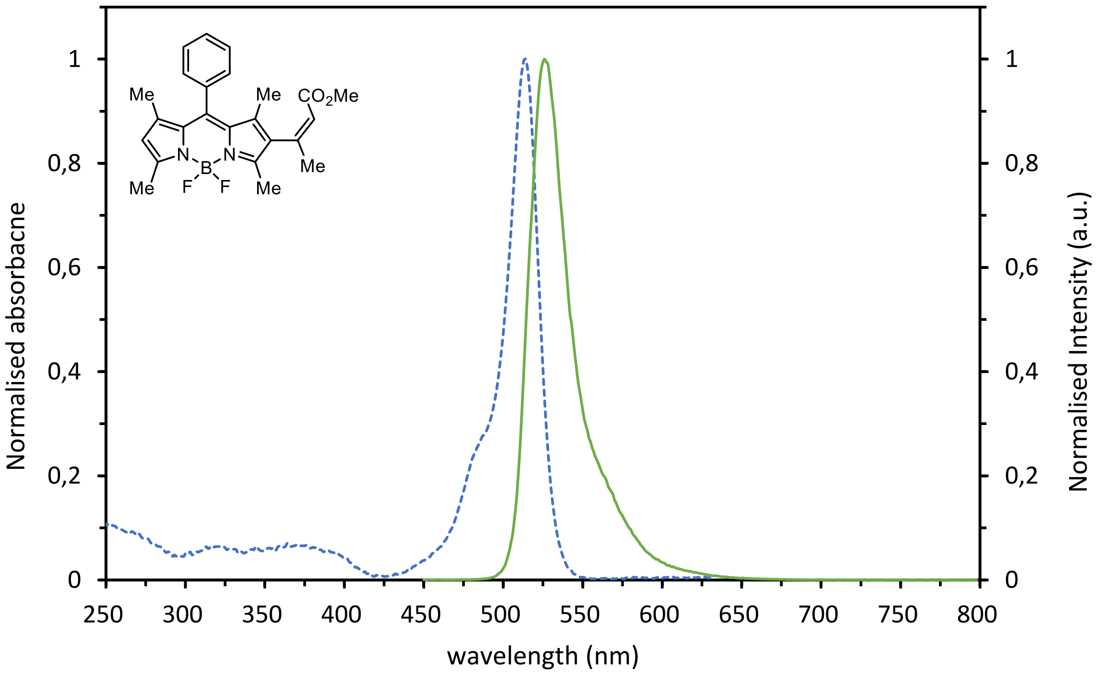

Figure S16. Absorption and Emission spectra of BODIPY **9a** registered in $CHCl_3$ ([**9a**]= $7.5 \cdot 10^{-7}$ M). λ_{max}(abs)= 514 nm; λ_{max}(em)= 526 nm.

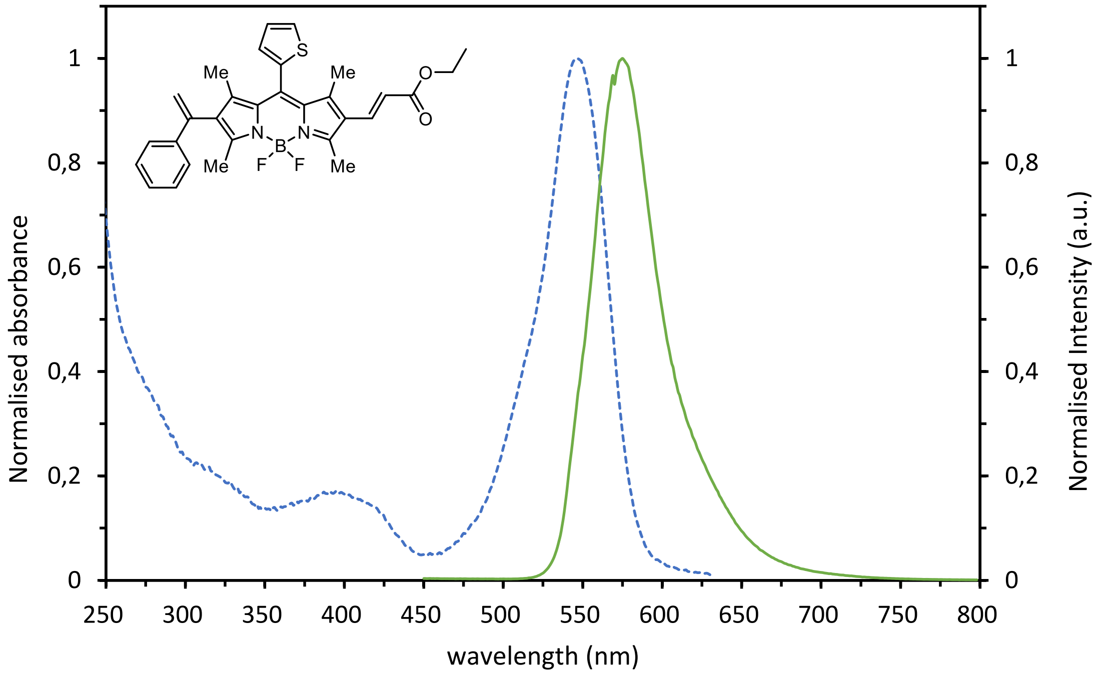

Figure S17. Absorption and Emission spectra of BODIPY **8b** registered in $CHCl_3$ ([**8b**]= $7.5\cdot10^{-7}$ M). λ_{max}(abs)= 546 nm; λ_{max}(em)= 575 nm.

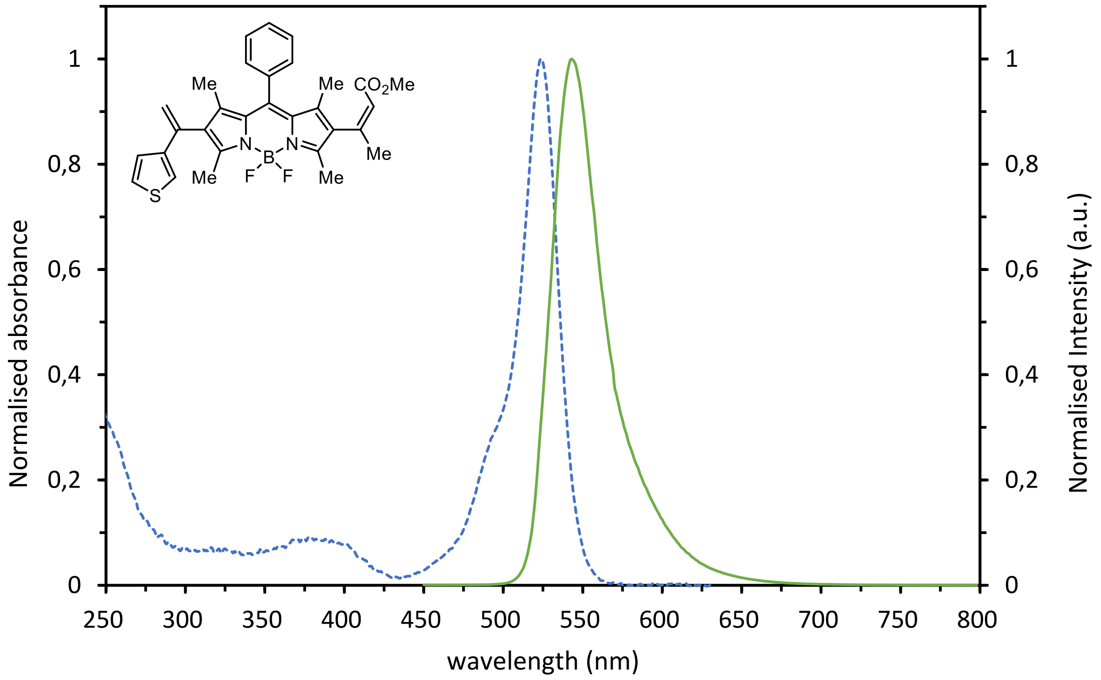

Figure S18. Absorption and Emission spectra of BODIPY **10a** registered in $CHCl_3$ ([**10a**]= $7.5\cdot10^{-7}$ M). λ_{max}(abs)= 524 nm; λ_{max}(em)= 544 nm.

300 MHz ^{1}H-NMR Spectrum of compound **2a** ($CDCl_3$, 300 K)

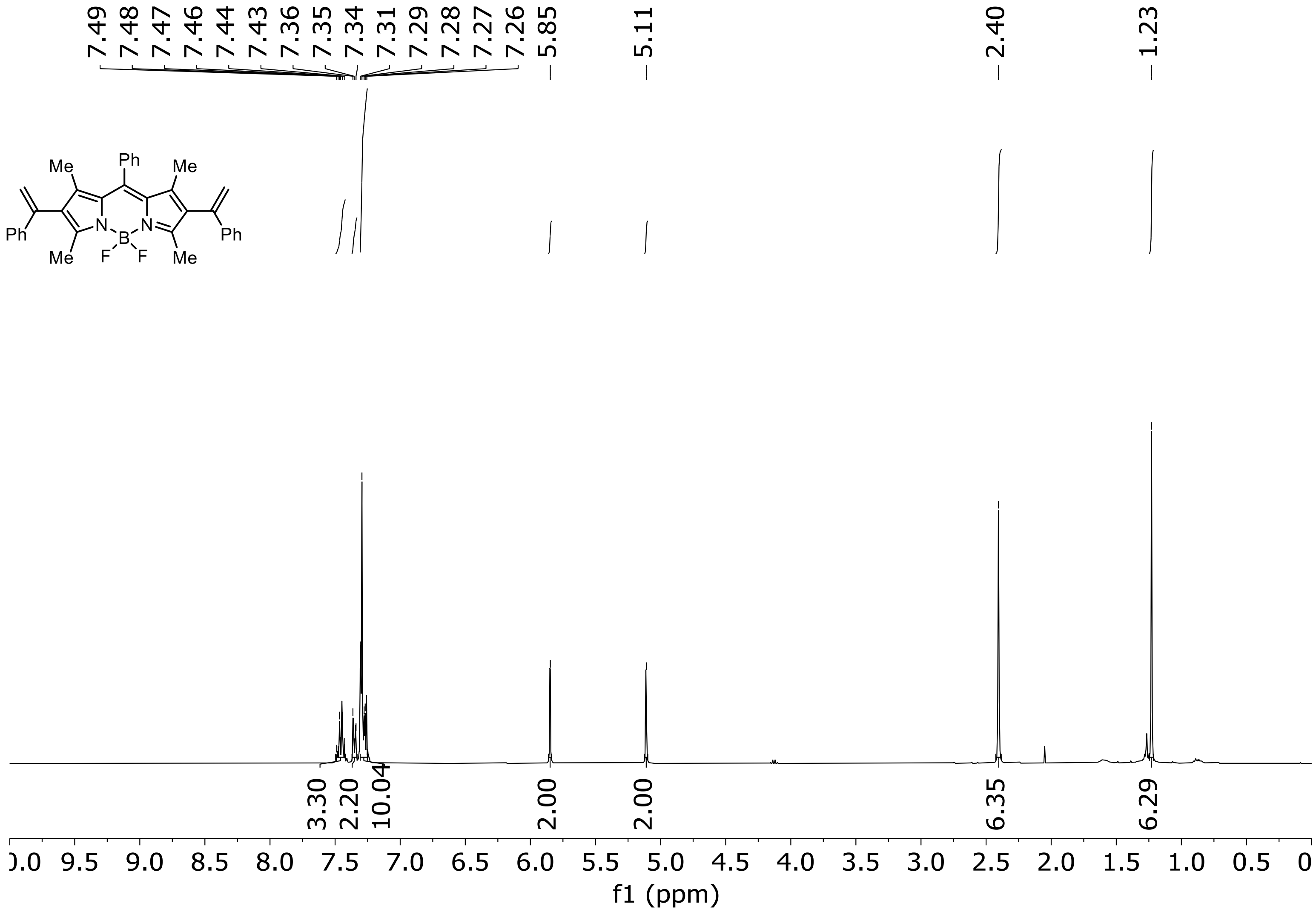

75 MHz $^{13}C\{^{1}H\}$-NMR Spectrum of compound **2a** ($CDCl_3$, 300 K)

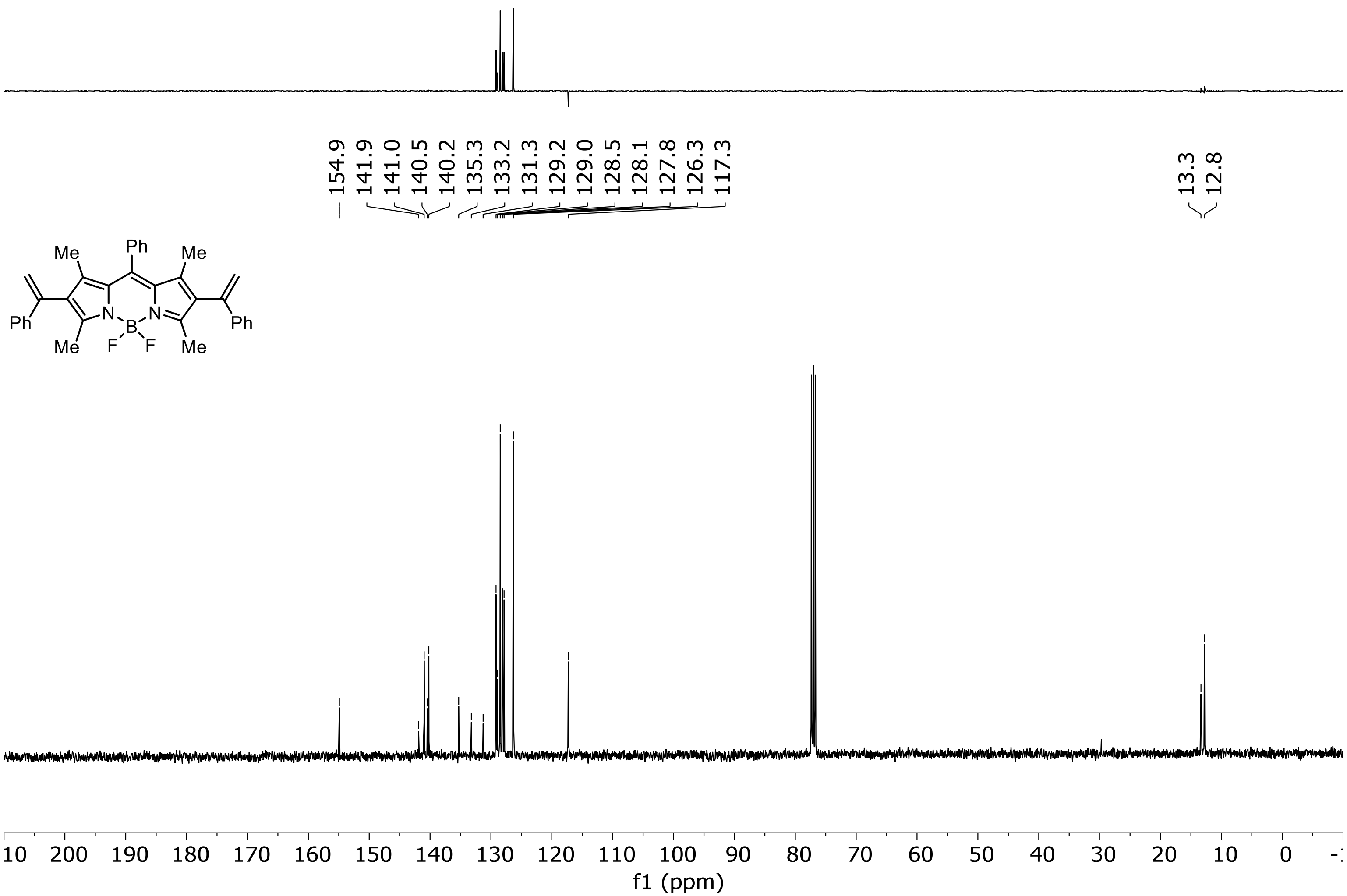

282 MHz ^{19}F-NMR Spectrum of compound **2a** ($CDCl_3$, 300 K)

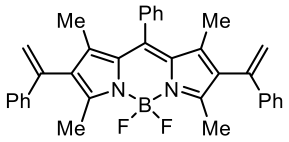

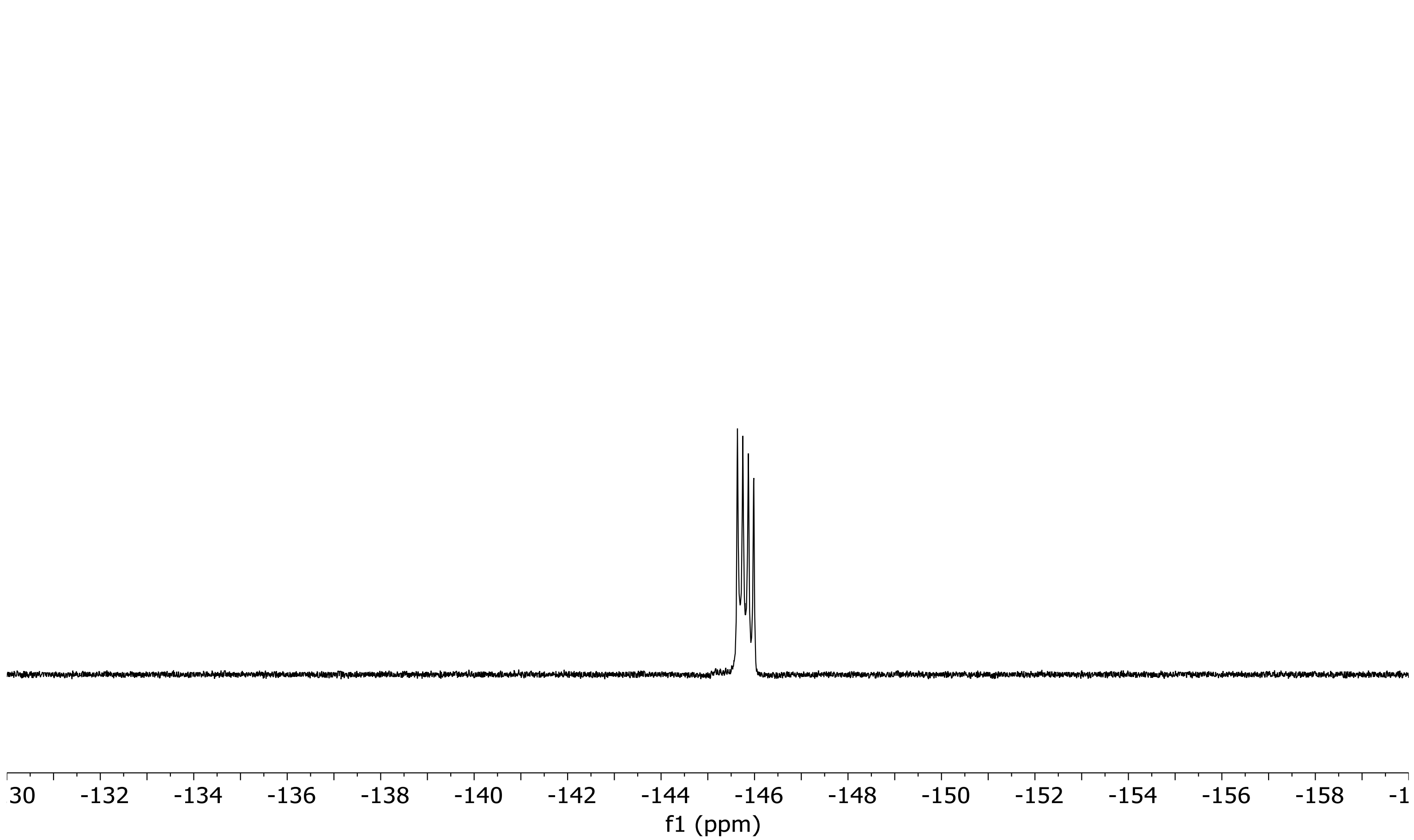

300 MHz ^{1}H-NMR Spectrum of compound **2b** ($CDCl_3$, 300 K)

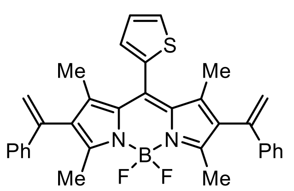

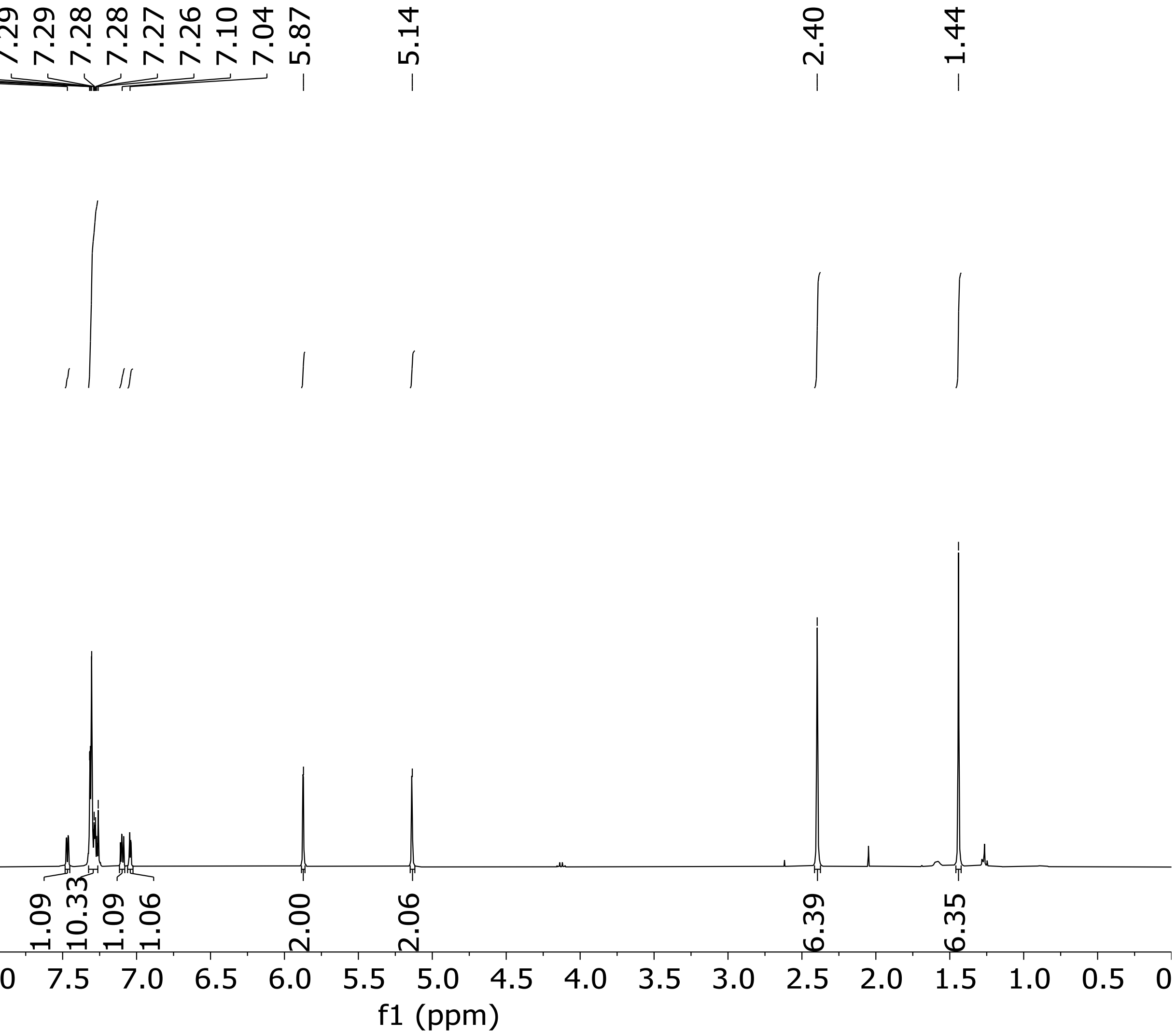

75 MHz $^{13}C\{^{1}H\}$-NMR Spectrum of compound **2b** ($CDCl_3$, 300 K)

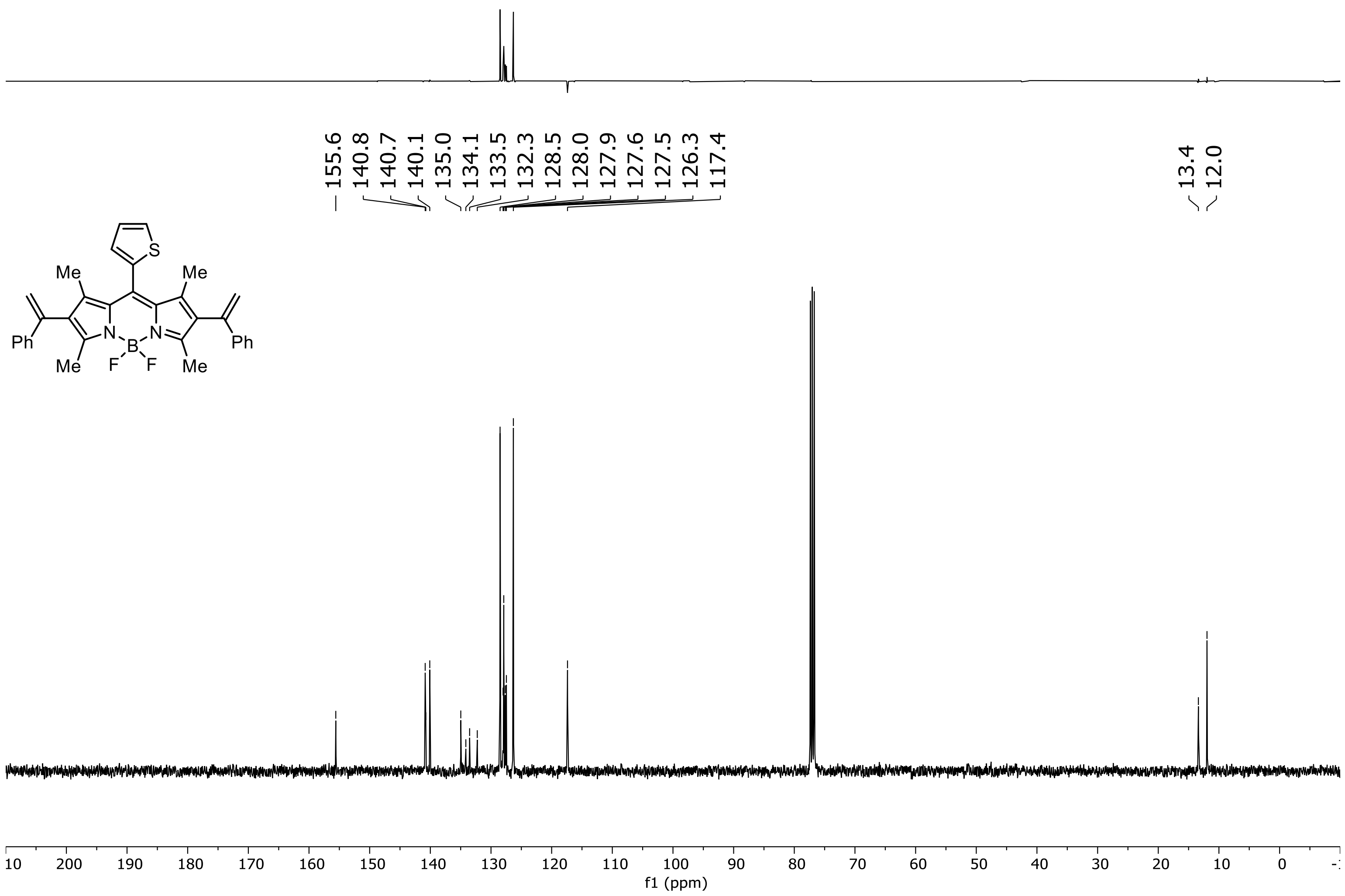

282 MHz ^{19}F-NMR Spectrum of compound **2b** ($CDCl_3$, 300 K)

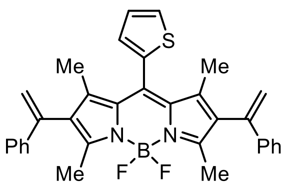

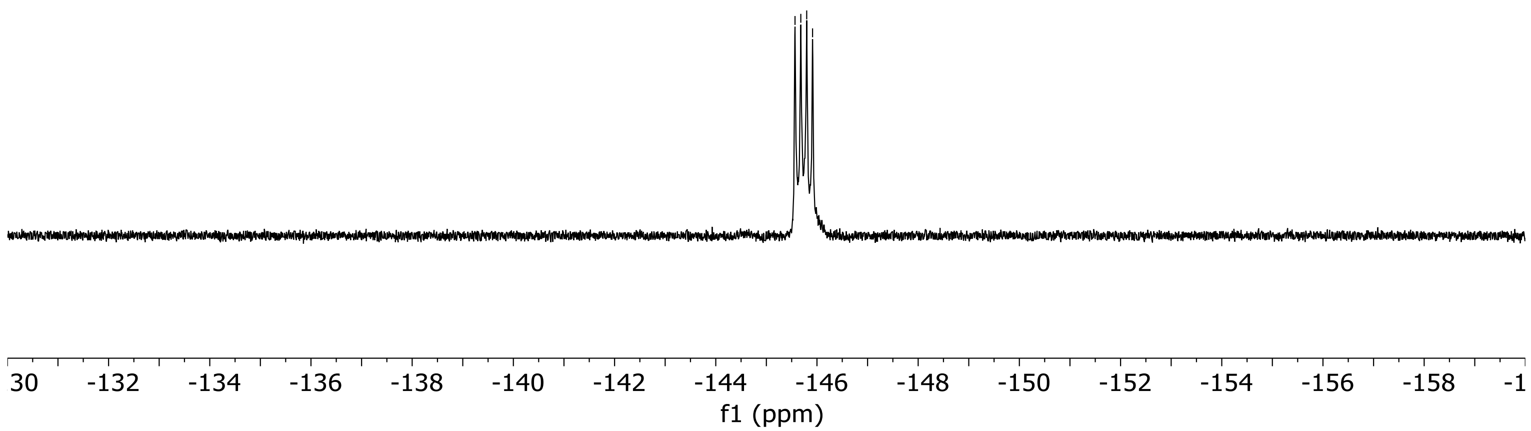

300 MHz ^{1}H-NMR Spectrum of compound **2c** ($CDCl_3$, 300 K)

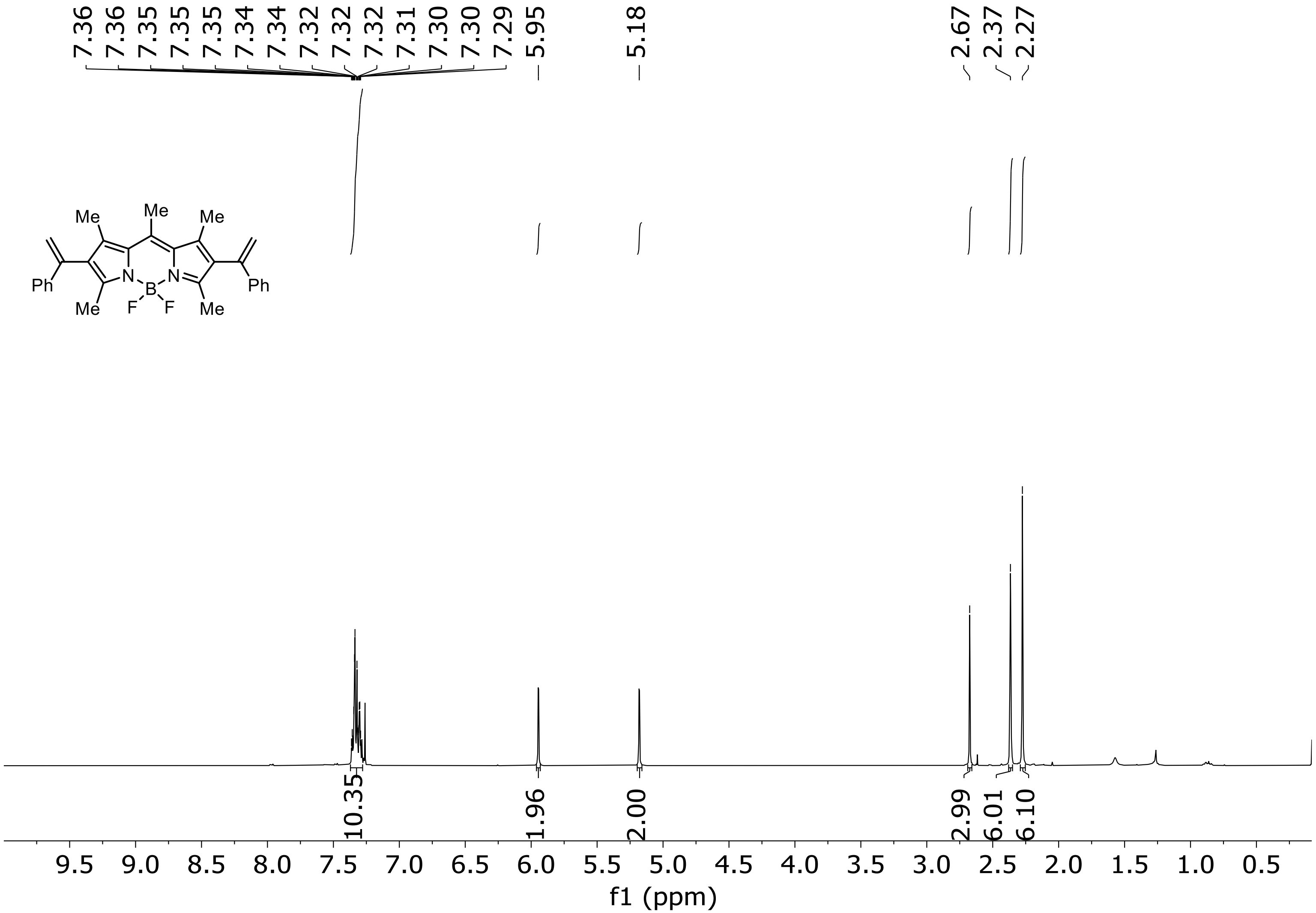

75 MHz $^{13}C\{^{1}H\}$-NMR Spectrum of compound **2c** ($CDCl_3$, 300 K)

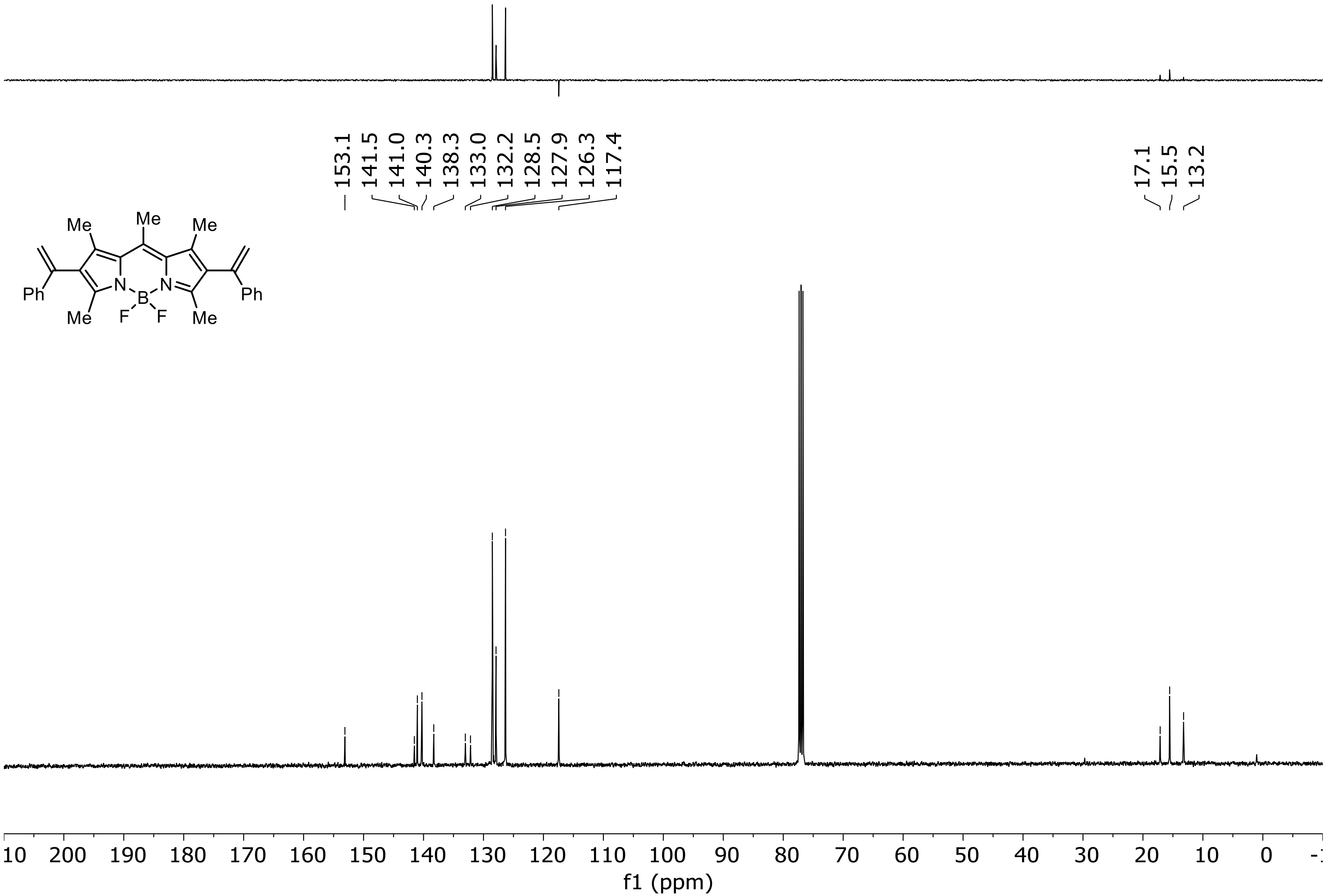

282 MHz ^{19}F-NMR Spectrum of compound **2c** ($CDCl_3$, 300 K)

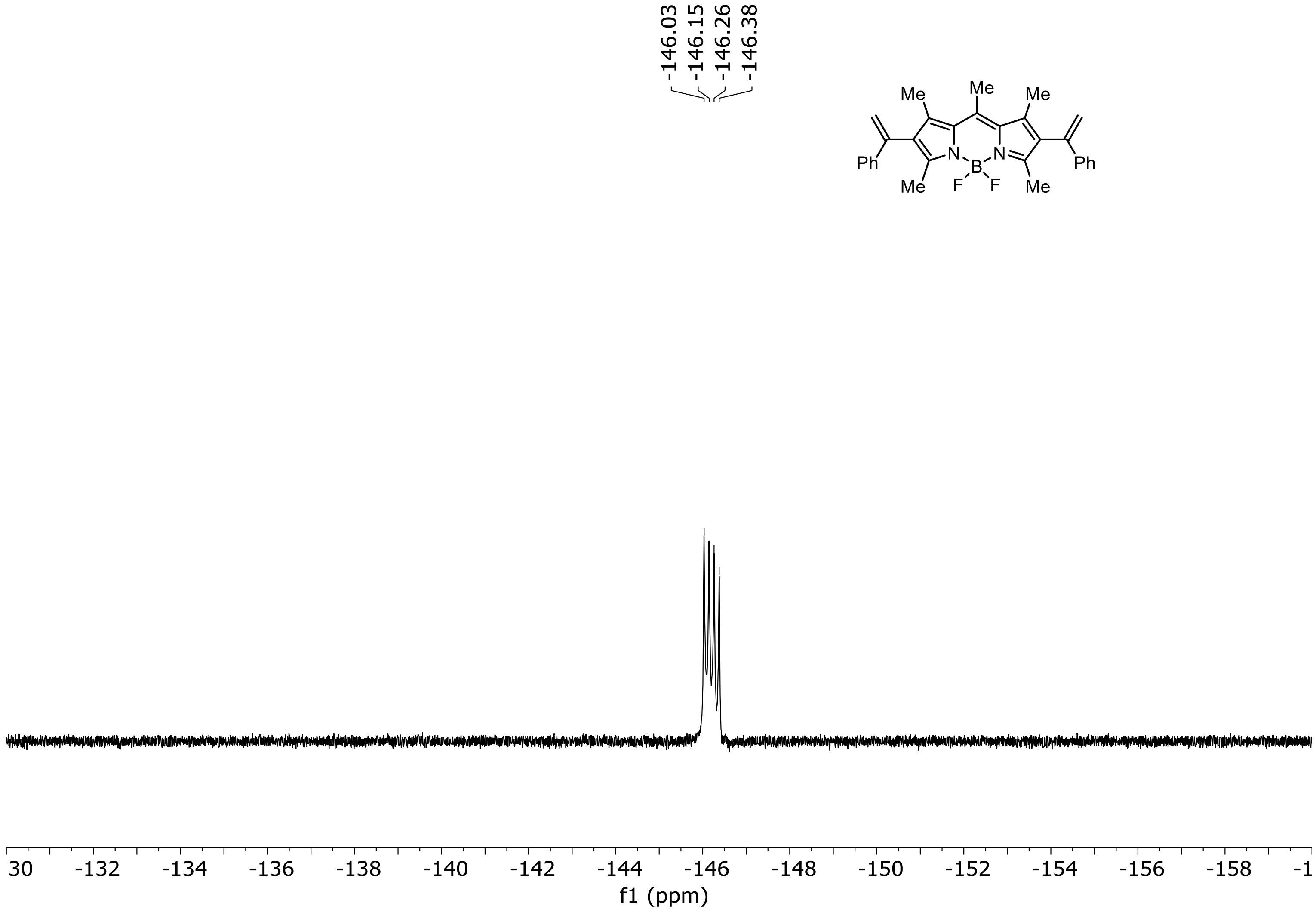

300 MHz ¹H-NMR Spectrum of compound **2d** ($CDCl_3$, 300 K)

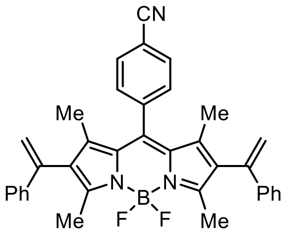

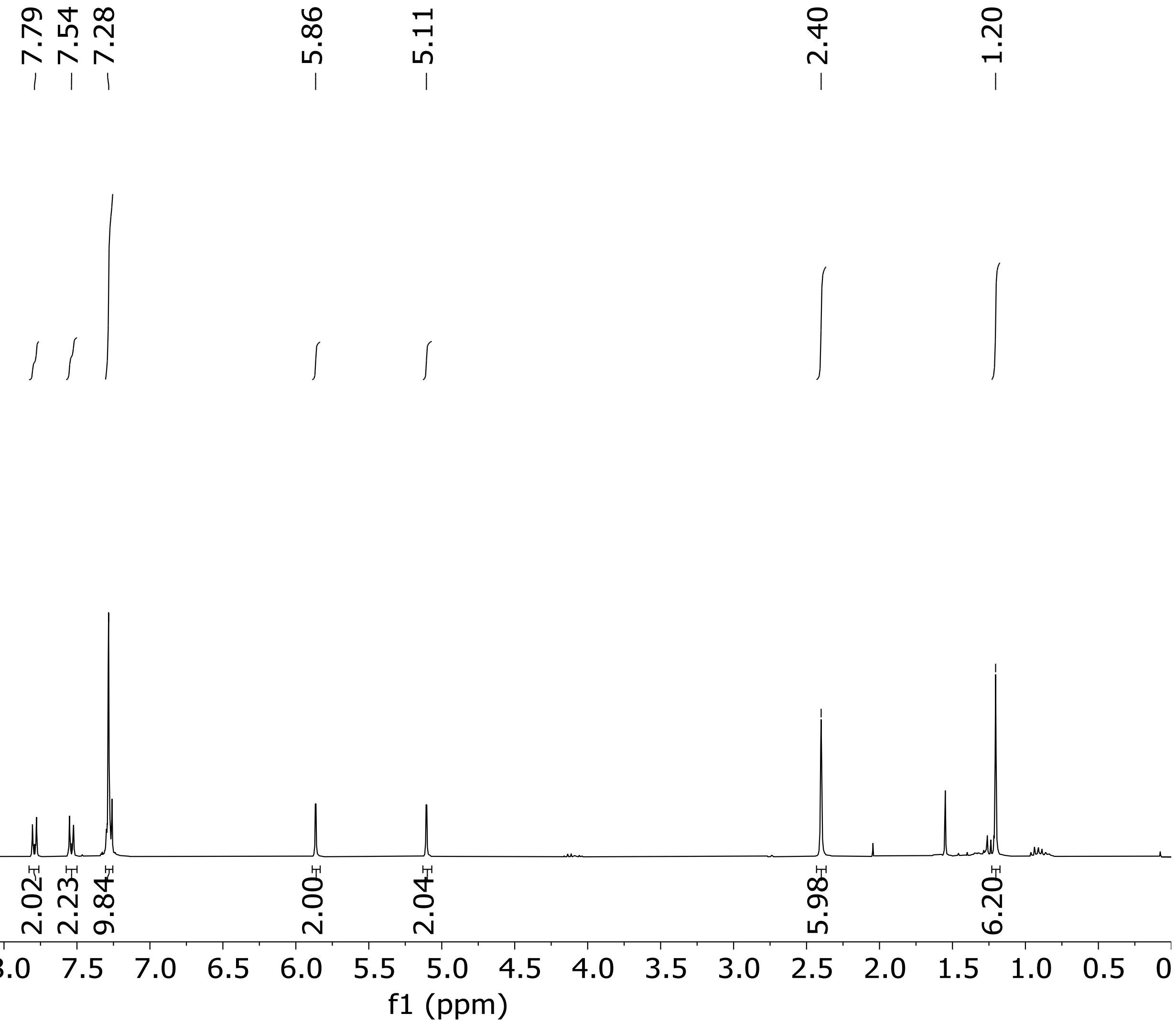

75 MHz $^{13}C\{^{1}H\}$-NMR Spectrum of compound **2d** ($CDCl_3$, 300 K)

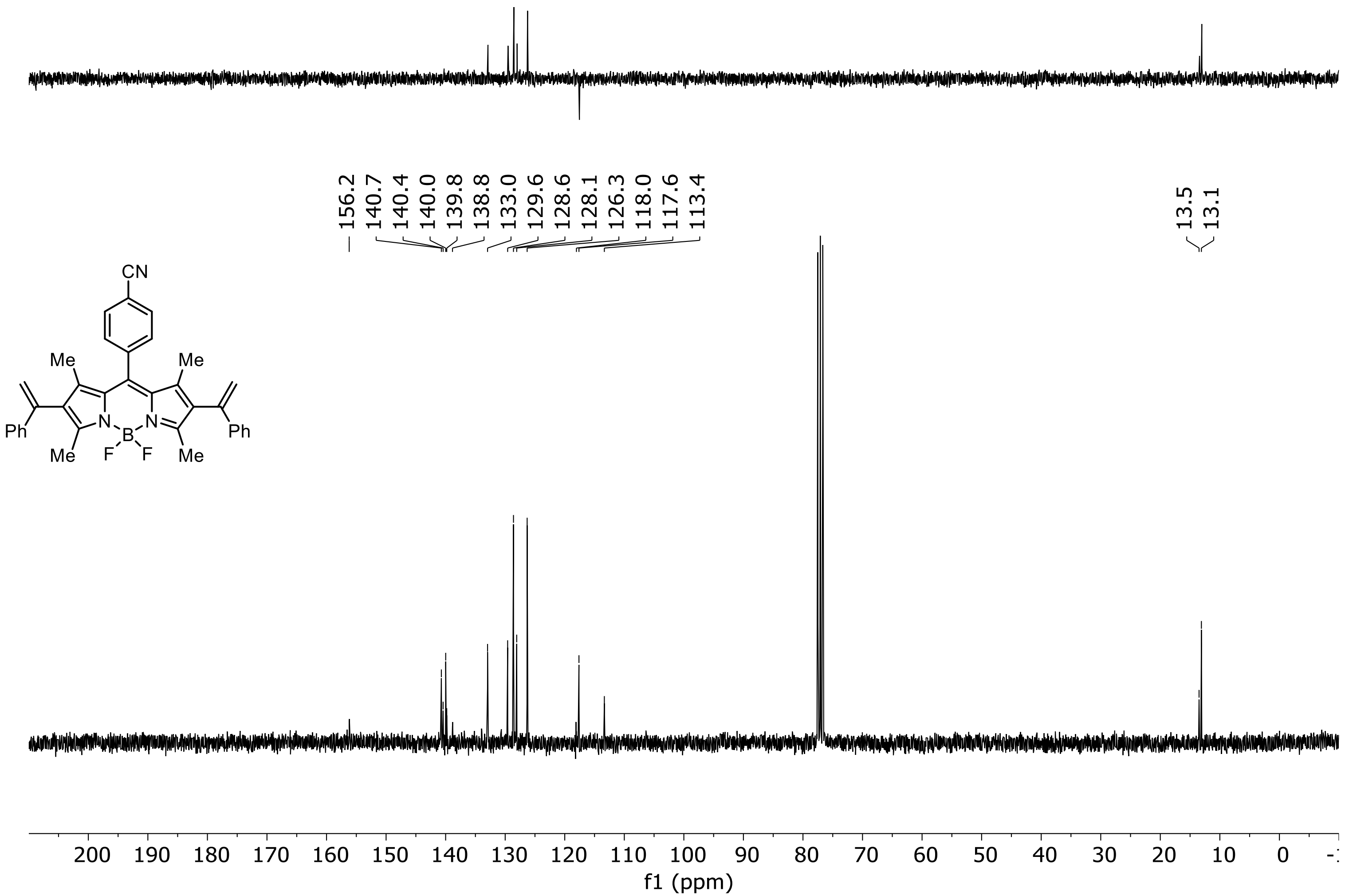

282 MHz ^{19}F-NMR Spectrum of compound **2d** ($CDCl_3$, 300 K)

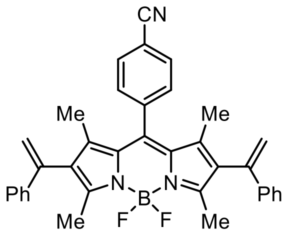

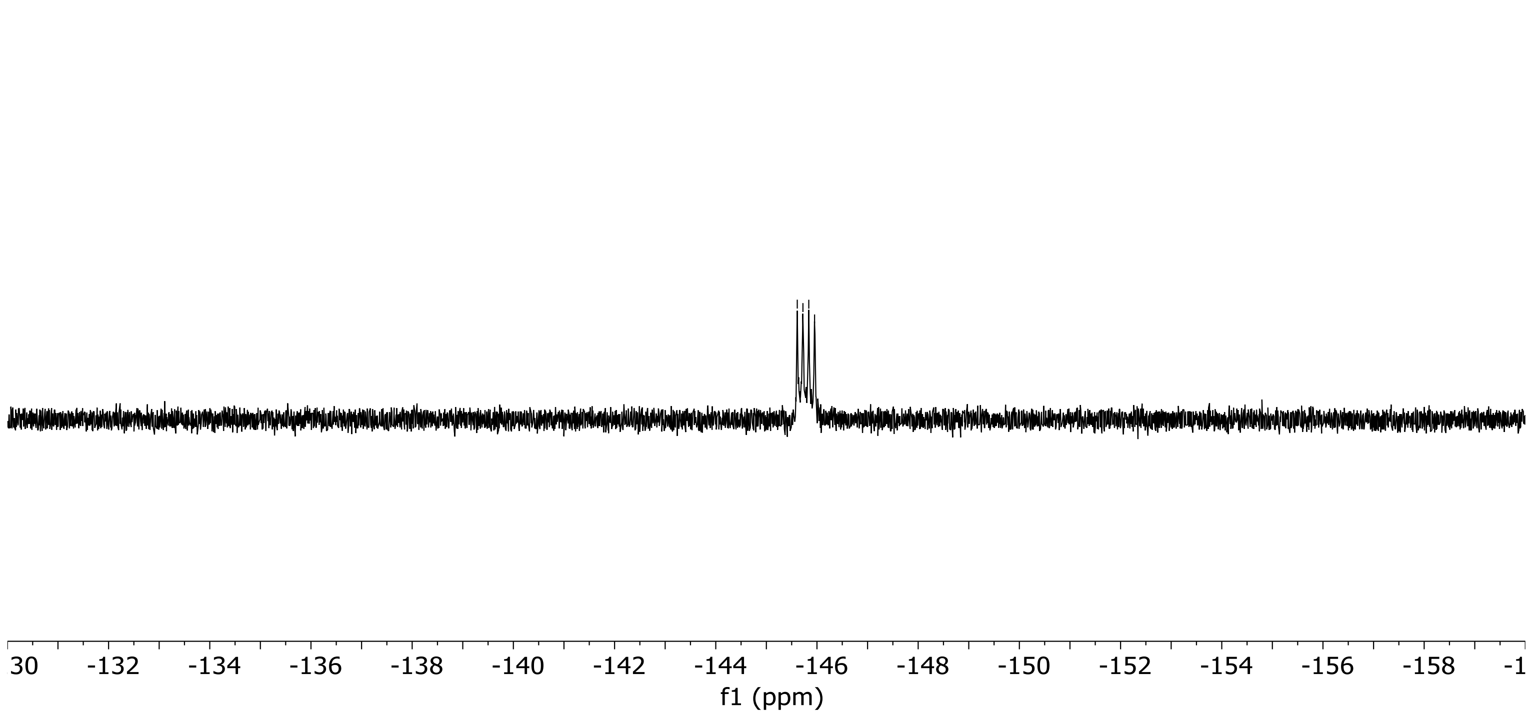

300 MHz ^{1}H-NMR Spectrum of compound **11** ($CDCl_3$, 300 K)

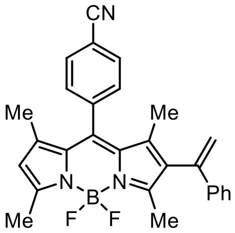

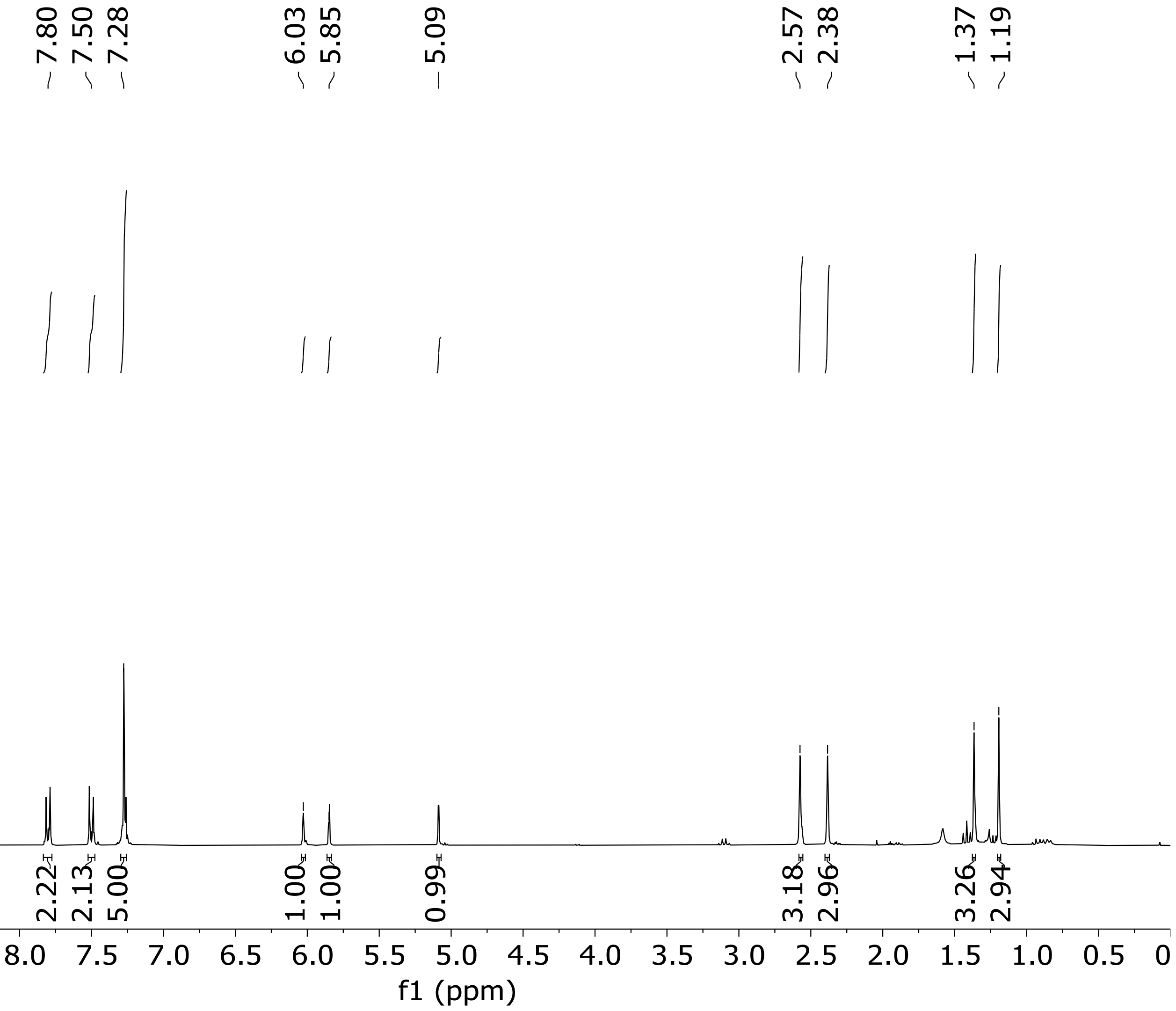

75 MHz $^{13}C\{^{1}H\}$-NMR Spectrum of compound **11** ($CDCl_3$, 300 K)

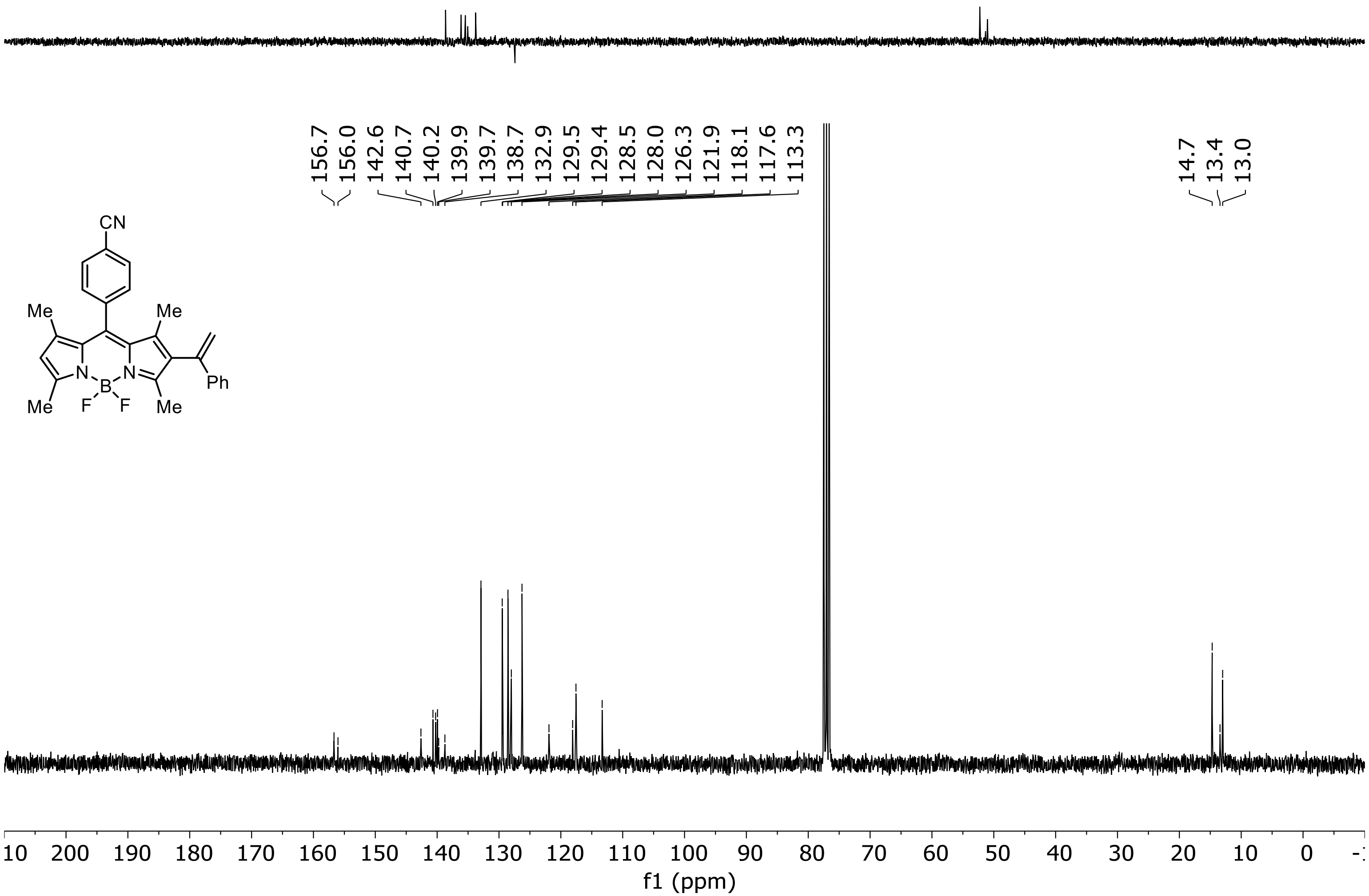

282 MHz ^{19}F-NMR Spectrum of compound **11** ($CDCl_3$, 300 K)

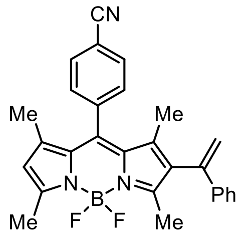

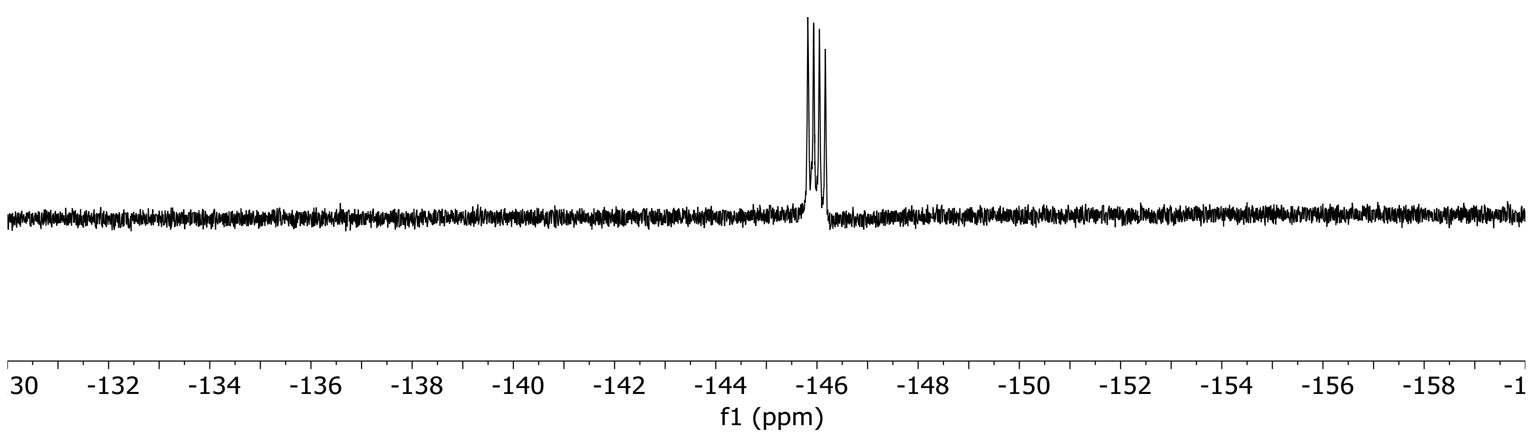

300 MHz ^{1}H-NMR Spectrum of compound **3a** ($CDCl_3$, 300 K)

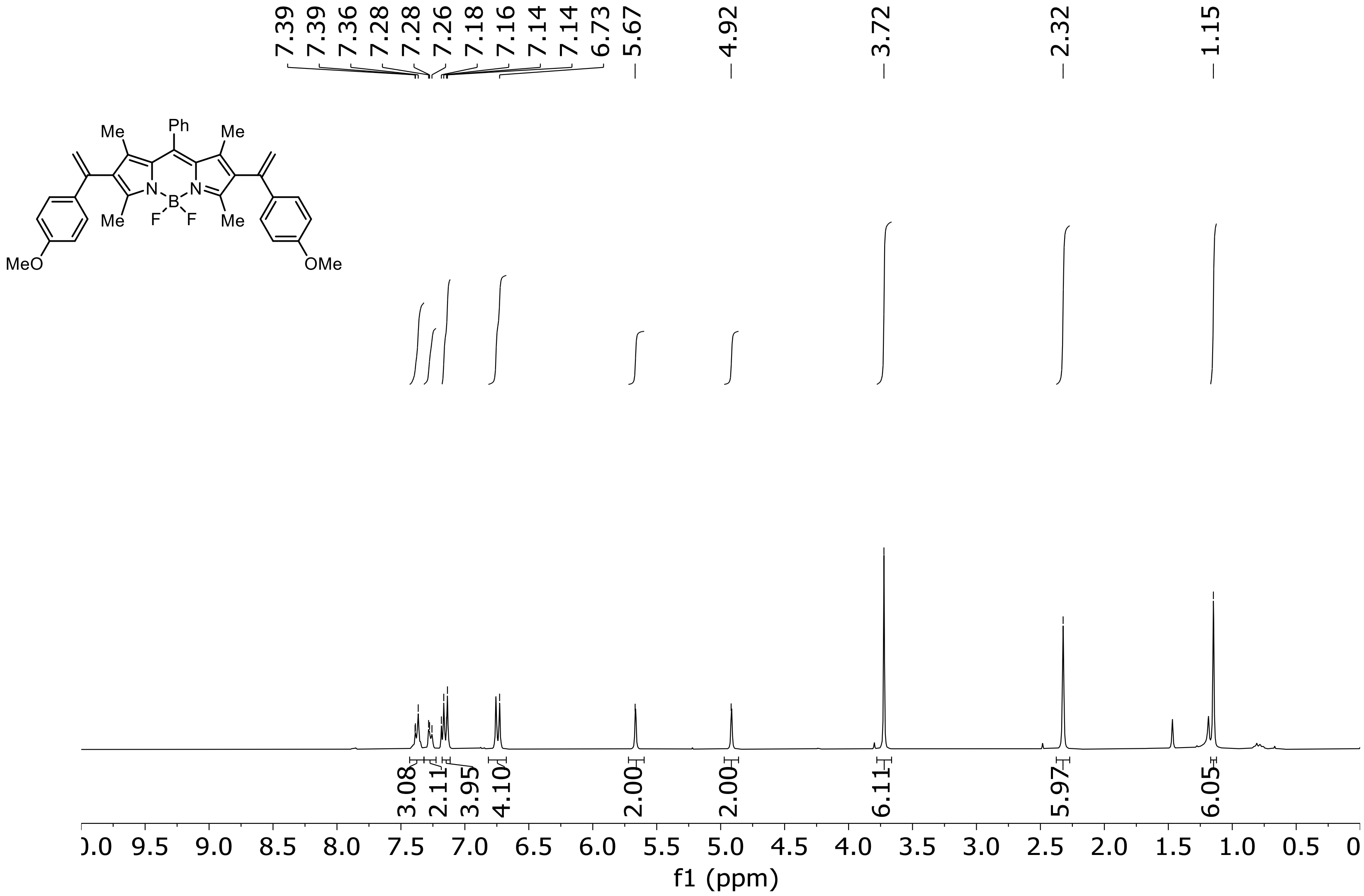

75 MHz $^{13}C\{^1H\}$-NMR Spectrum of compound **3a** ($CDCl_3$, 300 K)

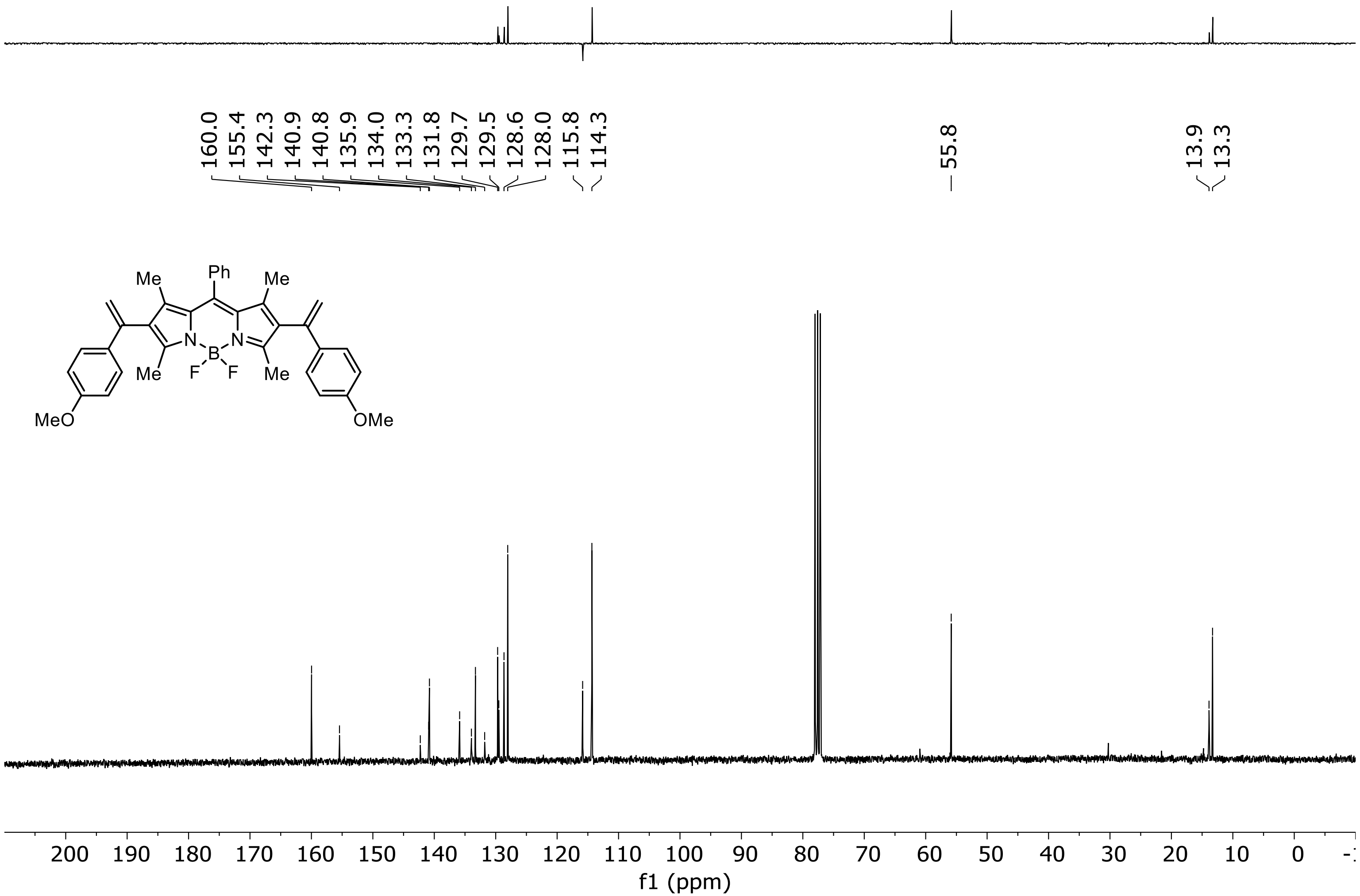

282 MHz ^{19}F-NMR Spectrum of compound **3a** ($CDCl_3$, 300 K)

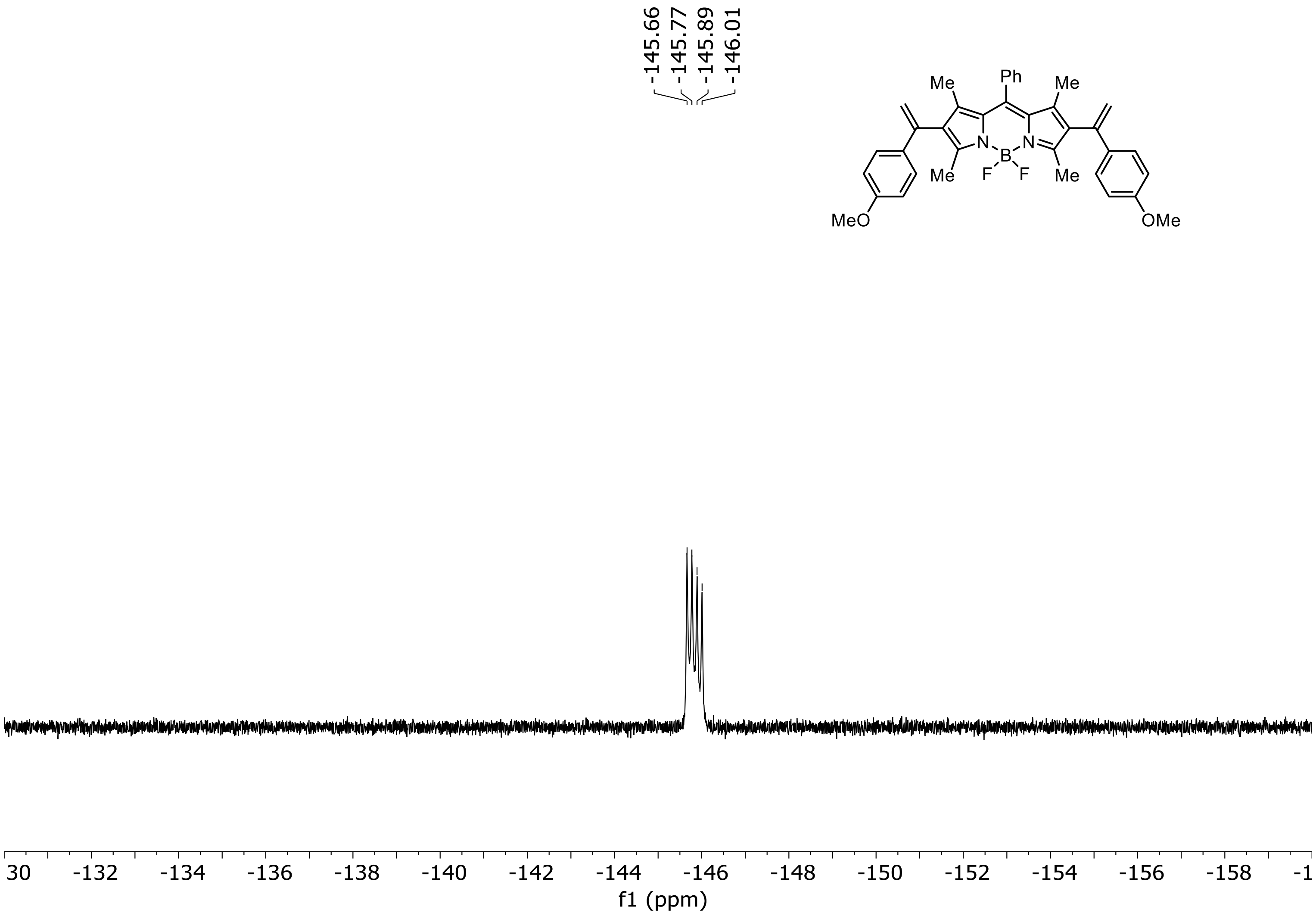

300 MHz ¹H-NMR Spectrum of compound **4a** ($CDCl_3$, 300 K)

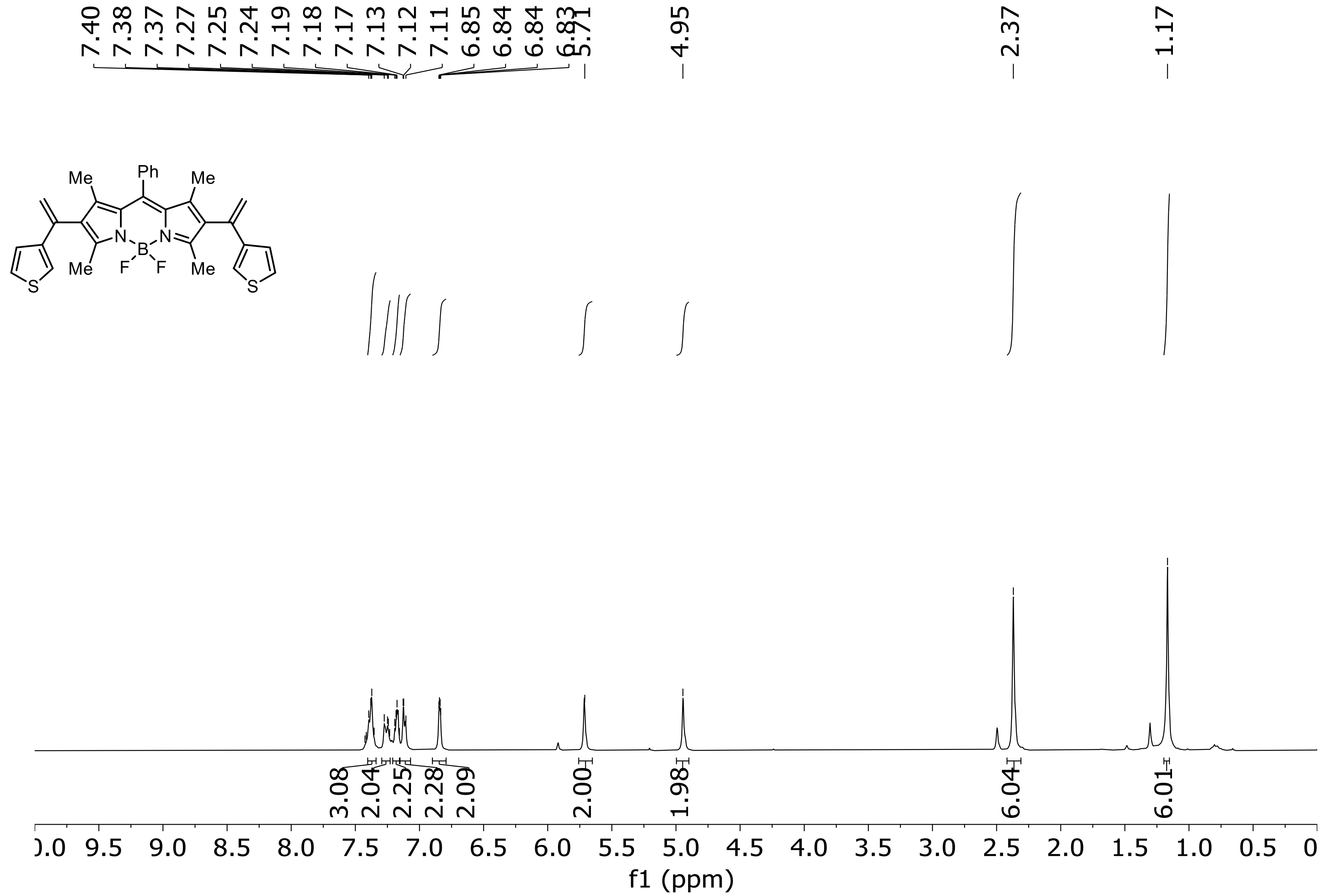

75 MHz $^{13}C\{^{1}H\}$-NMR Spectrum of compound **4a** ($CDCl_3$, 300 K)

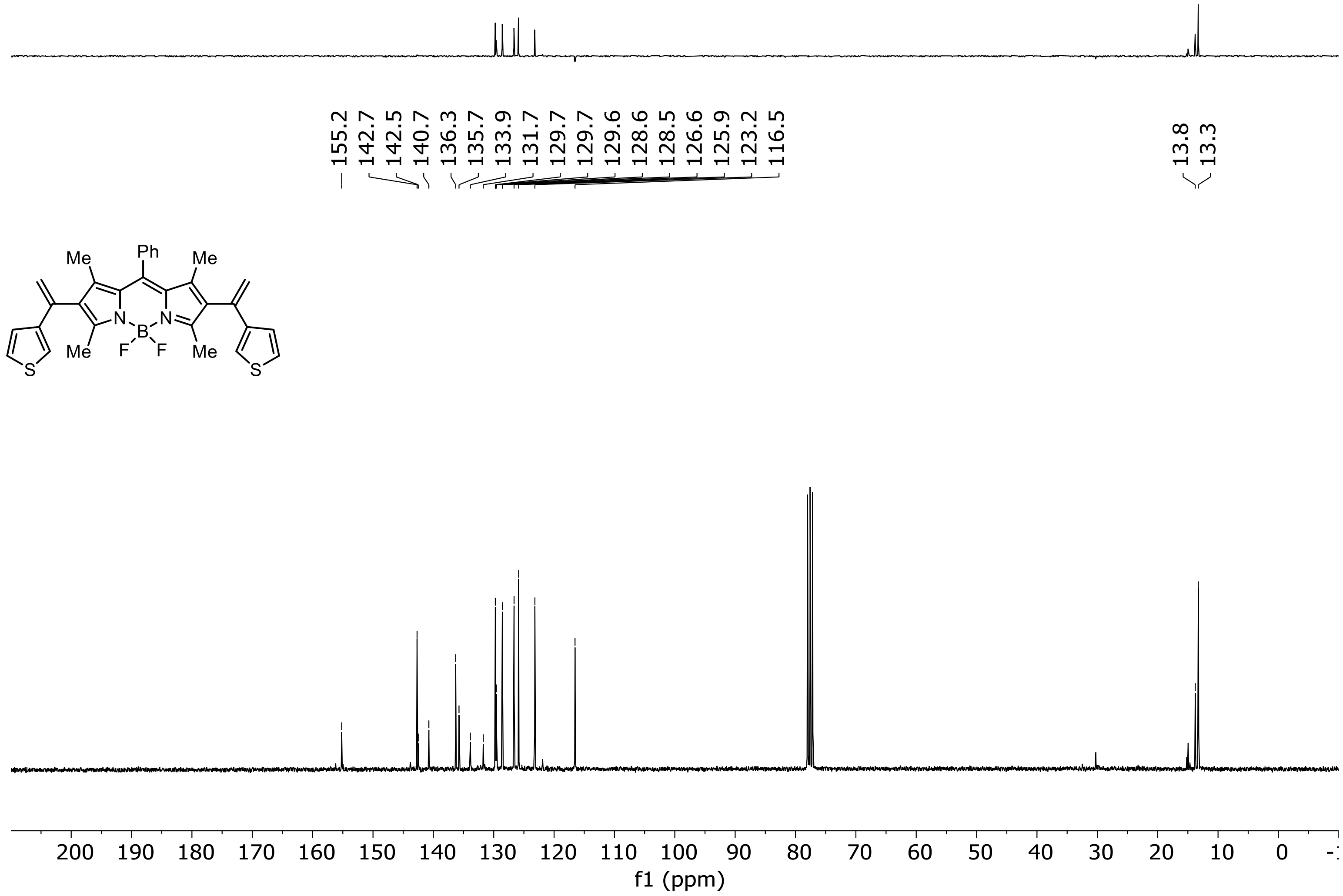

282 MHz ^{19}F-NMR Spectrum of compound **4a**($CDCl_3$, 300 K)

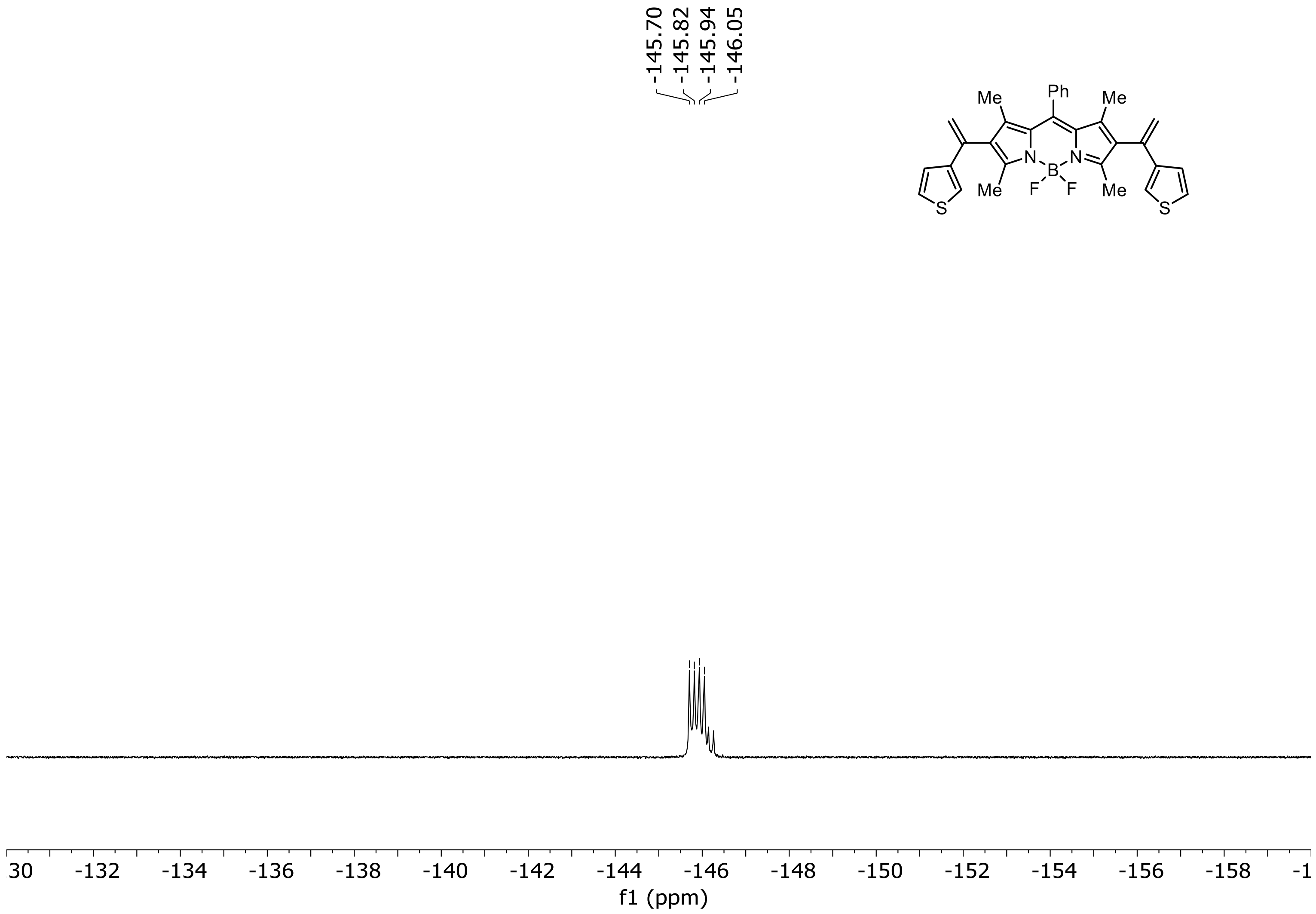

300 MHz ^{1}H-NMR Spectrum of compound **5a** ($CDCl_3$, 300 K)

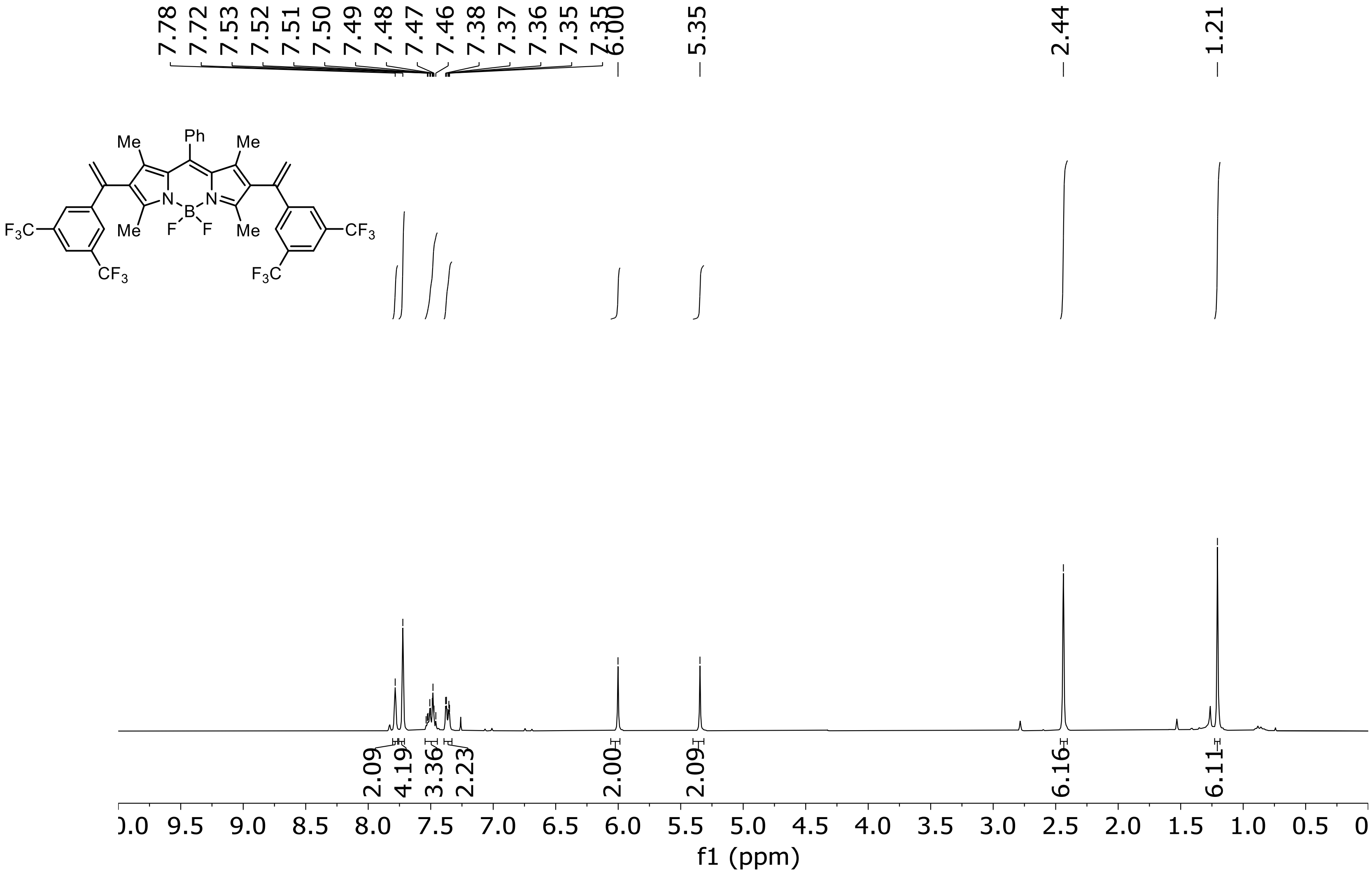

75 MHz $^{13}C\{^{1}H\}$-NMR Spectrum of compound **5a** ($CDCl_3$, 300 K)

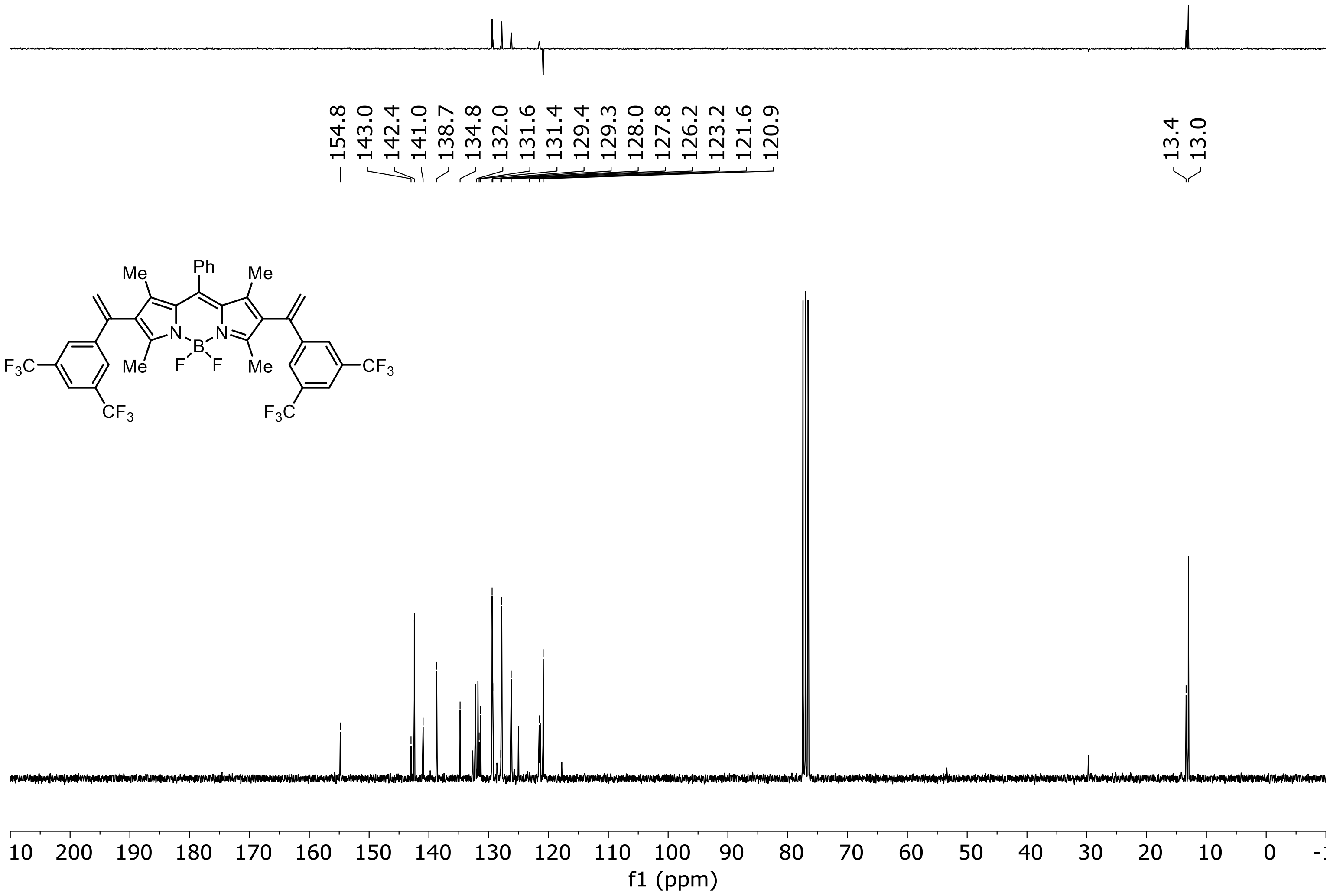

282 MHz ^{19}F-NMR Spectrum of compound **5a** ($CDCl_3$, 300 K)

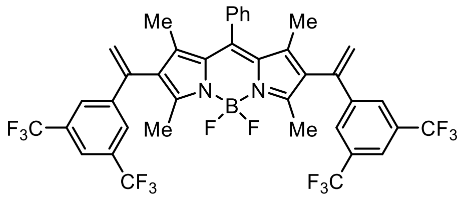

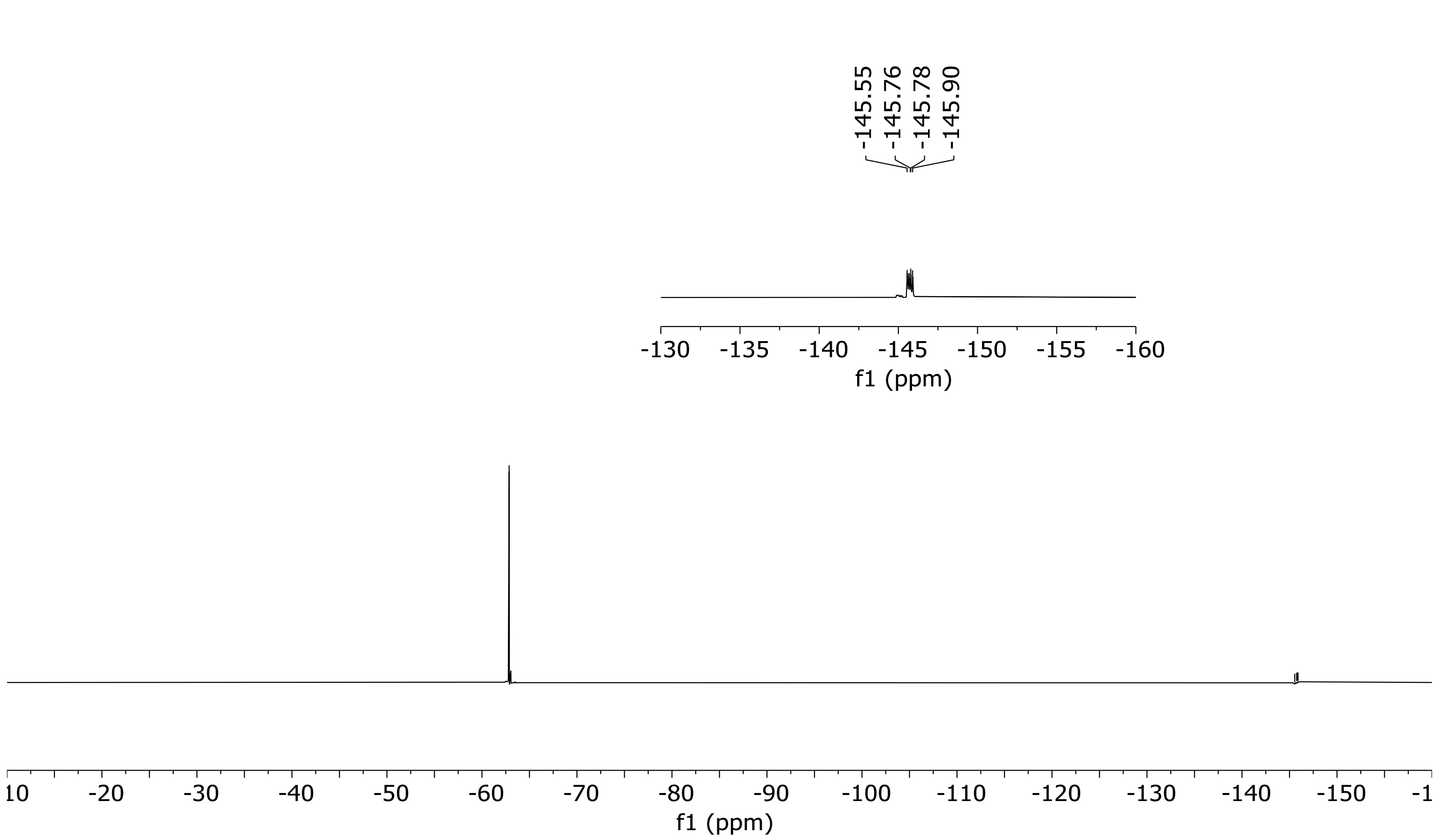

300 MHz ^{1}H-NMR Spectrum of compound **6a** ($CDCl_3$, 300 K)

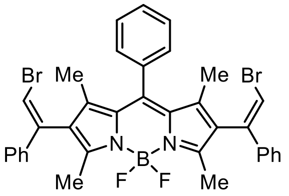

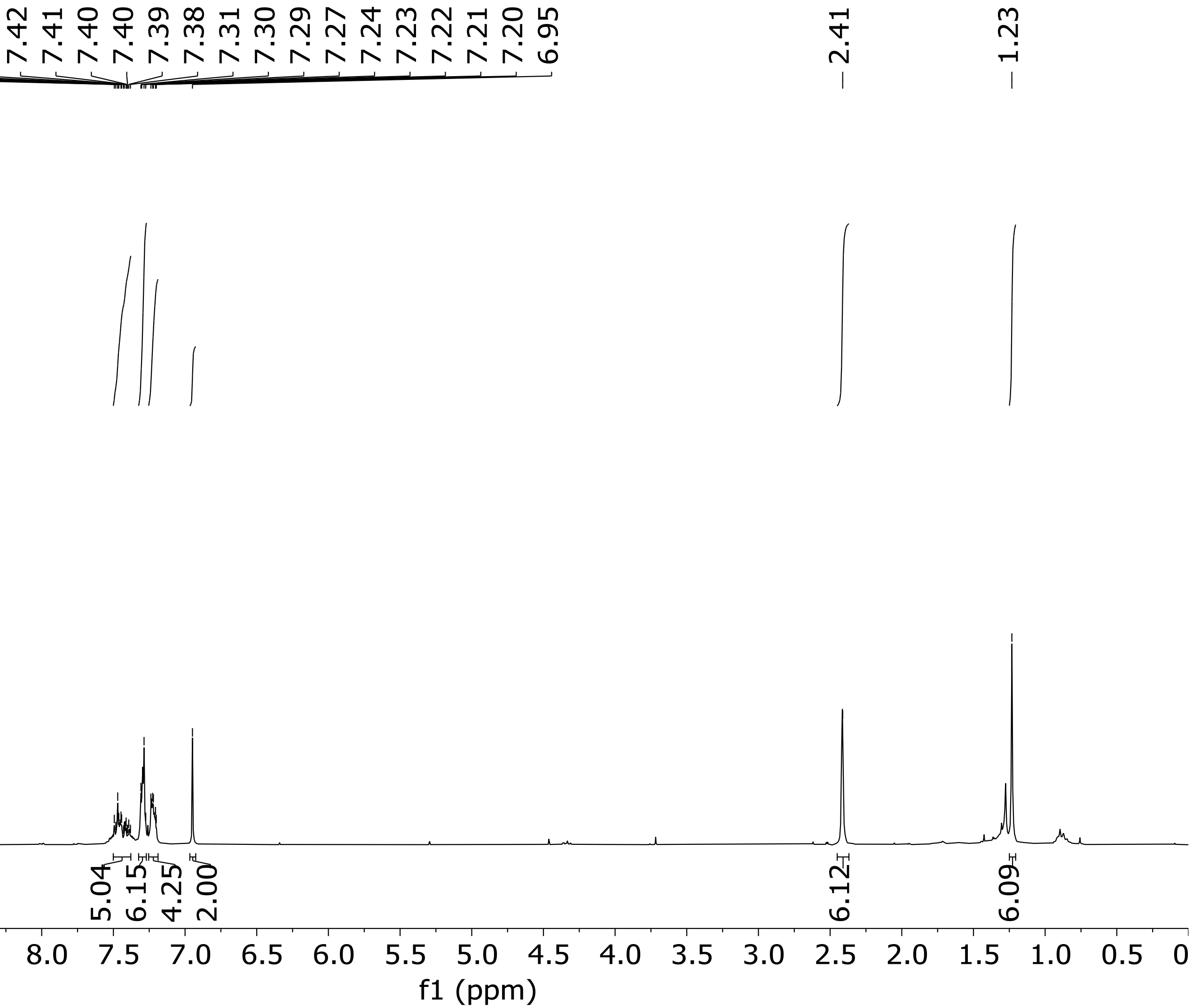

75 MHz $^{13}C\{^1H\}$-NMR Spectrum of compound **6a** ($CDCl_3$, 300 K)

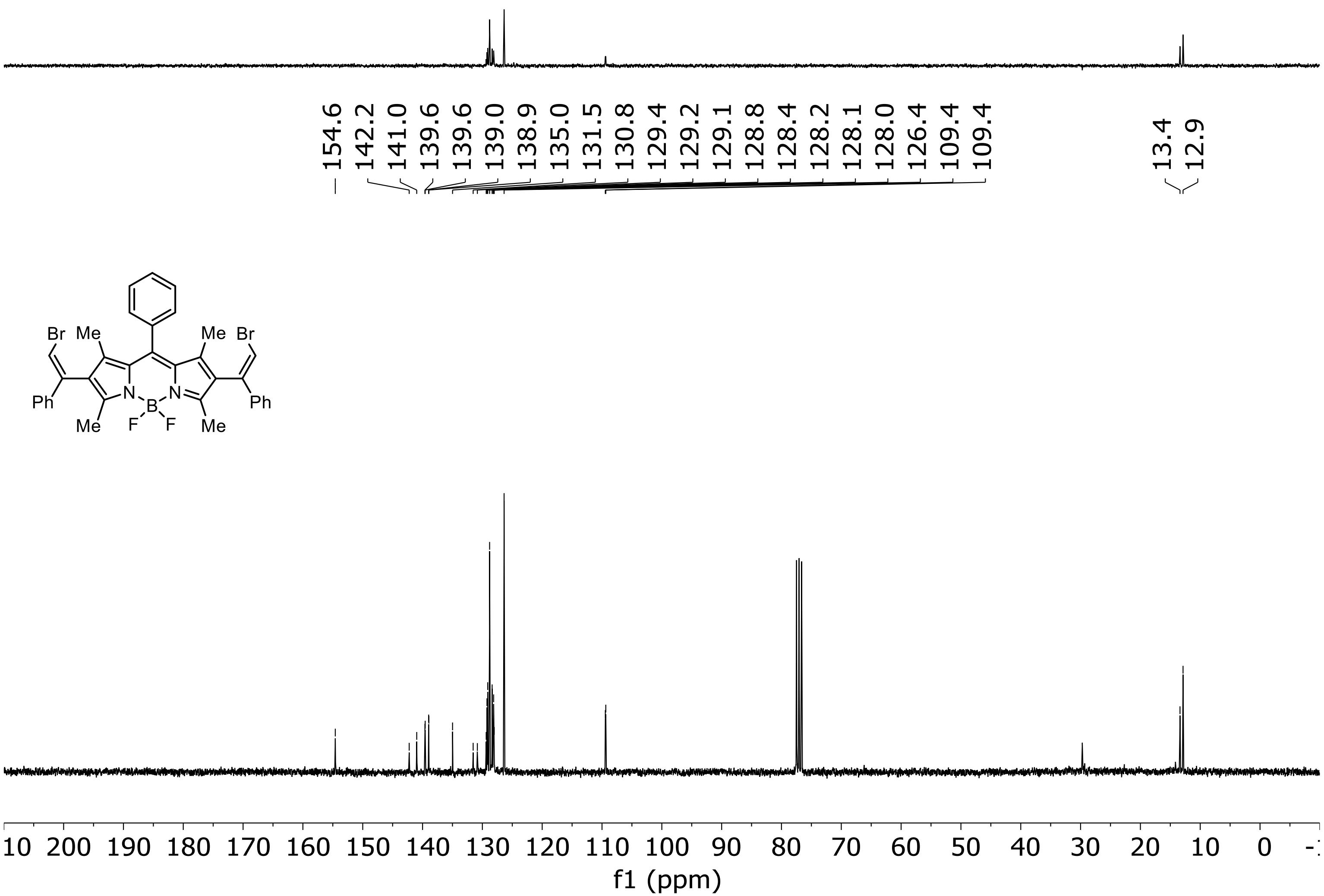

319 MHz ^{19}F-NMR Spectrum of compound **6a** ($CDCl_3$, 300 K)

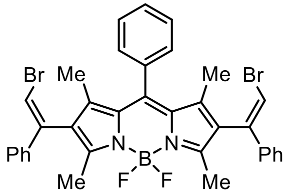

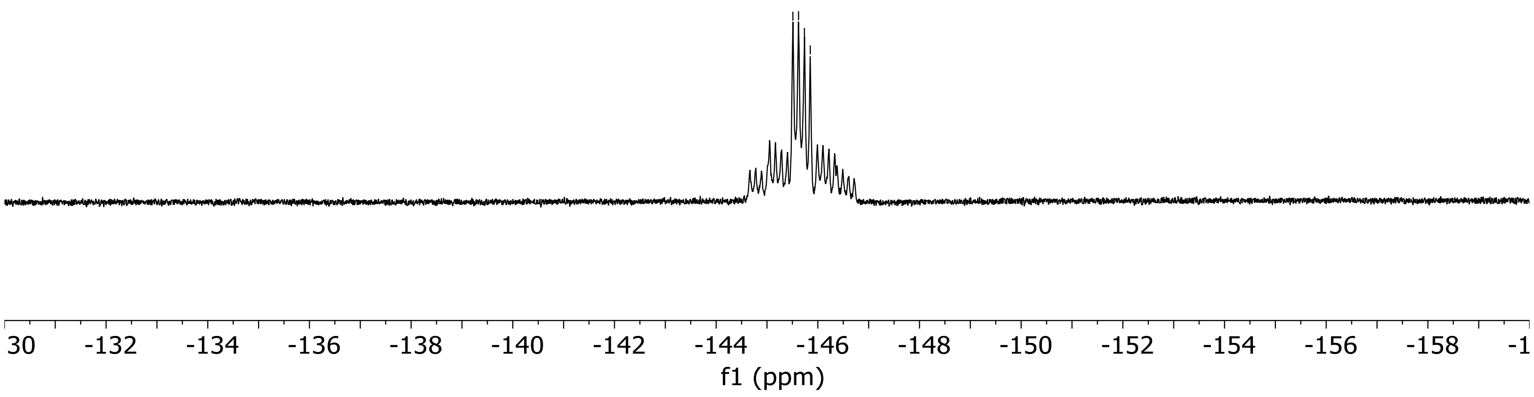

300 MHz NOESY Spectrum of compound **6a** ($CDCl_3$, 300 K)

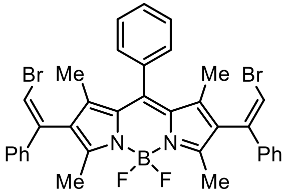

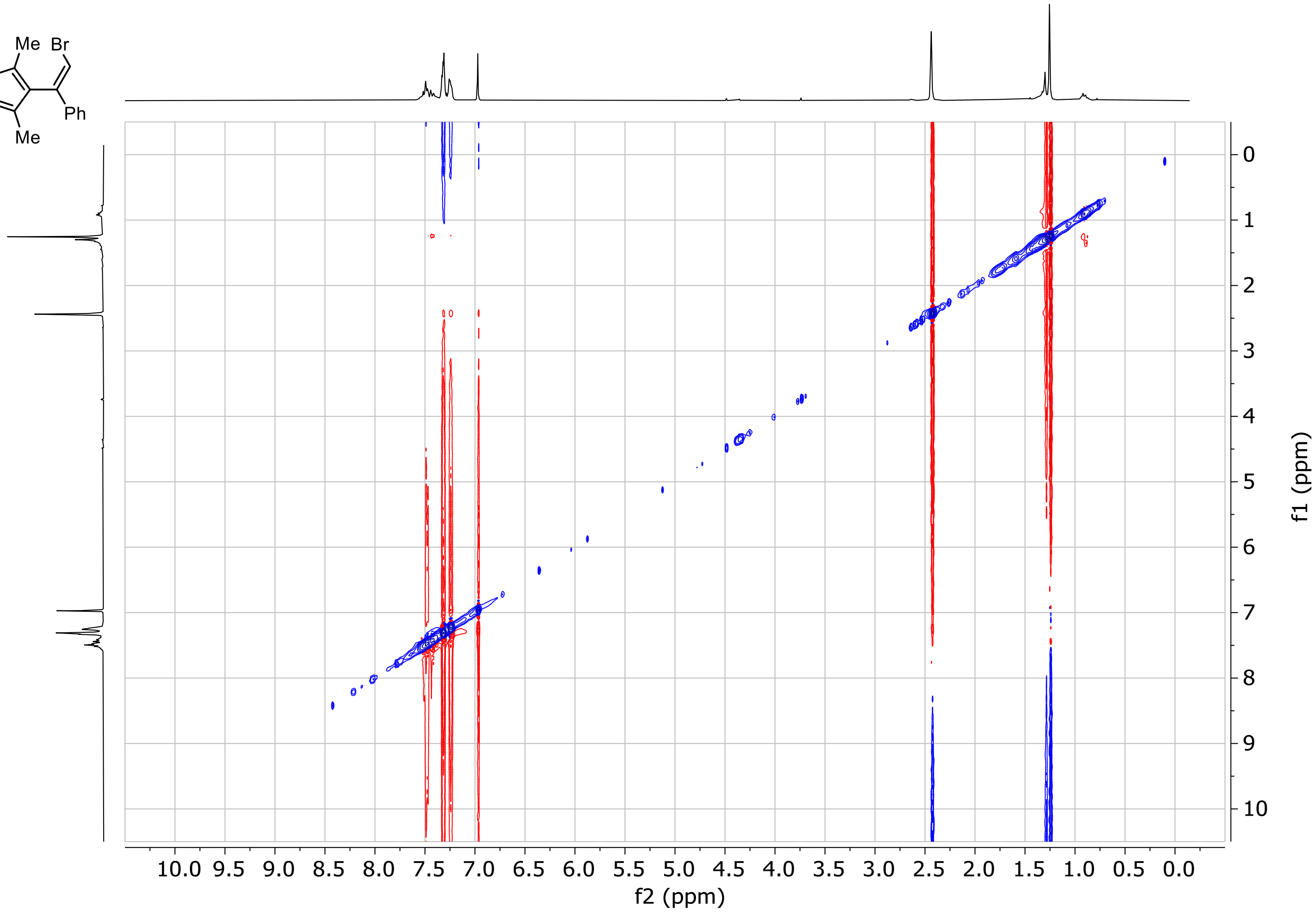

300 MHz 1D nOe Spectrum of compound **6a** ($CDCl_3$, 300 K) showing the correlation between the hydrogen of the alkenyl group at 6.95 ppm and the aromatic hydrogens at 7.23-7.28 ppm

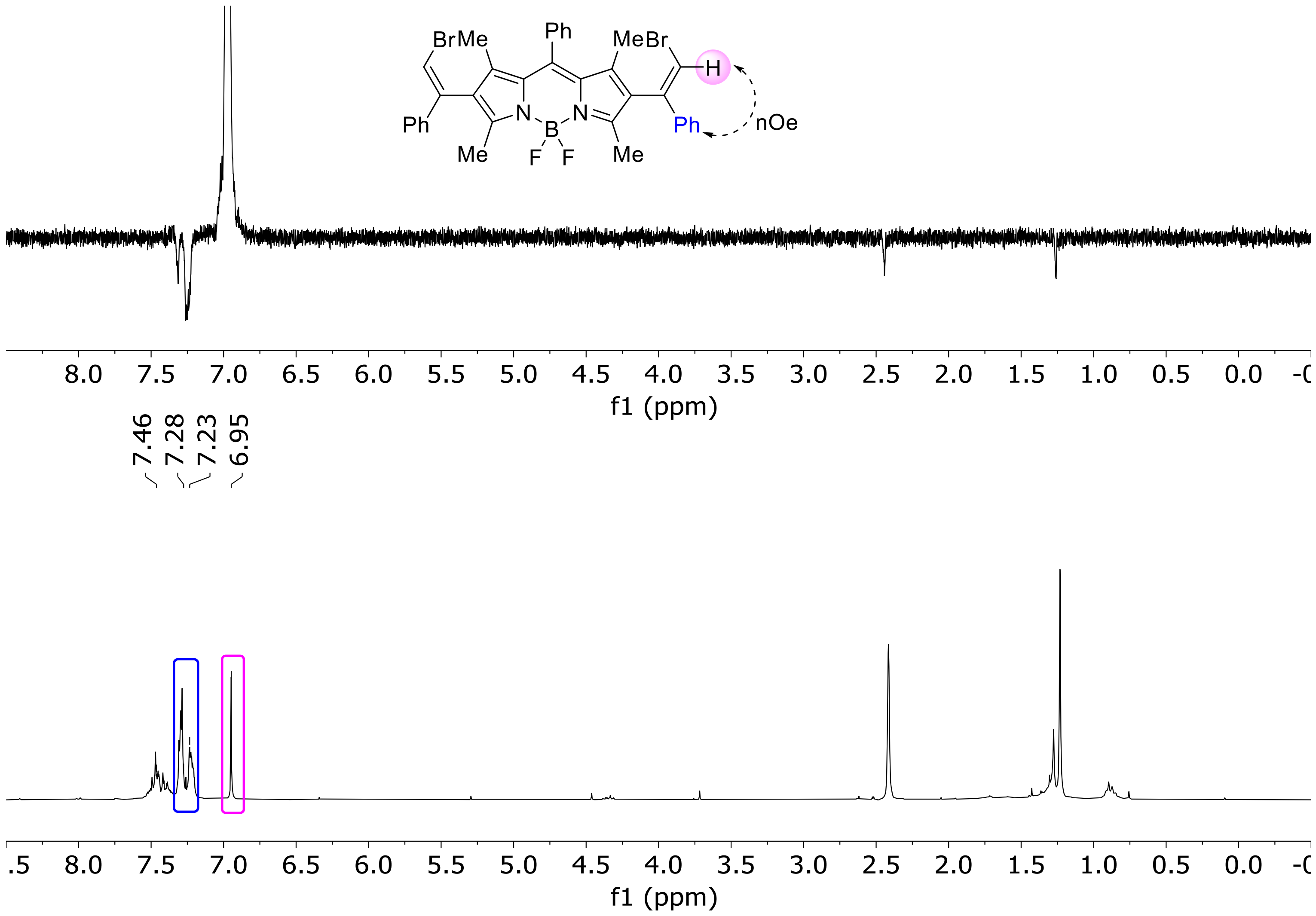

300 MHz ^{1}H-NMR Spectrum of compound **7a** ($CDCl_3$, 300 K)

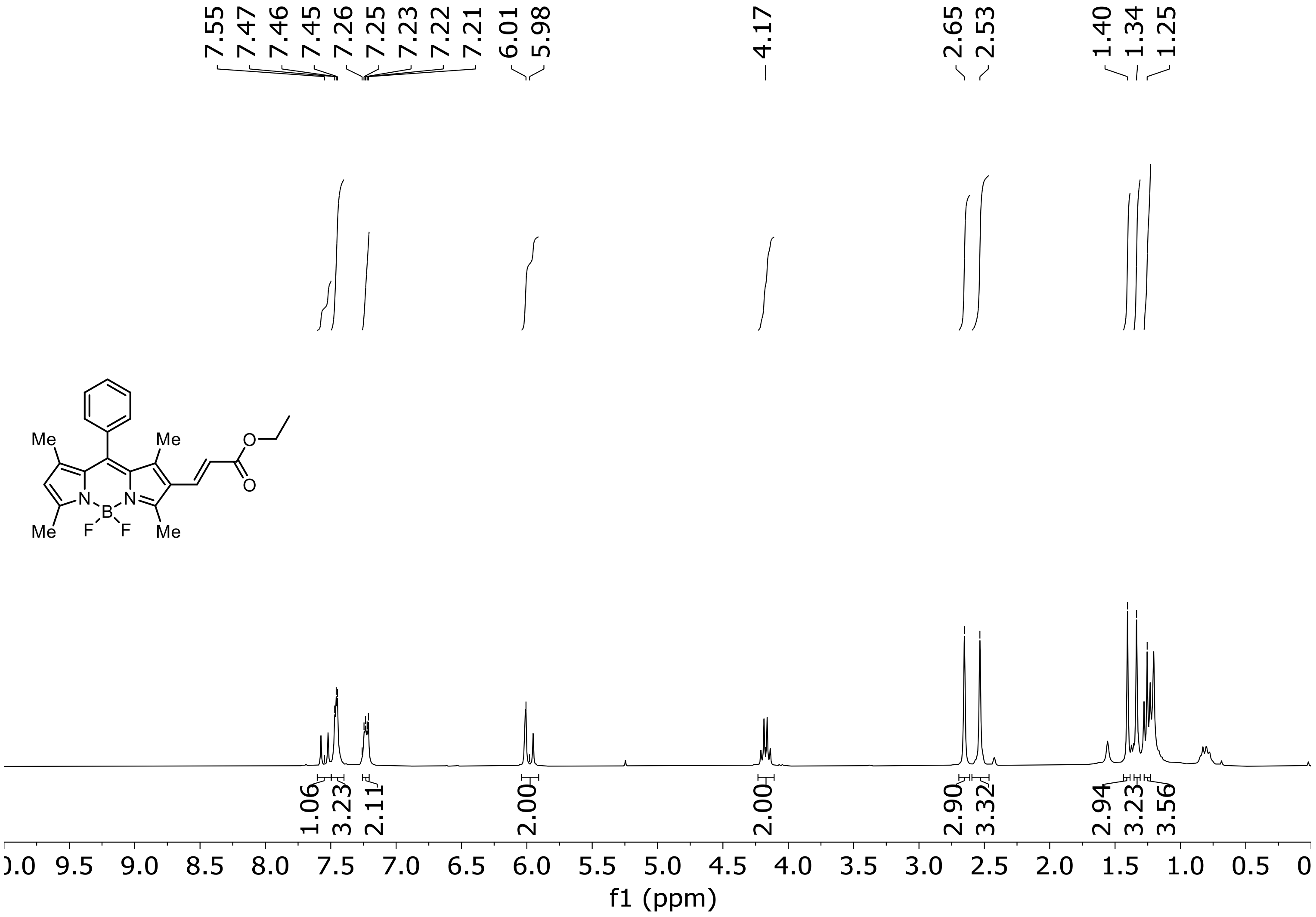

75 MHz $^{13}C\{^{1}H\}$-NMR Spectrum of compound **7a** ($CDCl_3$, 300 K)

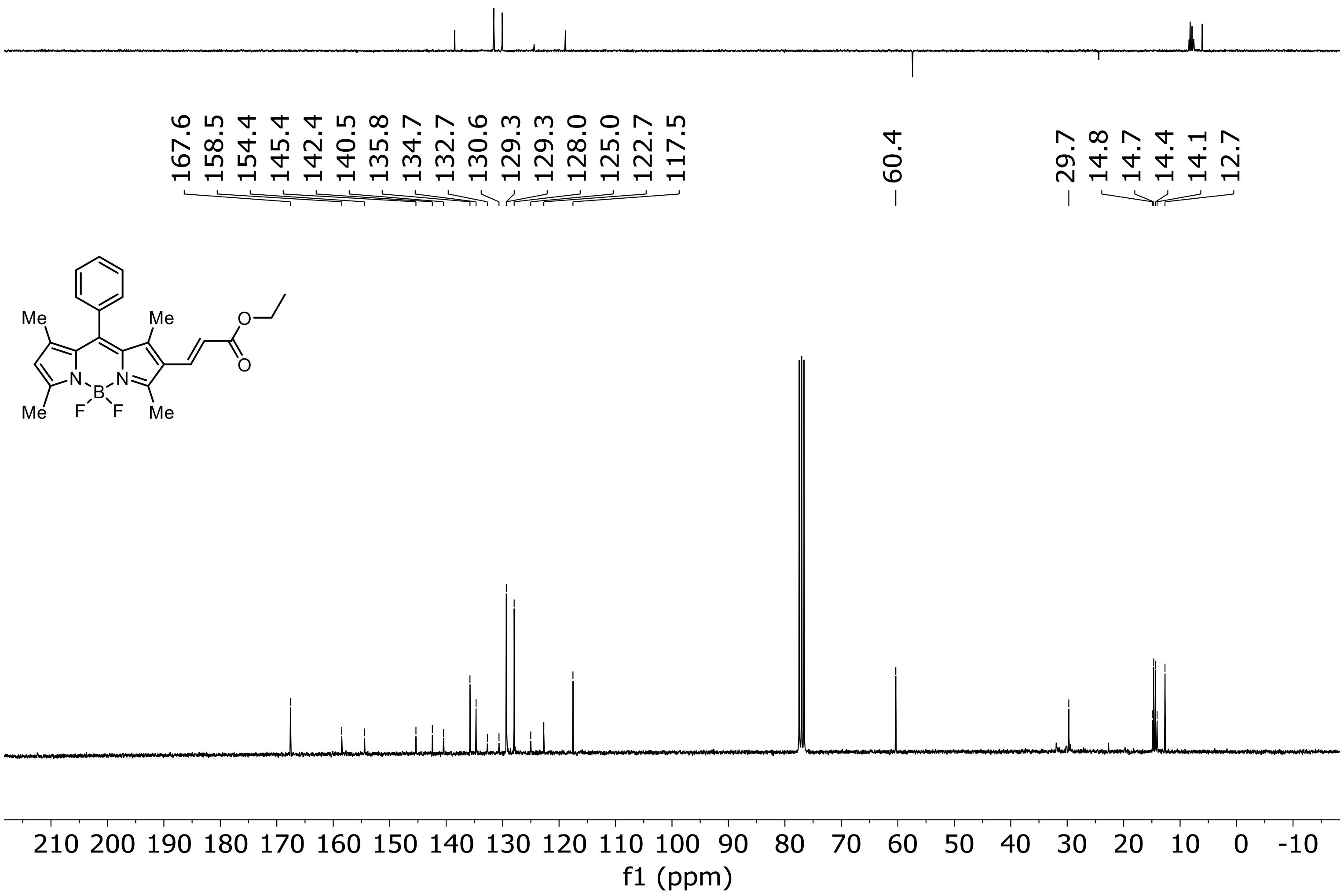

282 MHz ^{19}F-NMR Spectrum of compound **7a** ($CDCl_3$, 300 K)

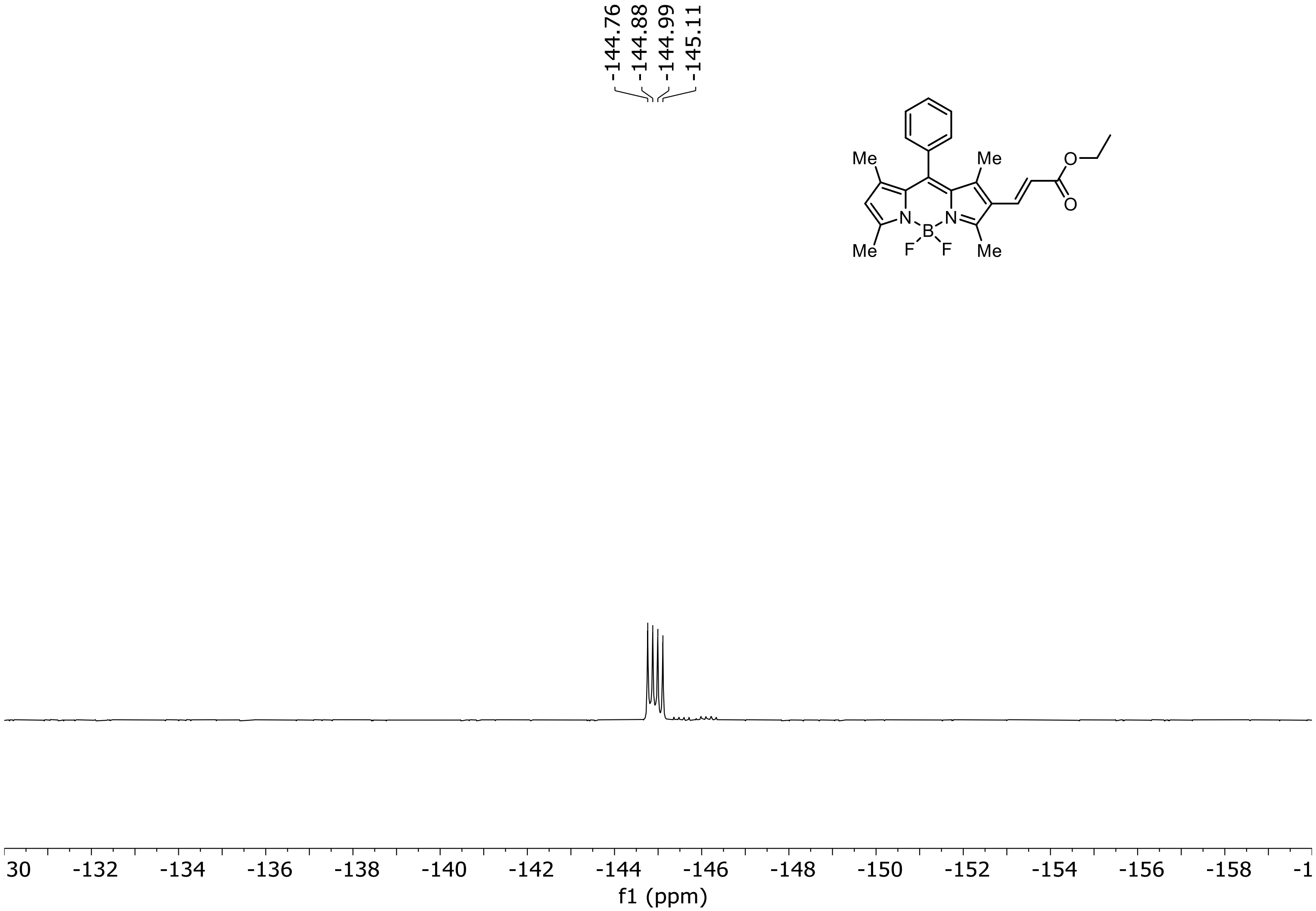

300 MHz ^{1}H-^{1}H COSY Spectrum of compound **7a** ($CDCl_3$, 300 K)

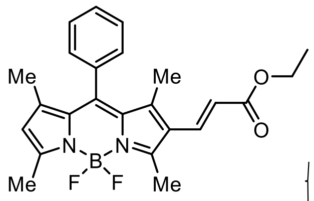

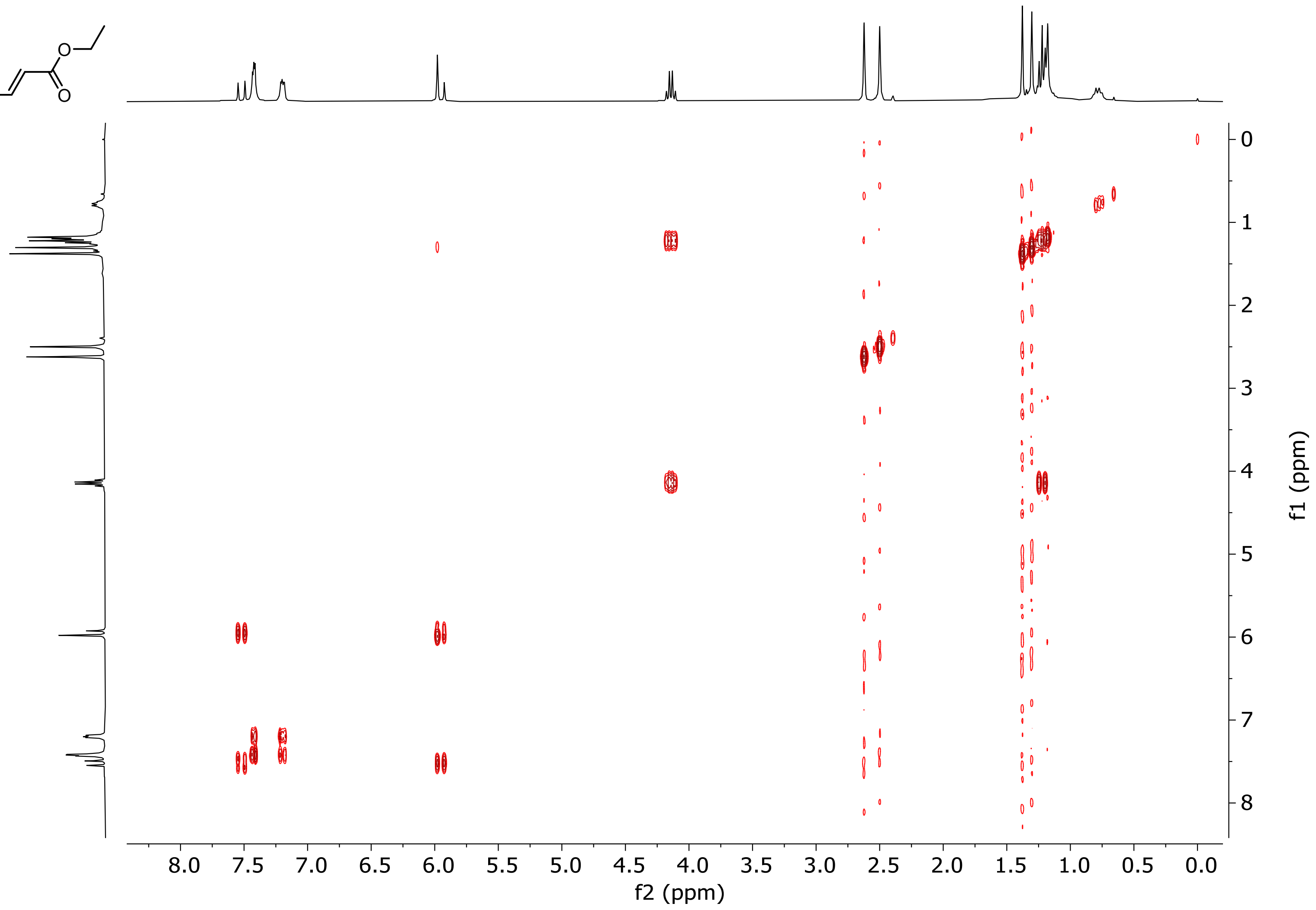

300 MHz HSQC Spectrum of compound **7a** ($CDCl_3$, 300 K)

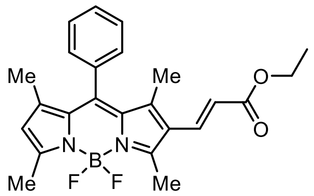

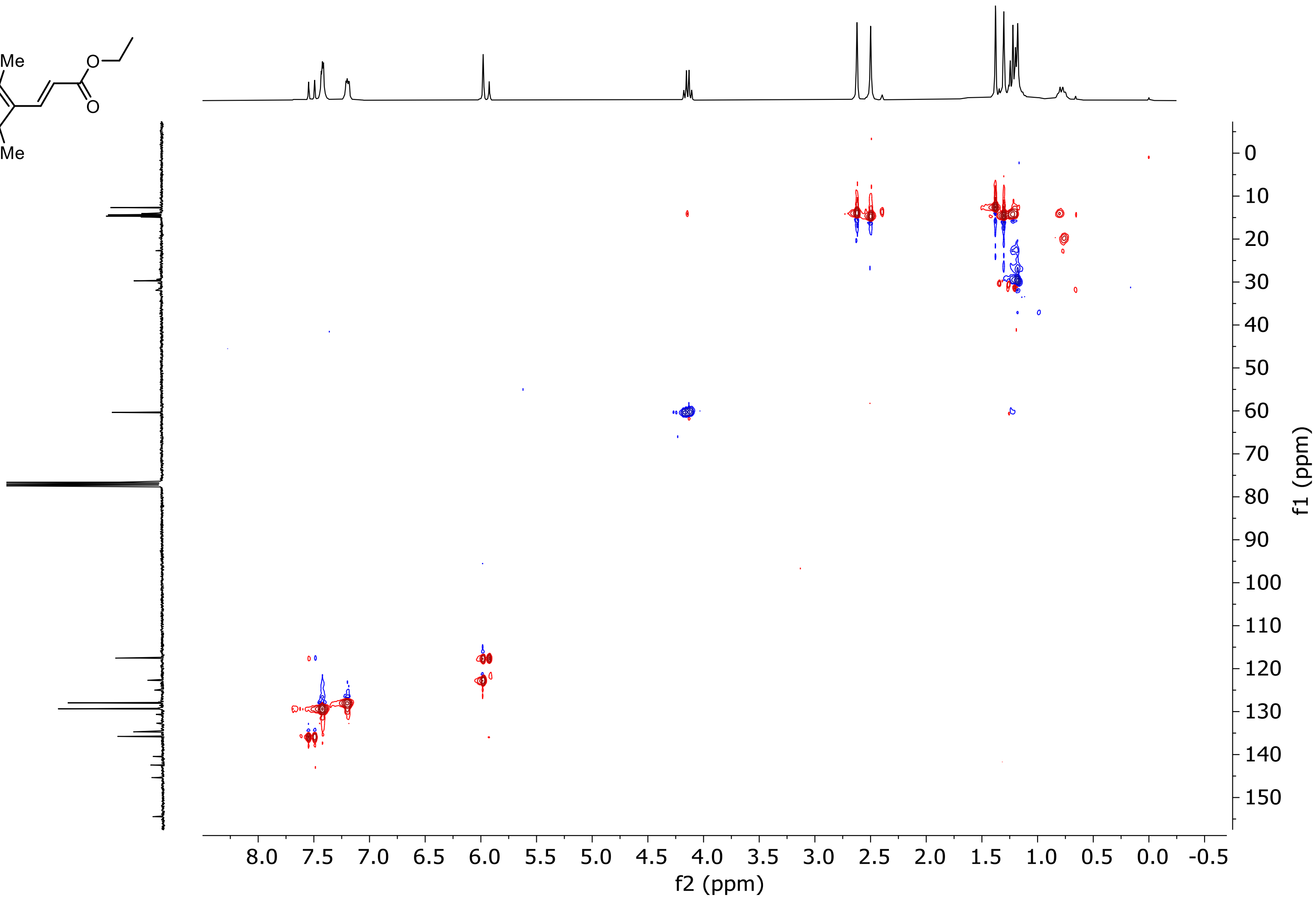

300 MHz HMBC Spectrum of compound **7a** ($CDCl_3$, 300 K)

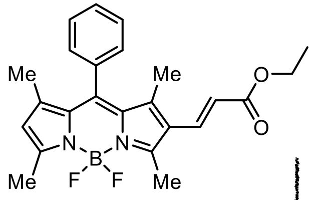

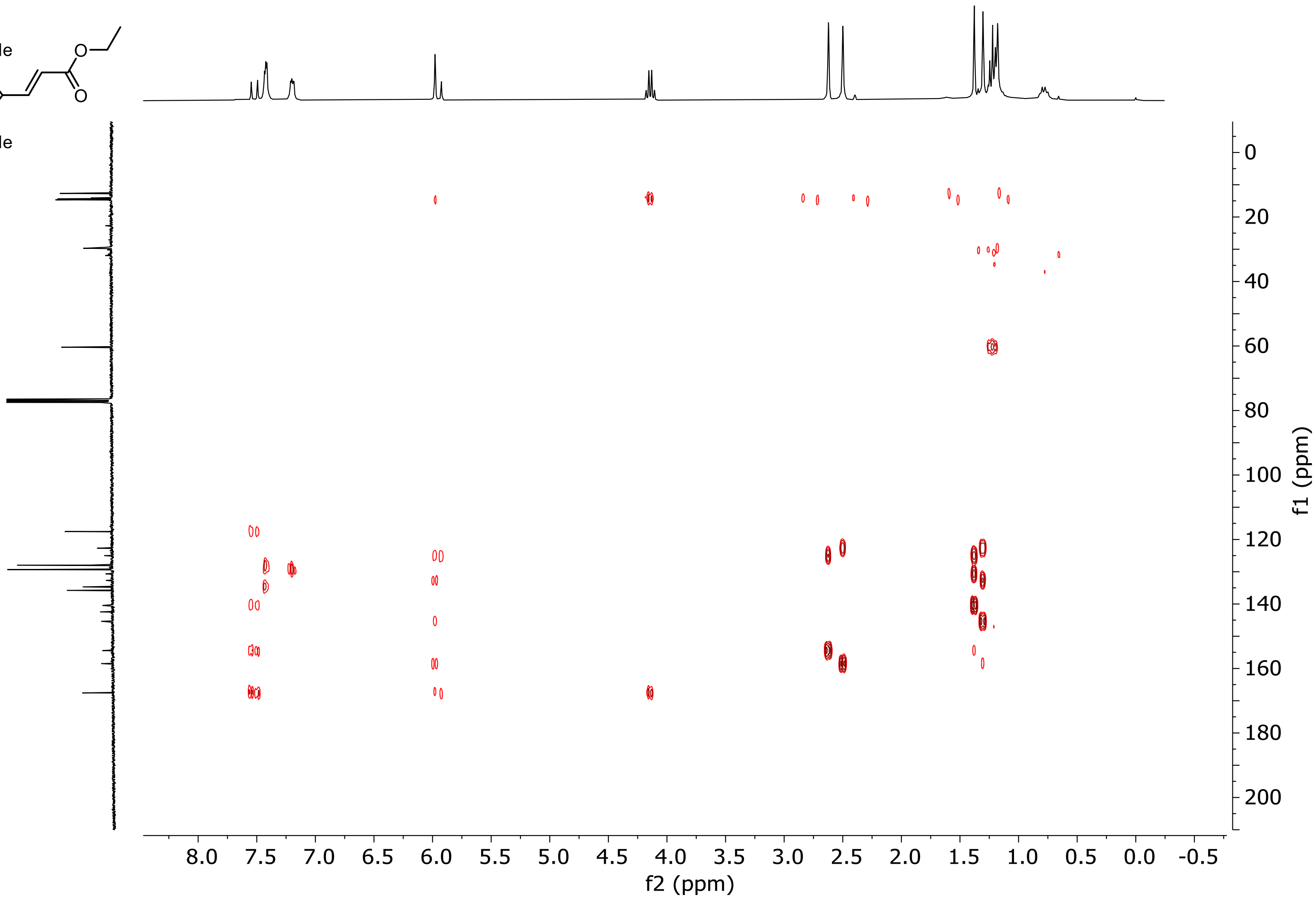

300 MHz NOESY Spectrum of compound **7a** ($CDCl_3$, 300 K)

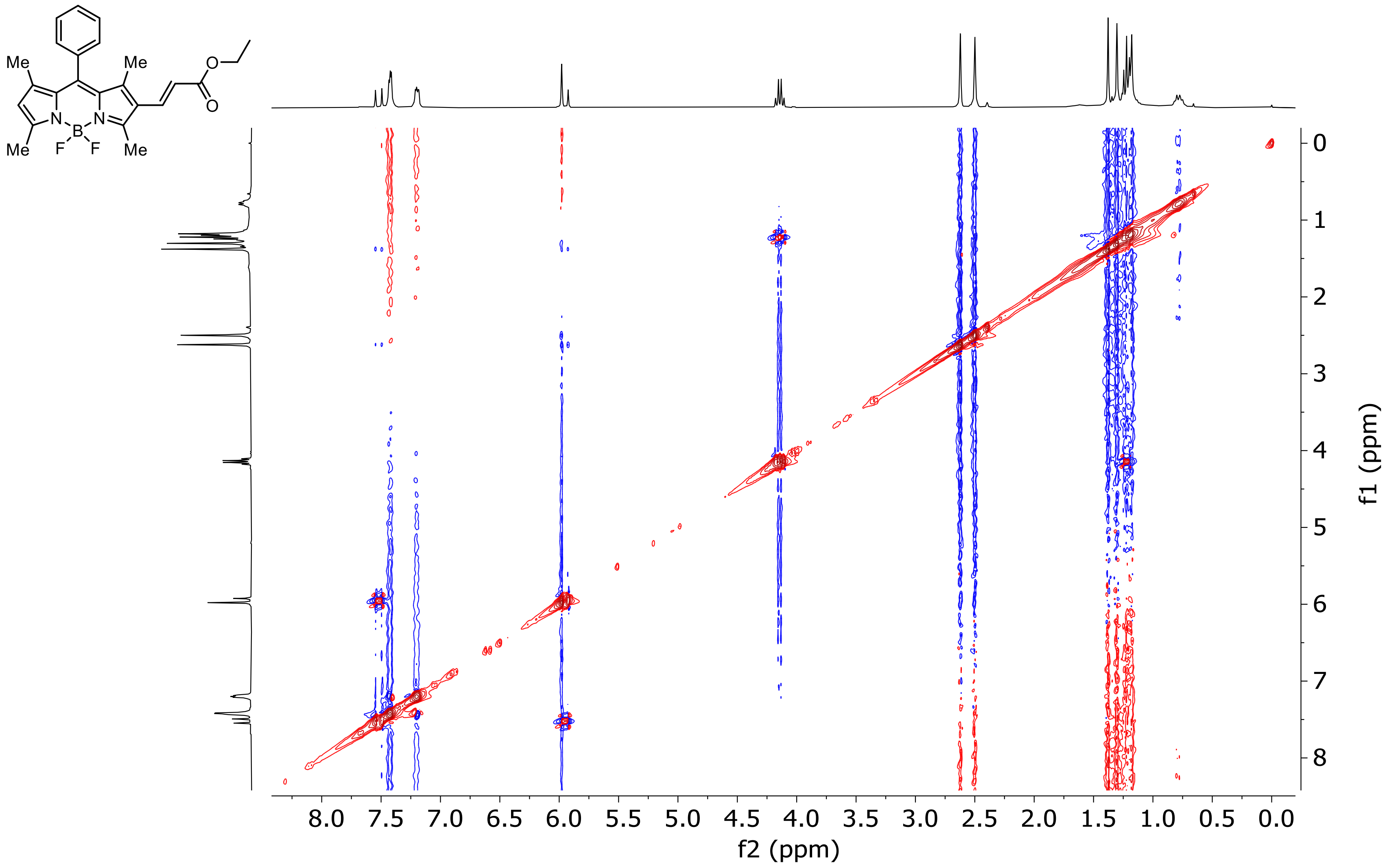

300 MHz [1]D nOe Spectrum of compound **7a** ($CDCl_3$, 300 K) showing the correlation between the hydrogen of the alkenyl group at 6.03 ppm and the methyl hydrogens at 2.60 ppm and the correlation between the hydrogen of the alkenyl group at 7.63 ppm and the methyl hydrogens at 1.41 ppm.

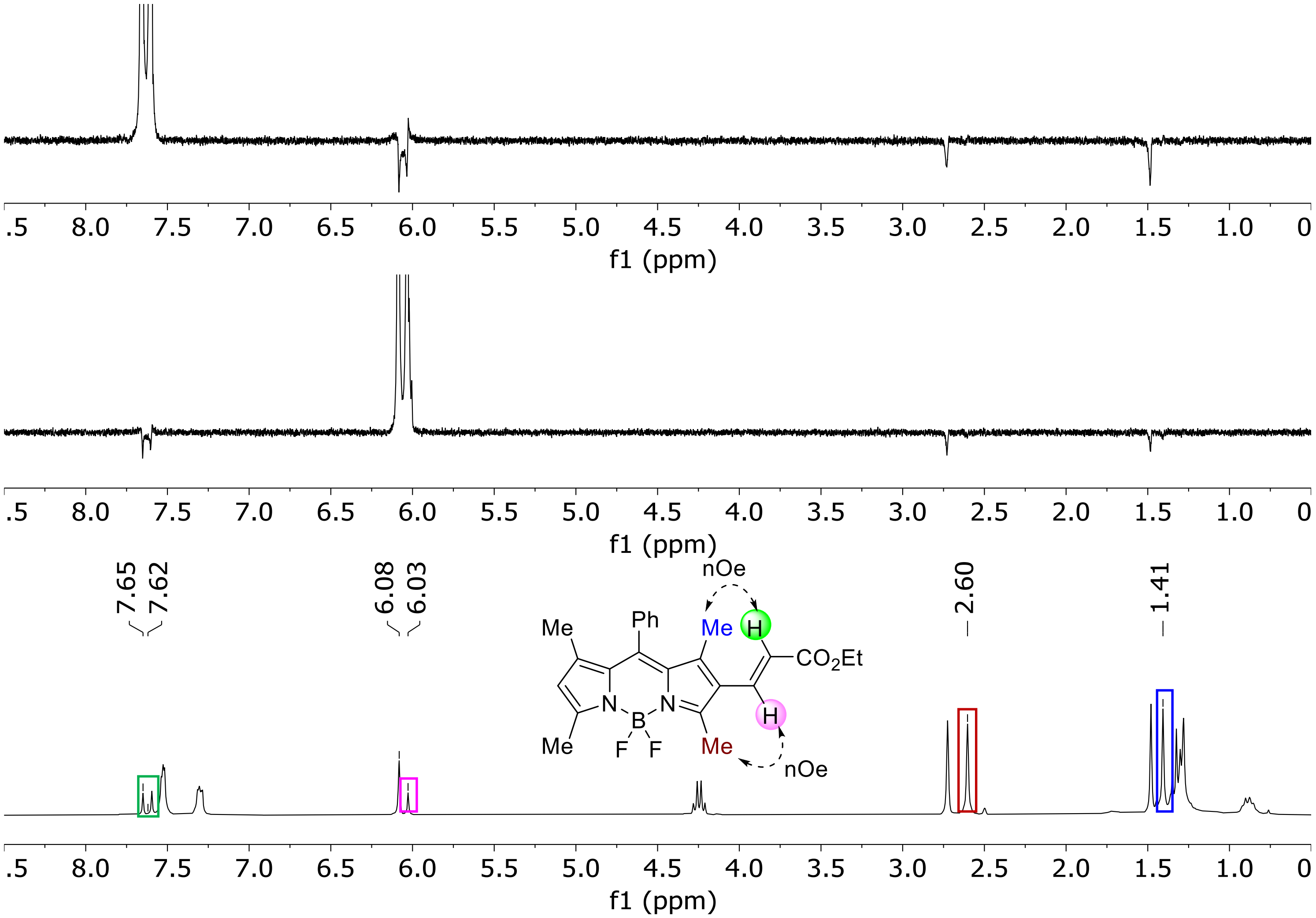

300 MHz ^{1}H-NMR Spectrum of compound **7b** ($CDCl_3$, 300 K)

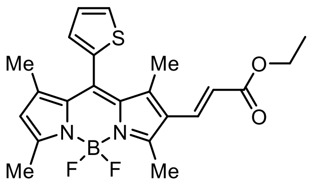

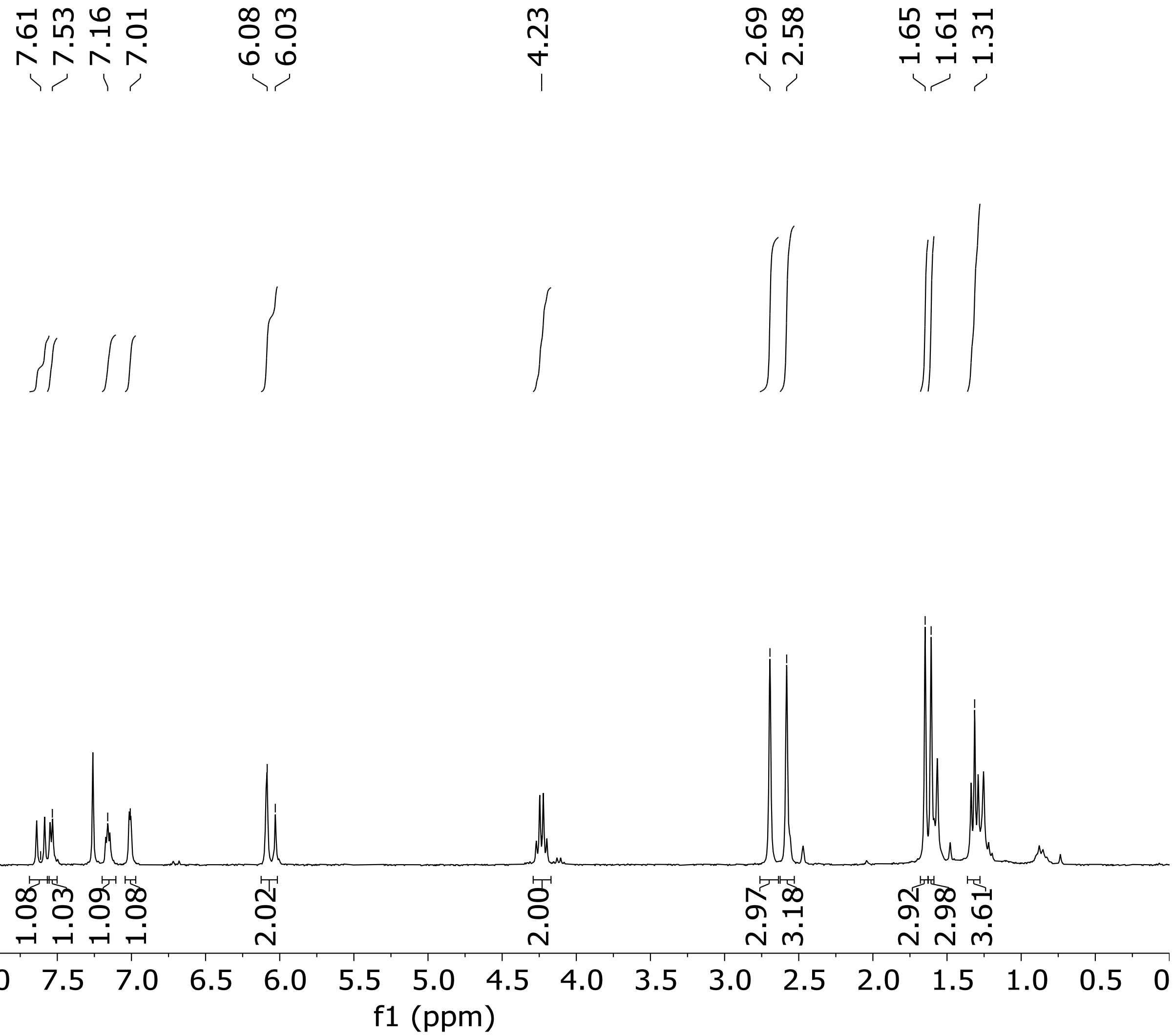

75 MHz $^{13}C\{^1H\}$-NMR Spectrum of compound **7b** ($CDCl_3$, 300 K)

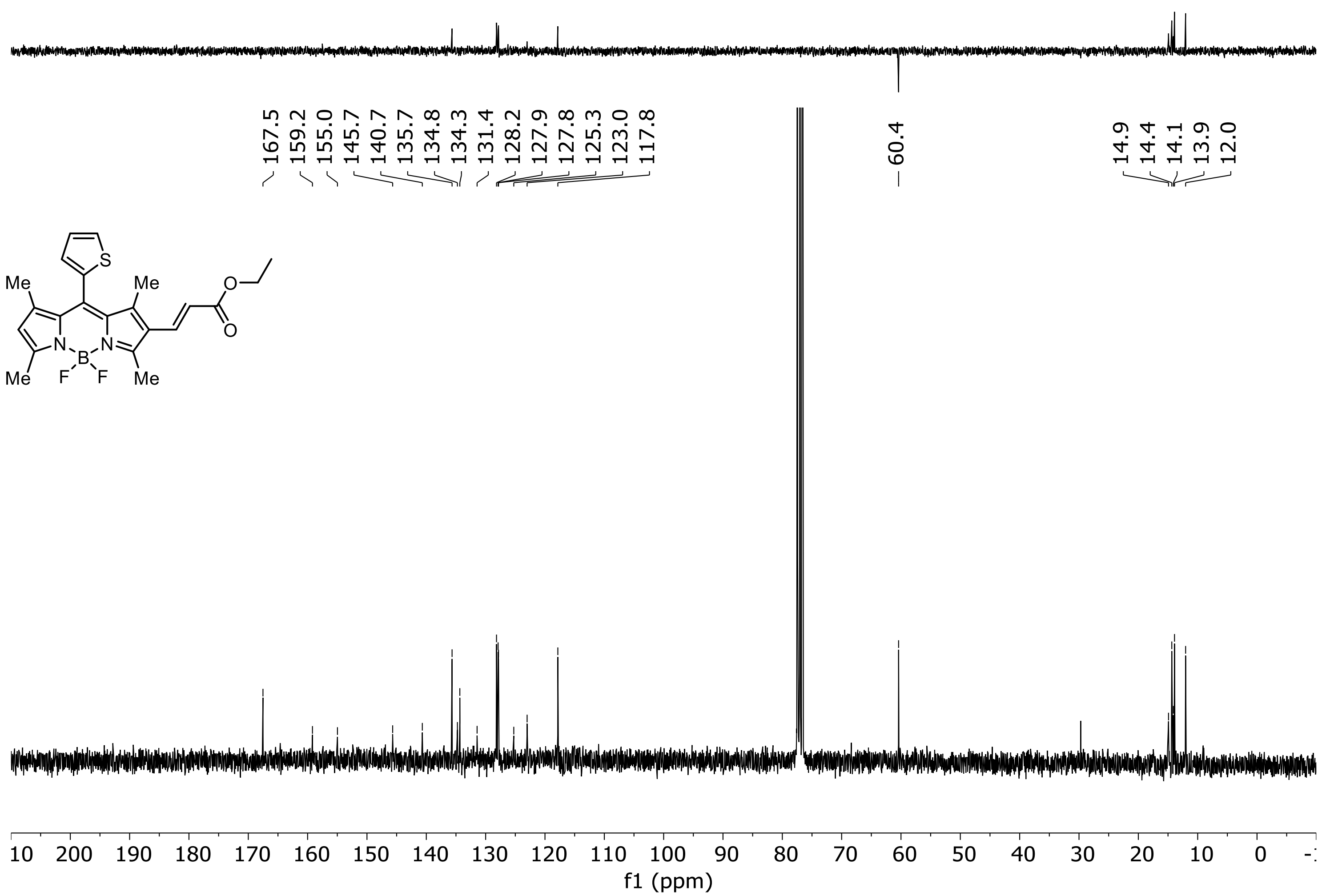

282 MHz ^{19}F-NMR Spectrum of compound **7b** ($CDCl_3$, 300 K)

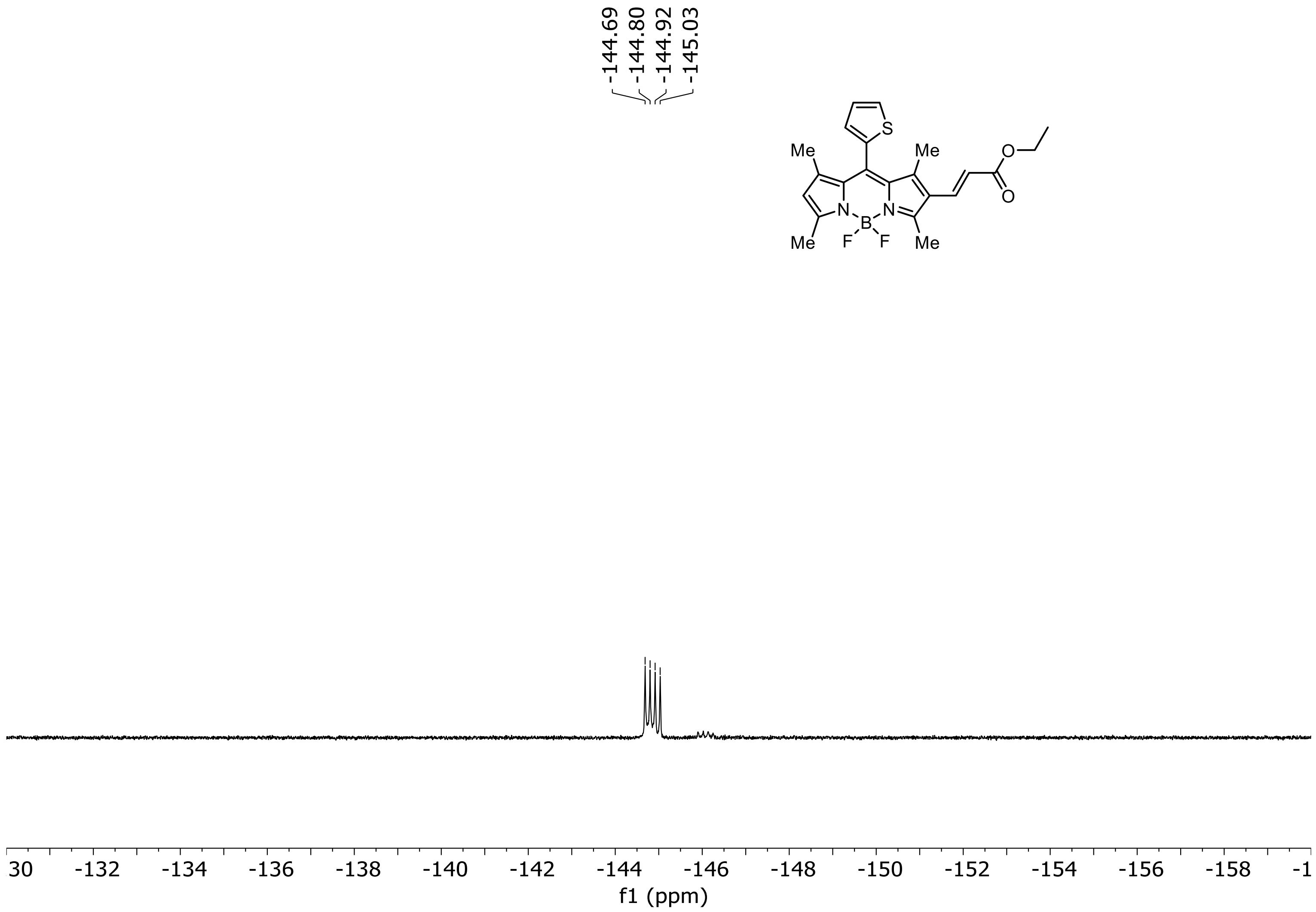

300 MHz ¹H-NMR Spectrum of compound **8a** ($CDCl_3$, 300 K)

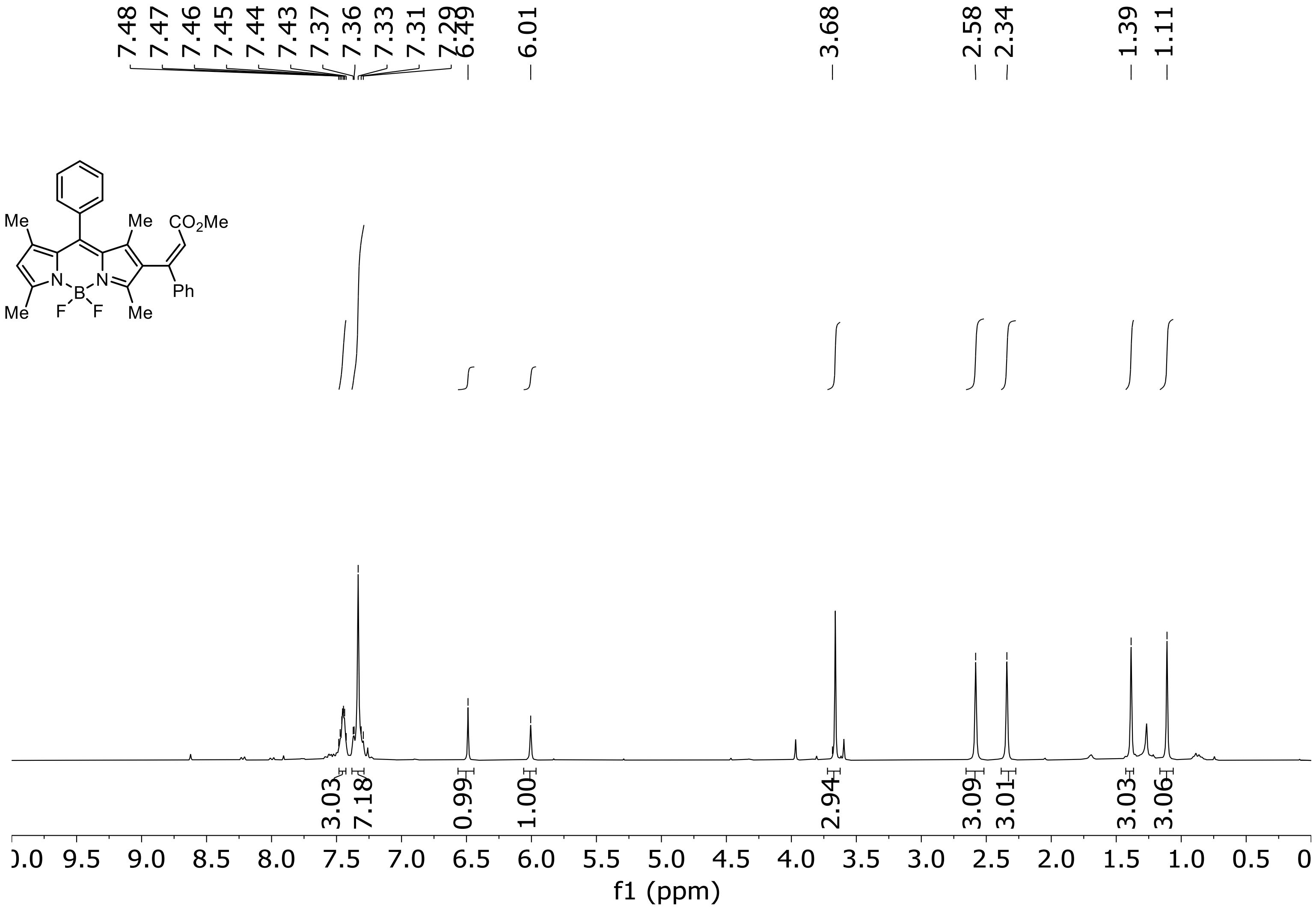

75 MHz $^{13}C\{^{1}H\}$-NMR Spectrum of compound **8a** ($CDCl_3$, 300 K)

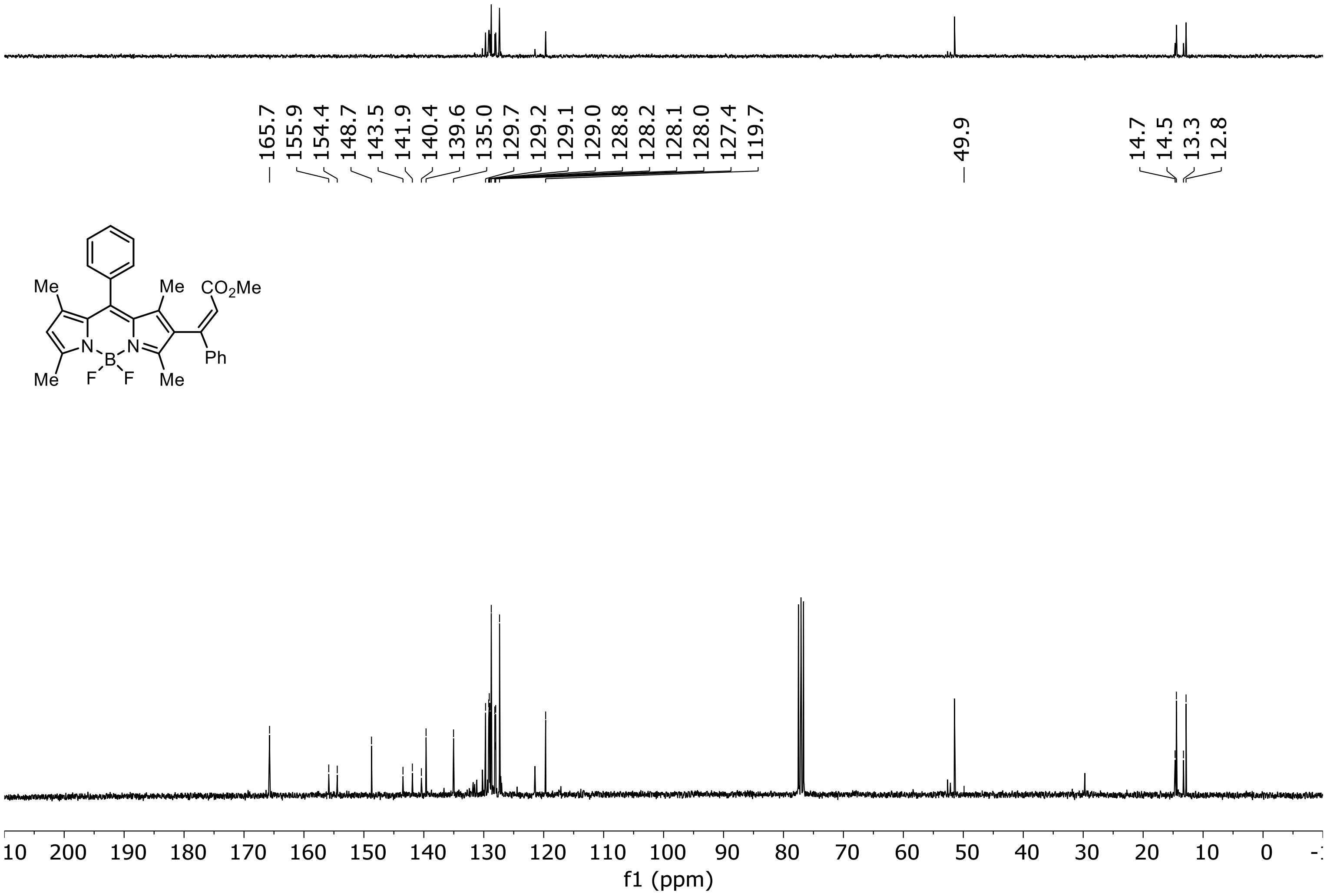

282 MHz ^{19}F-NMR Spectrum of compound **8a** ($CDCl_3$, 300 K)

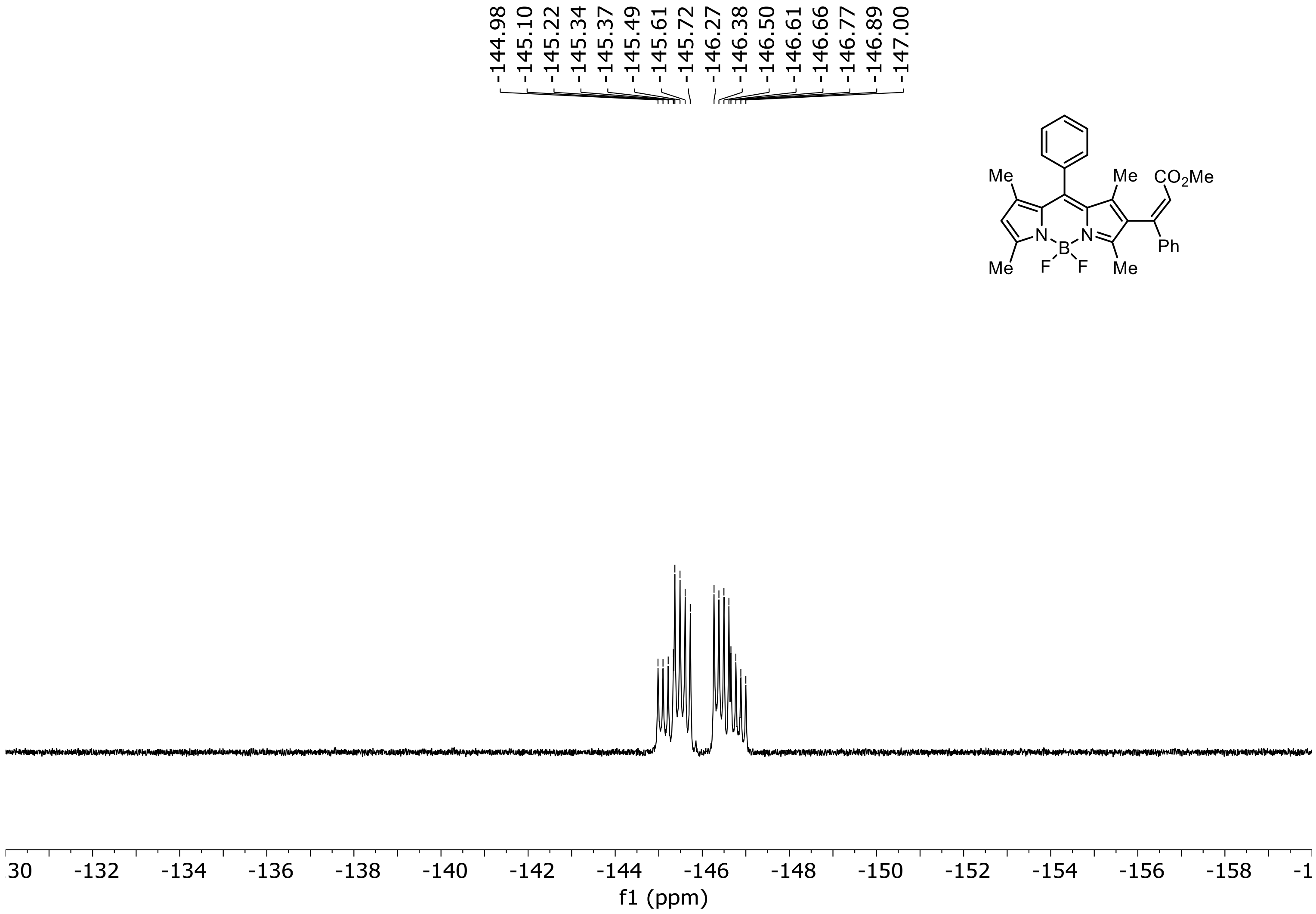

300 MHz ^{1}H-^{1}H COSY Spectrum of compound **8a** (CDCl$_3$, 300 K)

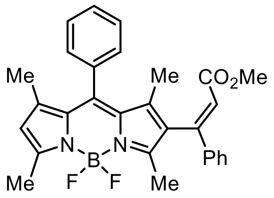

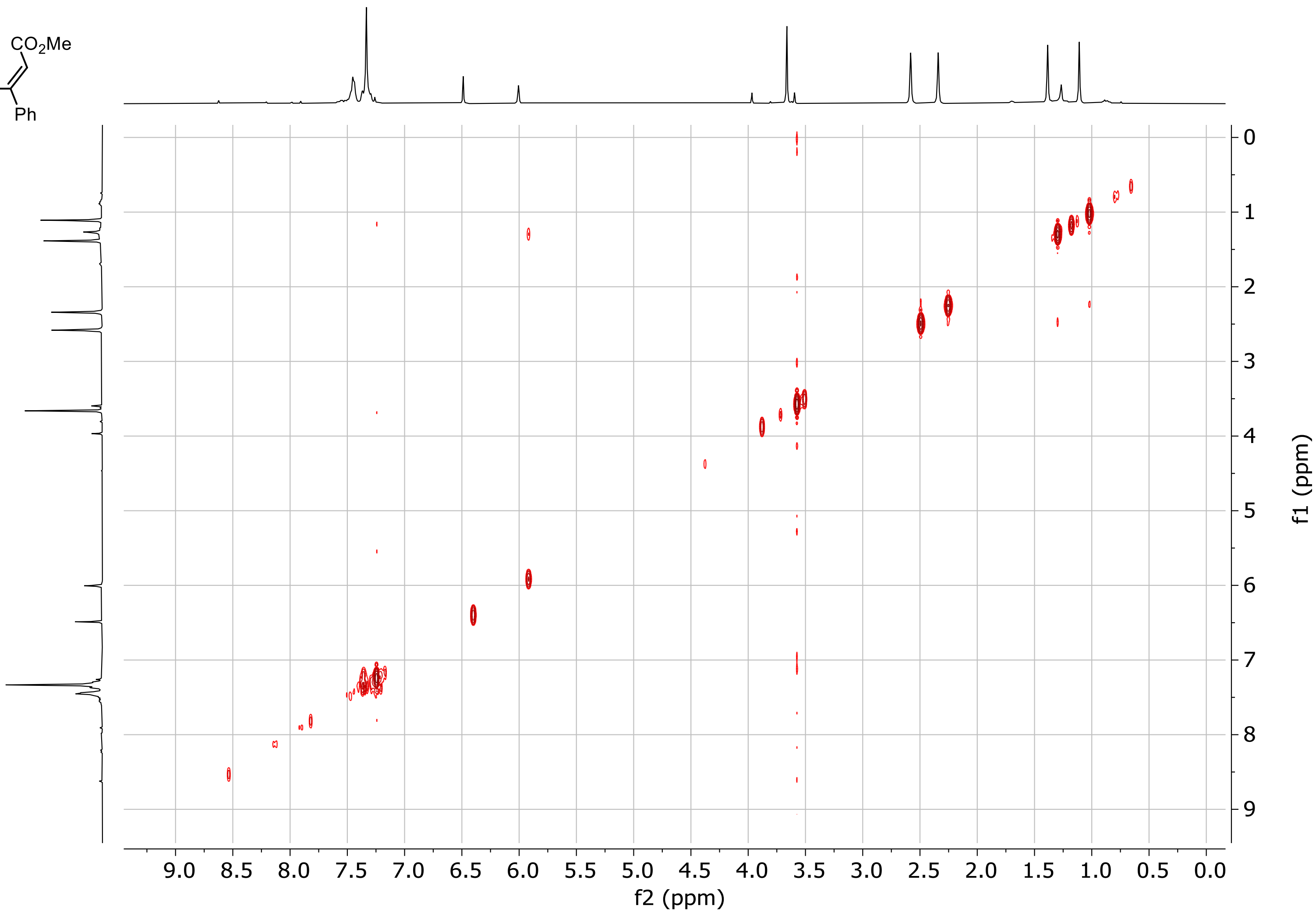

300 MHz HSQC Spectrum of compound **8a** ($CDCl_3$, 300 K)

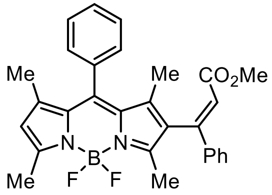

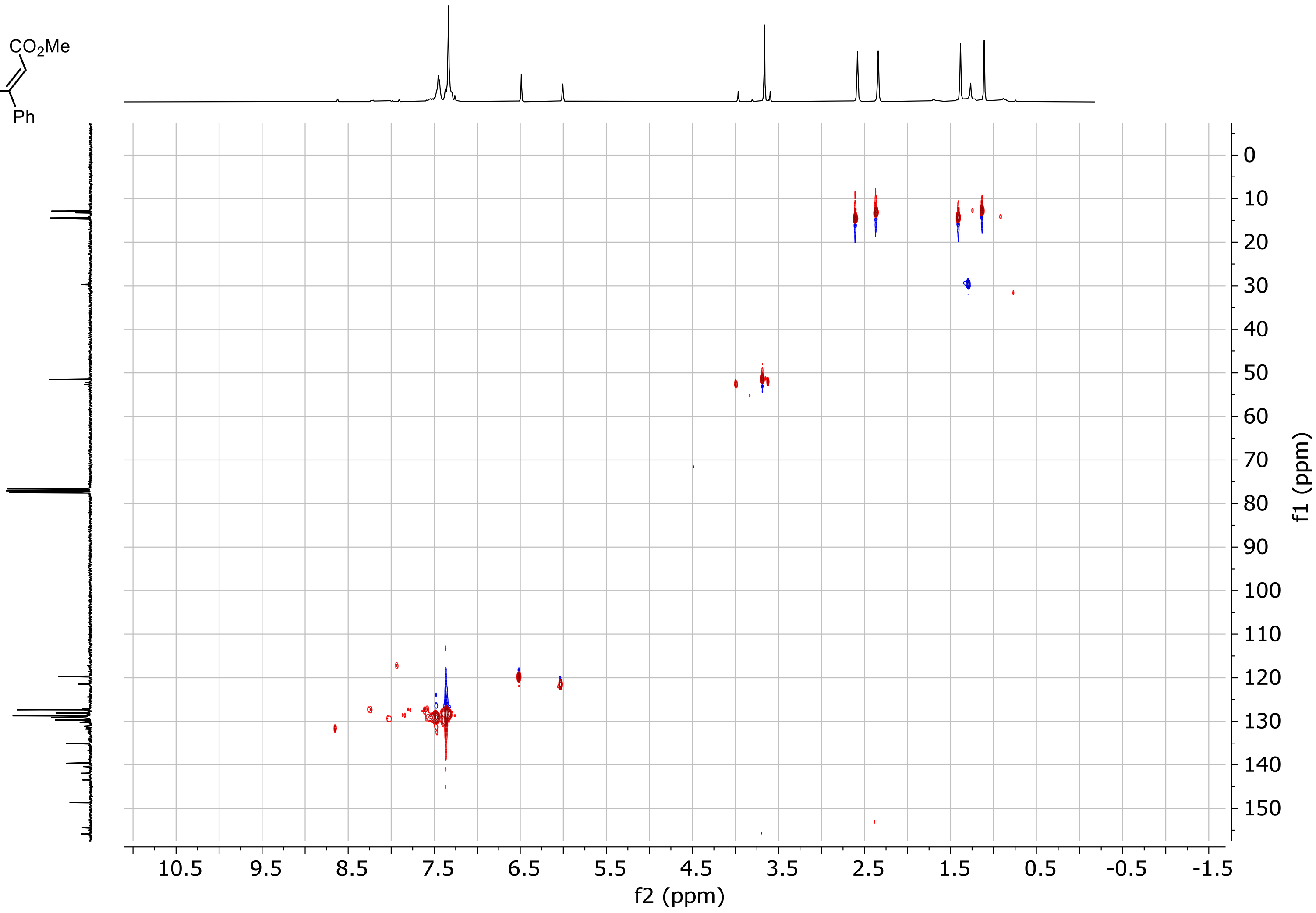

300 MHz HMBC Spectrum of compound **8a** ($CDCl_3$, 300 K)

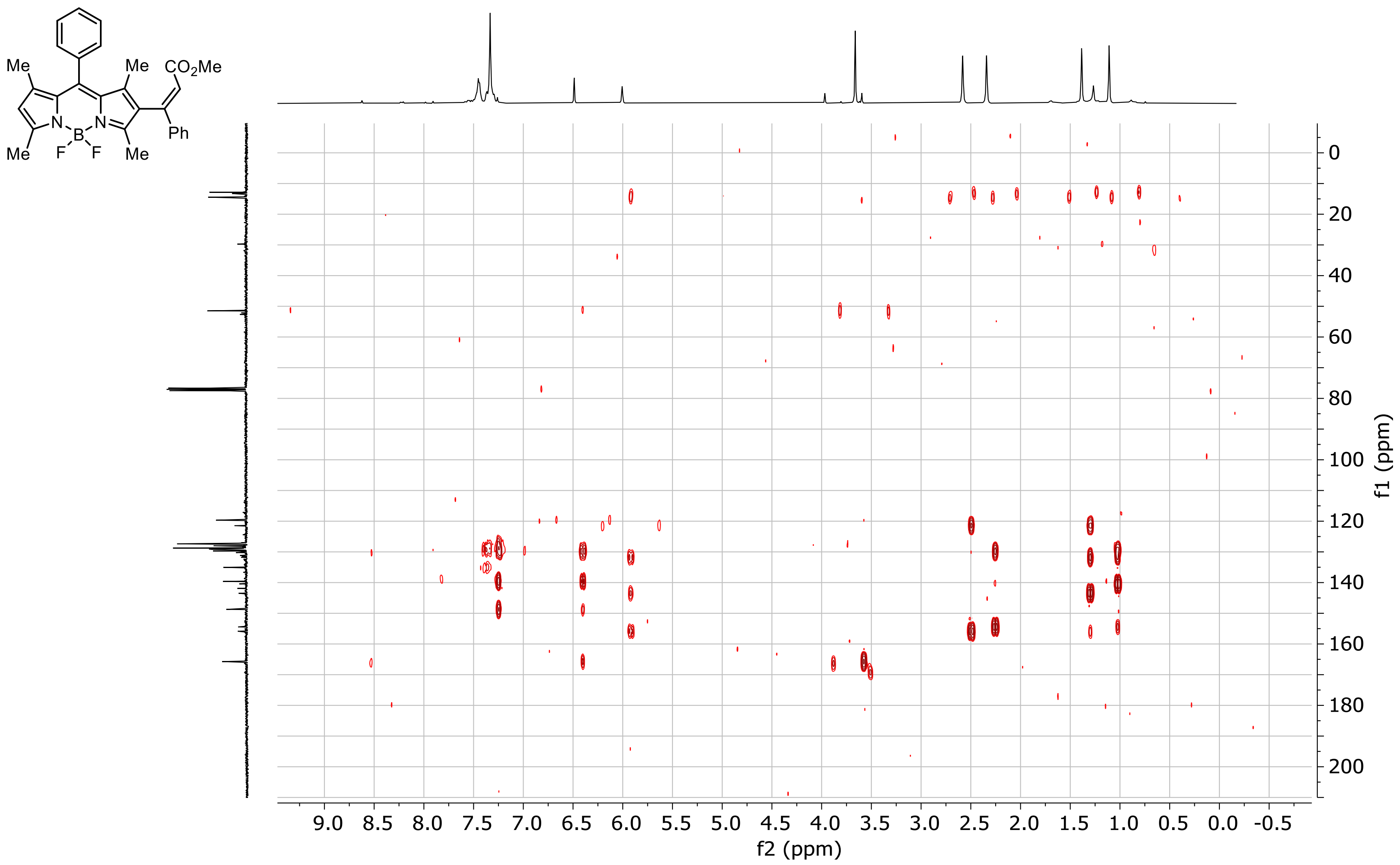

300 MHz NOESY Spectrum of compound **8a** ($CDCl_3$, 300 K)

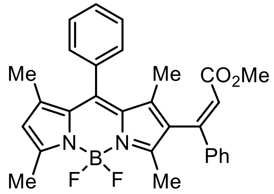

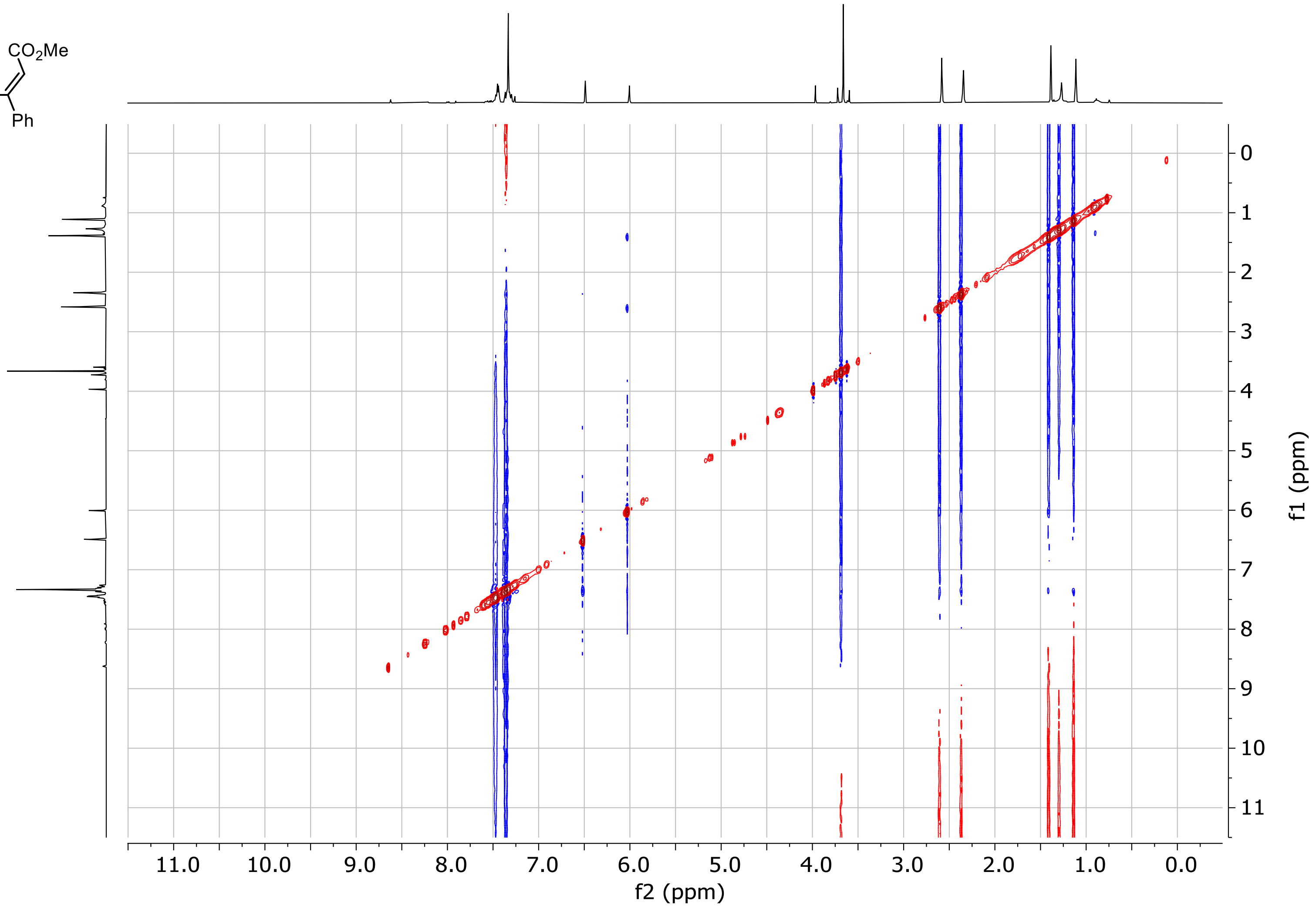

300 MHz [1]D nOe Spectrum of compound **8a** ($CDCl_3$, 300 K) showing the correlation between the hydrogen of the alkenyl group at 6.51 ppm and the aromatic hydrogens at 7.36 ppm.

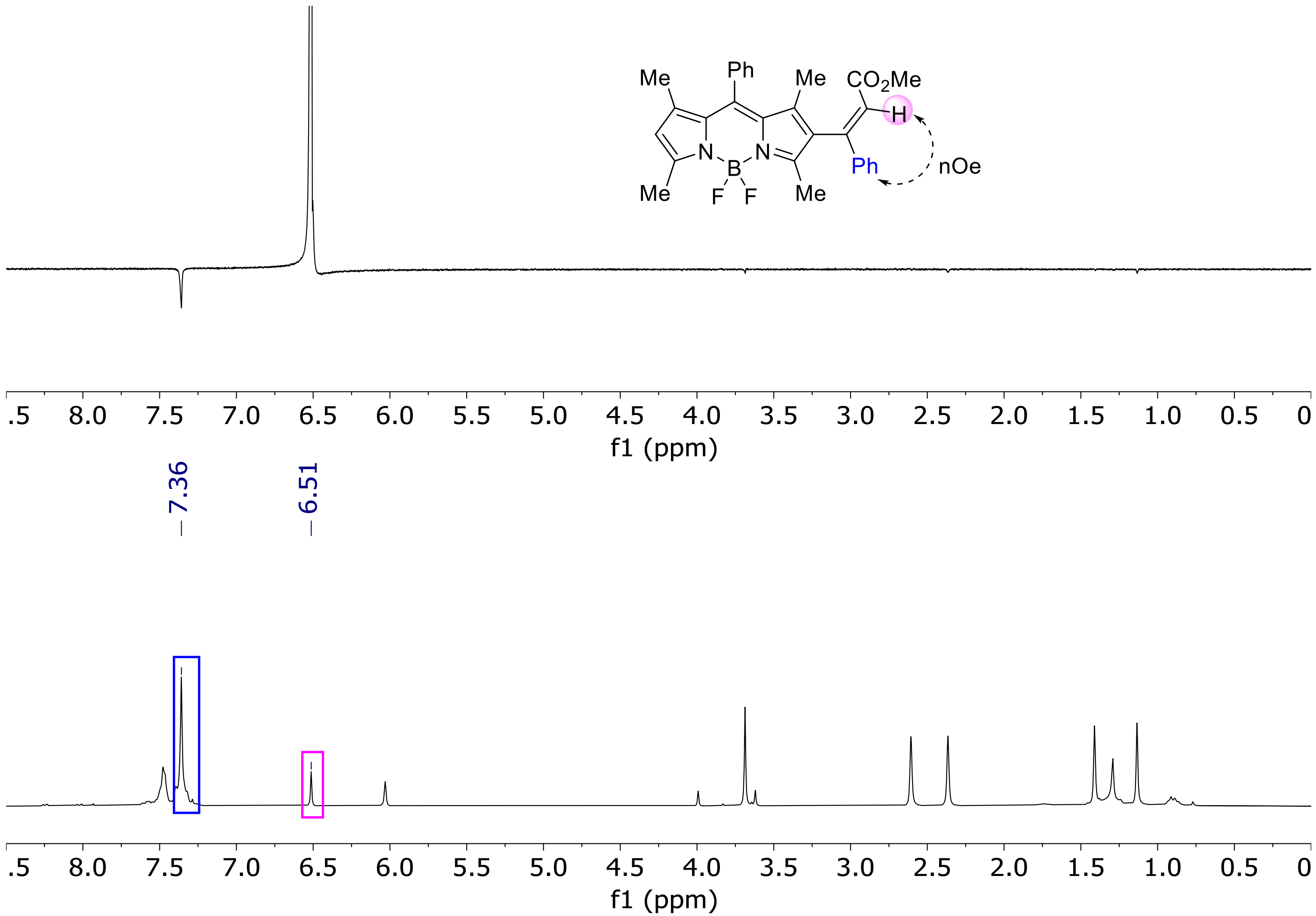

300 MHz ^{1}H-NMR Spectrum of compound **9a** ($CDCl_3$, 300 K)

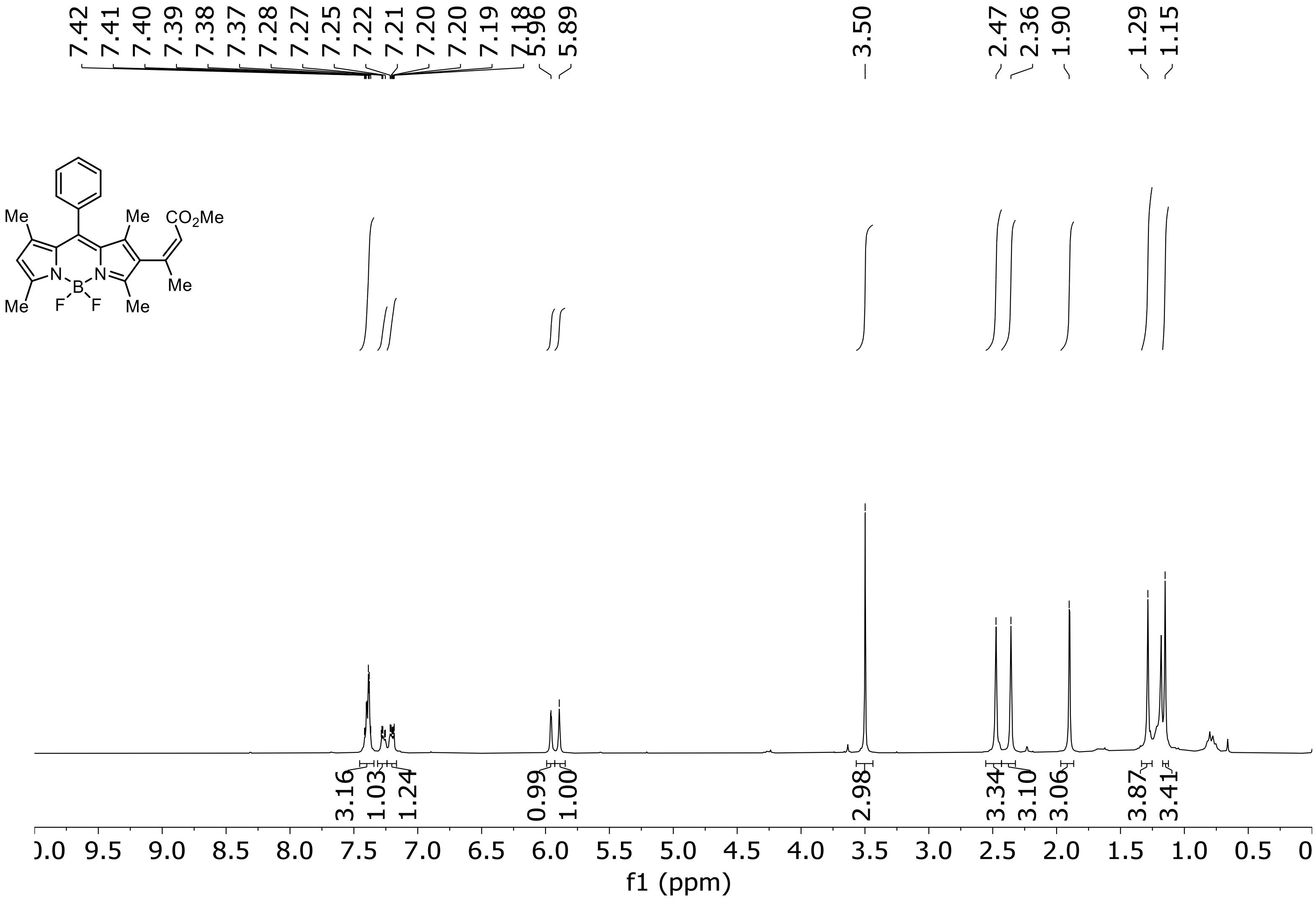

75 MHz $^{13}C\{^{1}H\}$-NMR Spectrum of compound **9a** ($CDCl_3$, 300 K)

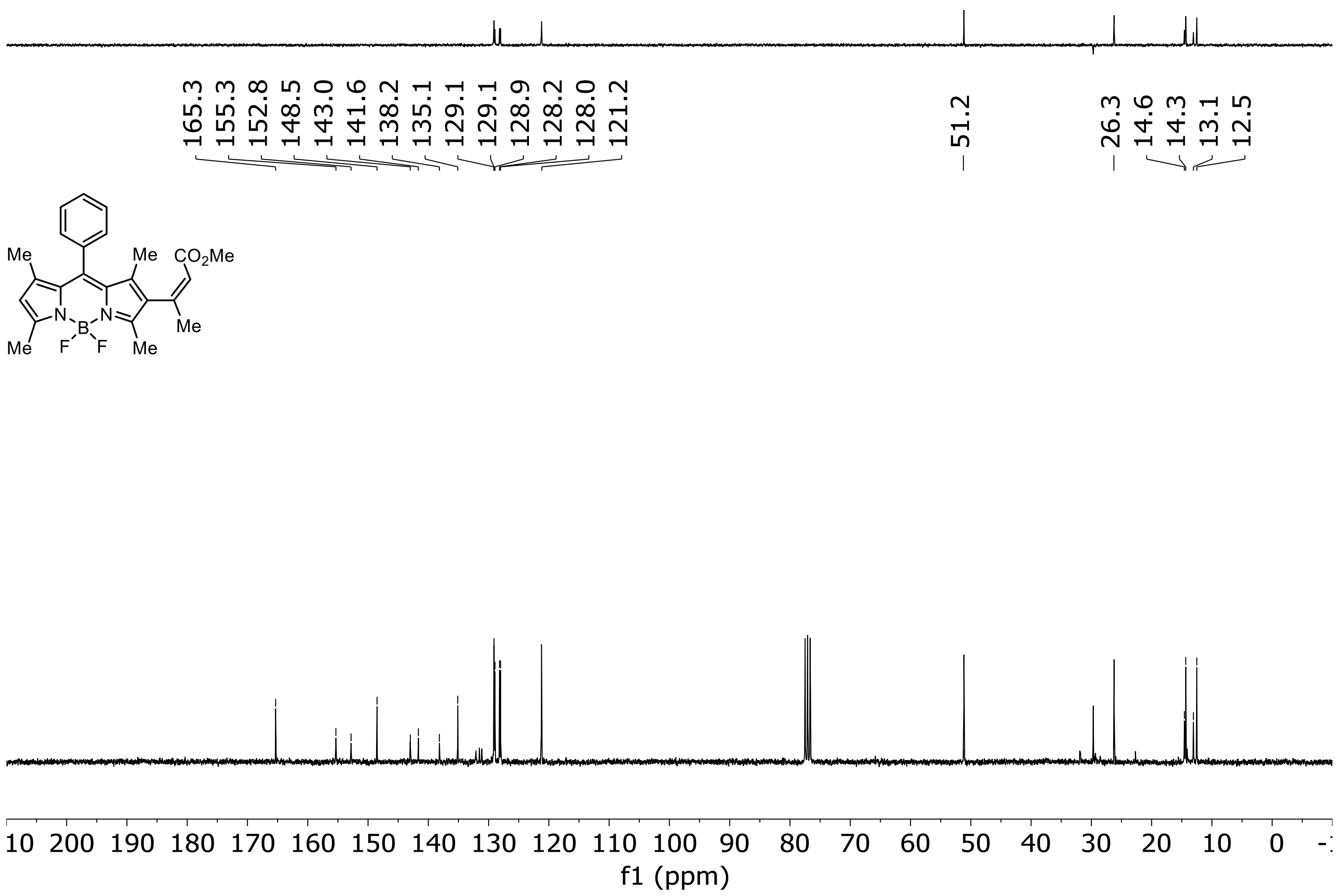

319 MHz ^{19}F-NMR Spectrum of compound **9a** ($CDCl_3$, 300 K)

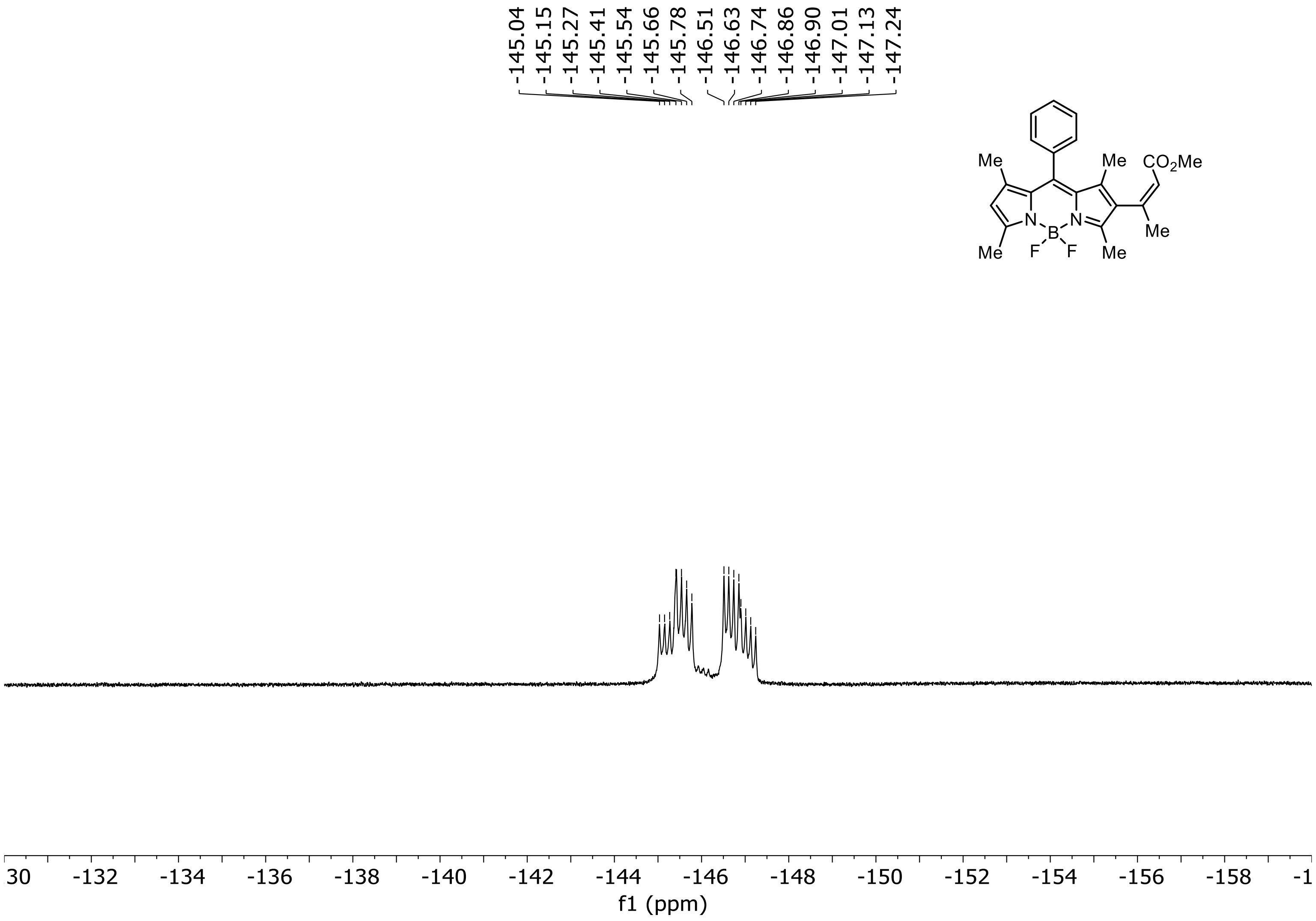

300 MHz ^1H-^1H COSY Spectrum of compound **9a** ($CDCl_3$, 300 K)

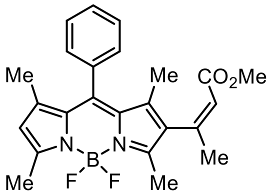

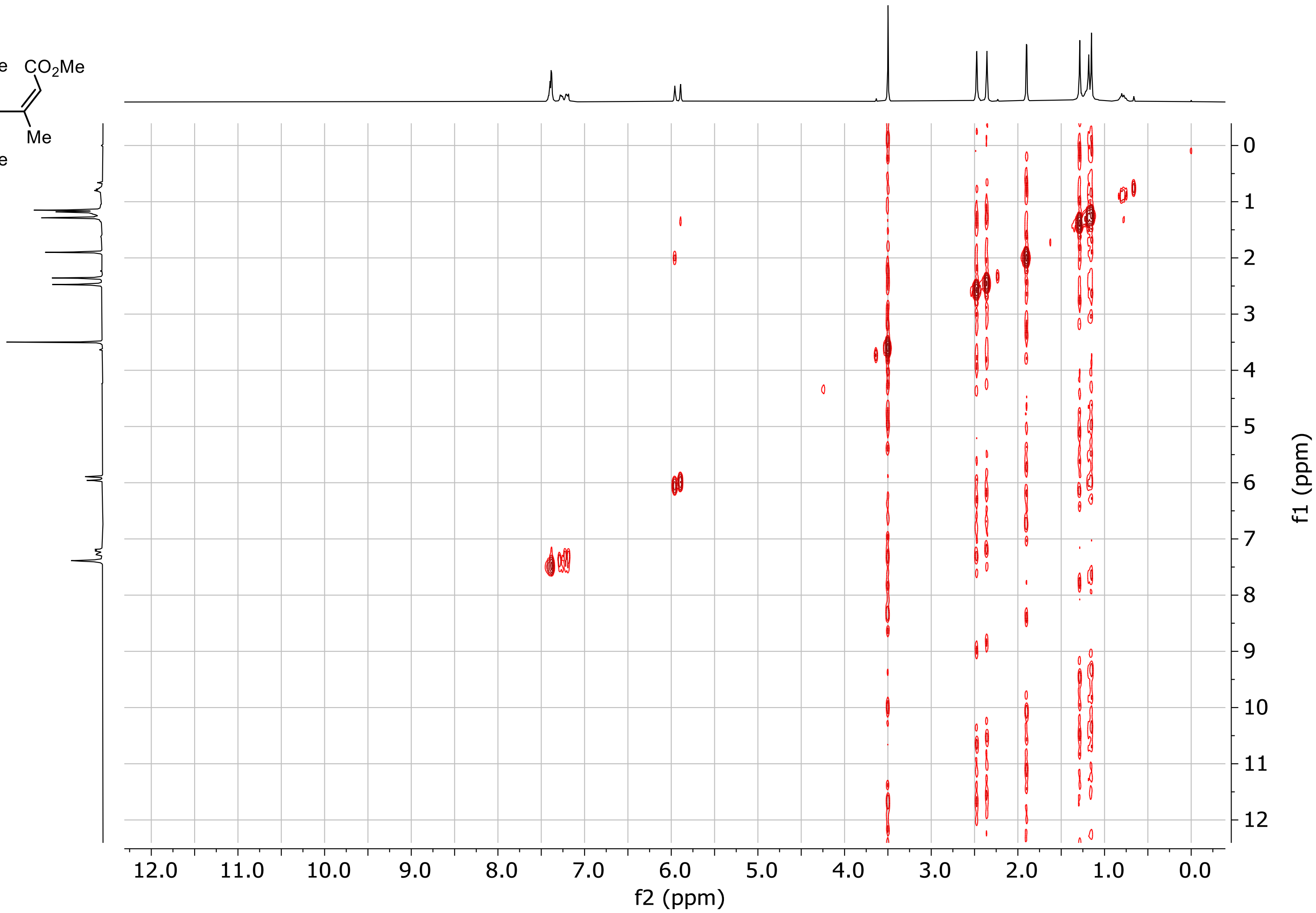

300 MHz HSQC Spectrum of compound **9a** ($CDCl_3$, 300 K)

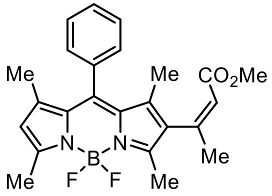

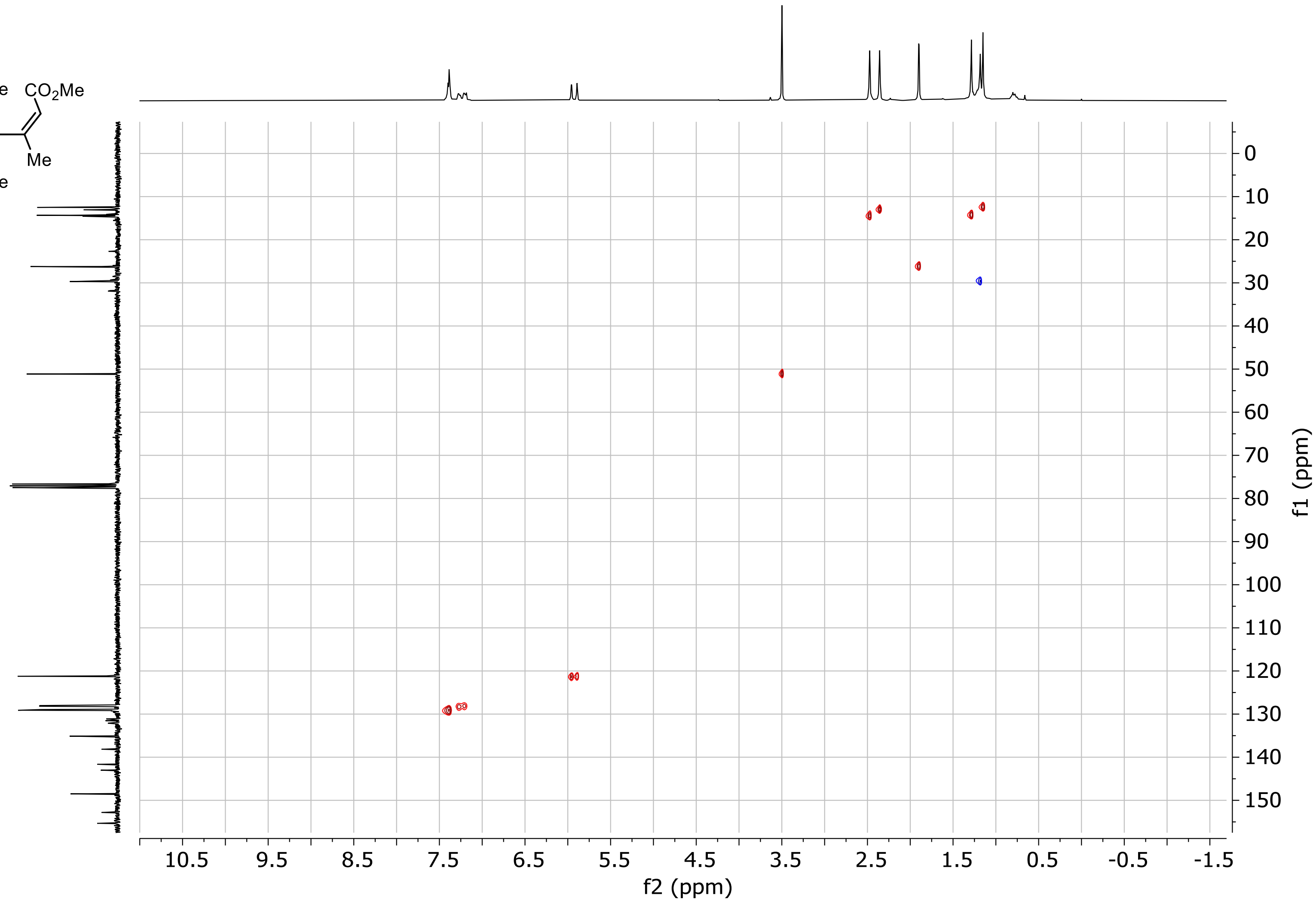

300 MHz HMBC Spectrum of compound **9a** ($CDCl_3$, 300 K)

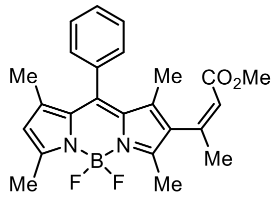

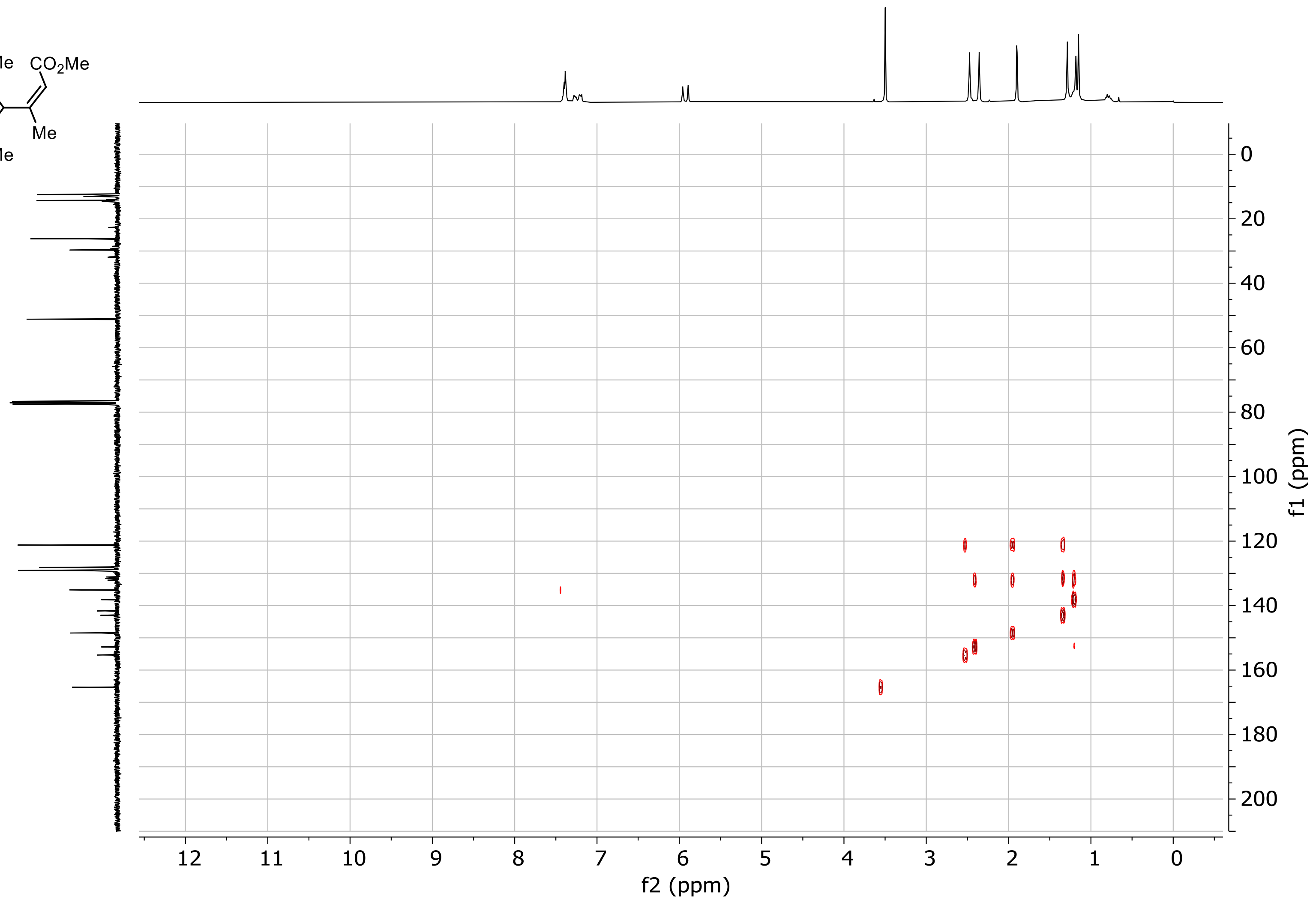

300 MHz NOESY Spectrum of compound **9a** ($CDCl_3$, 300 K)

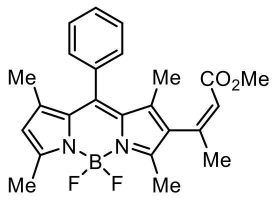

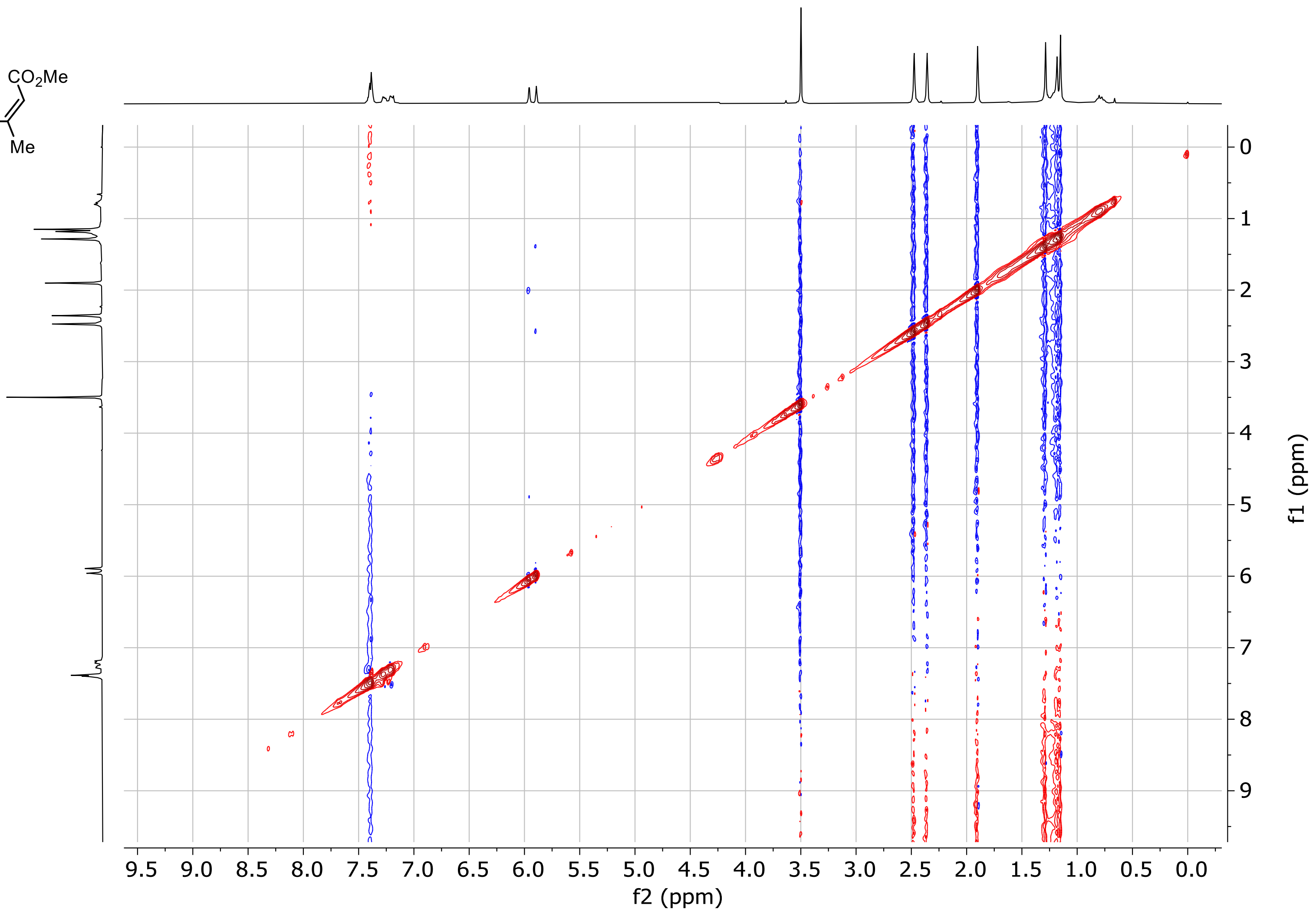

300 MHz [1]D nOe Spectrum of compound **9a** ($CDCl_3$, 300 K) showing the correlation between the hydrogen of the alkenyl group at 6.03 ppm and the methyl hydrogens at 2.00 ppm.

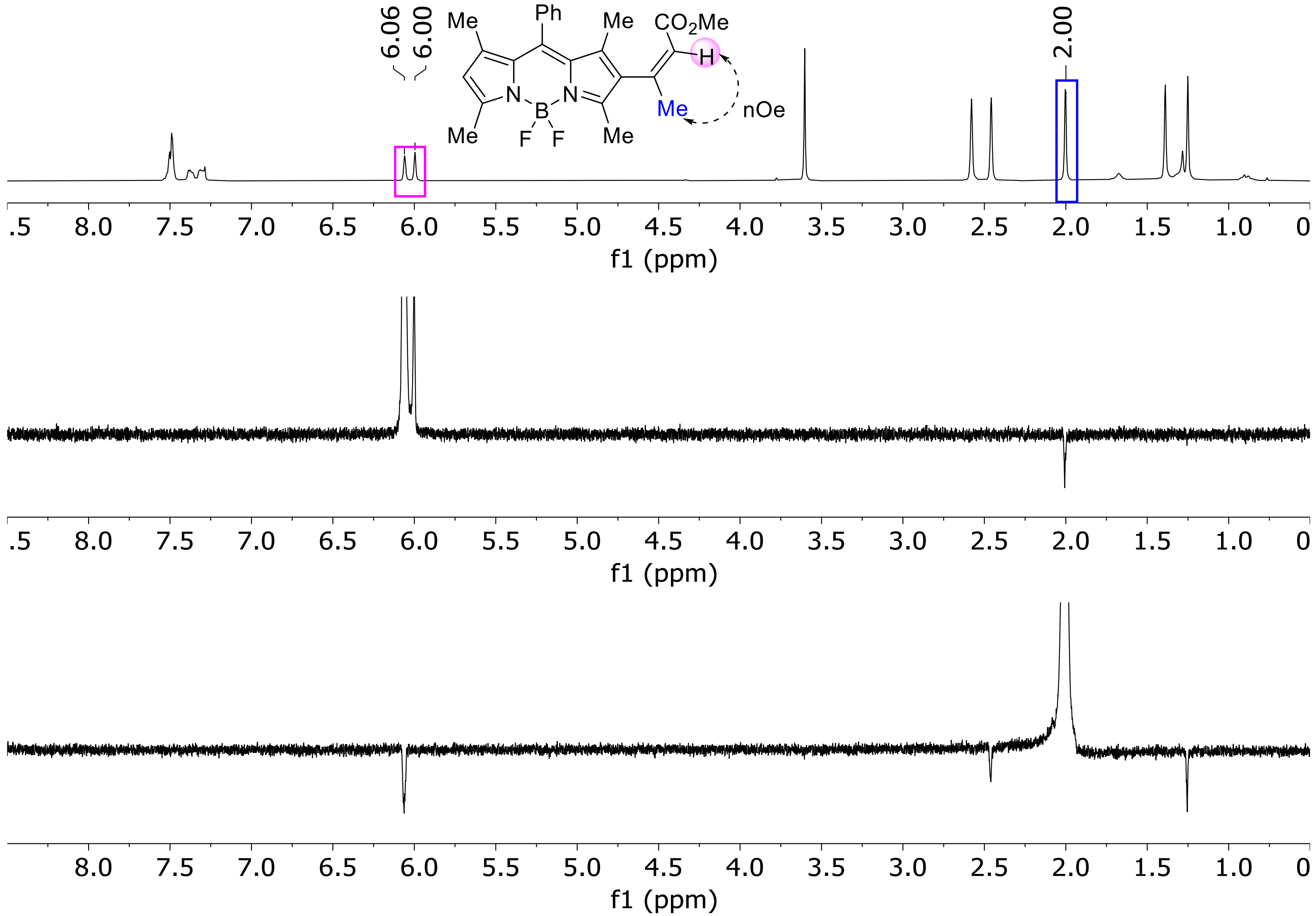

300 MHz ^{1}H-NMR Spectrum of compound **8b** ($CDCl_3$, 300 K)

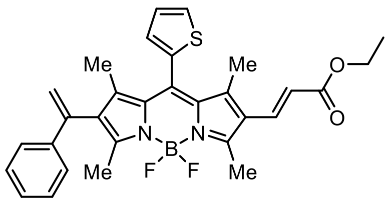

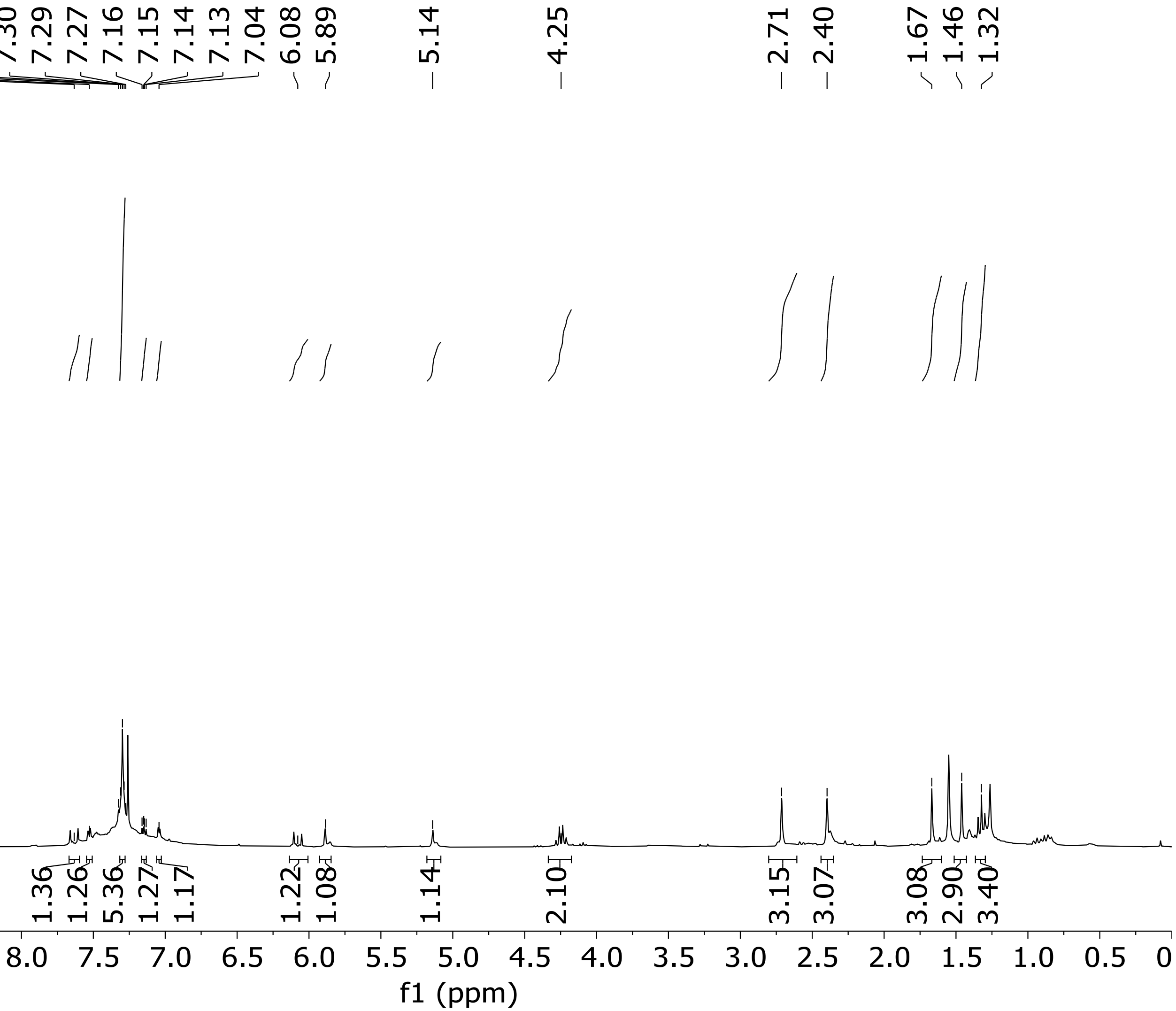

75 MHz $^{13}C\{^1H\}$-NMR Spectrum of compound **8b** ($CDCl_3$, 300 K)

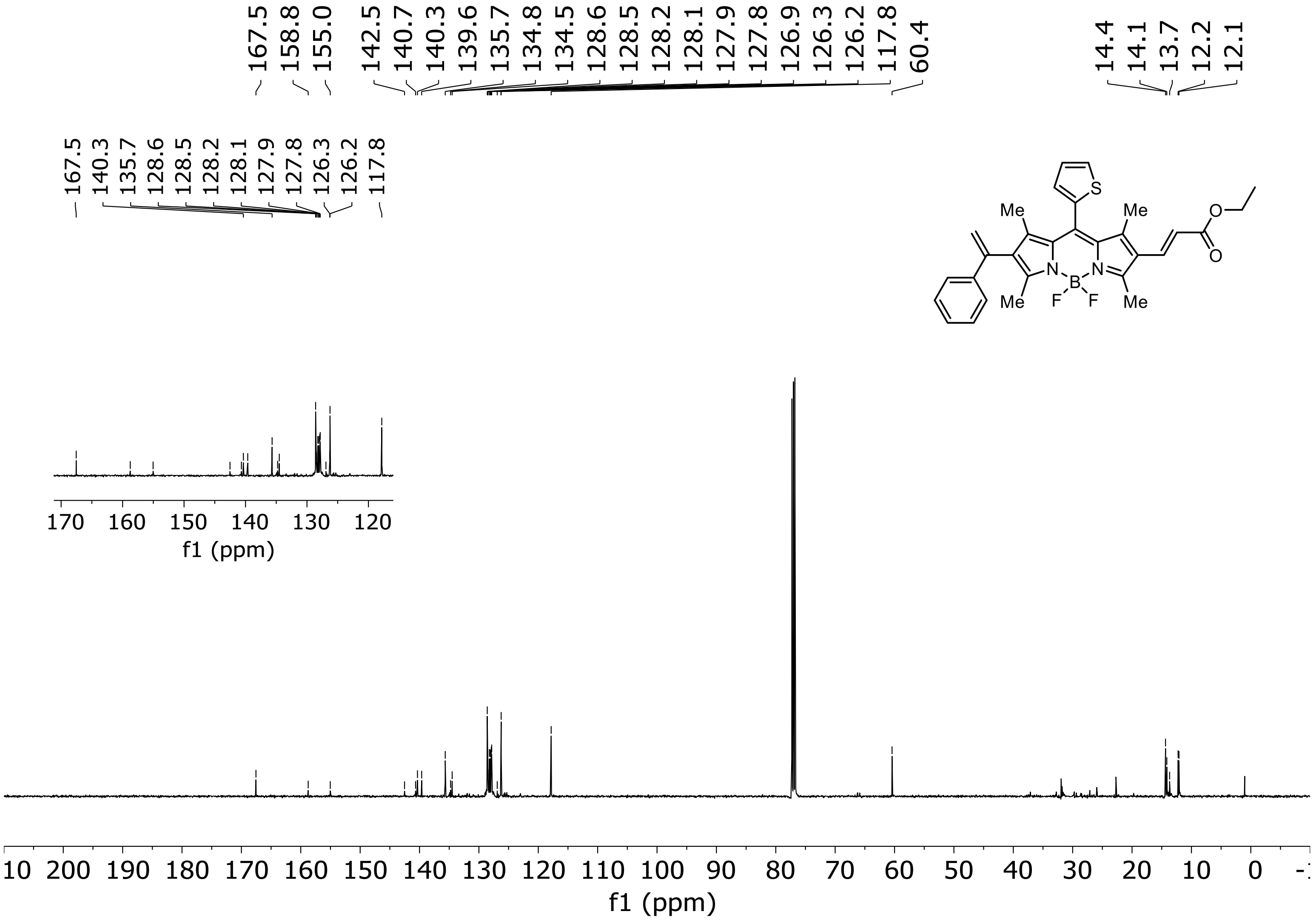

319 MHz ^{19}F-NMR Spectrum of compound **8b** ($CDCl_3$, 300 K)

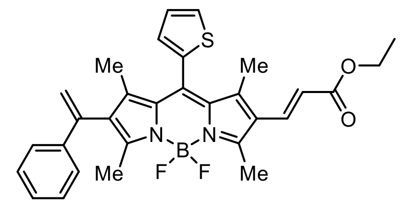

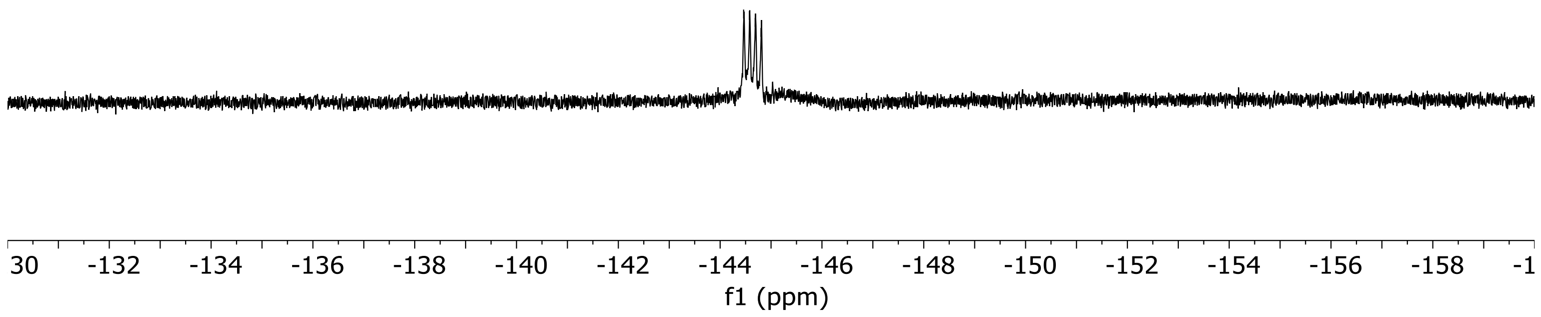

300 MHz ^{1}H-NMR Spectrum of compound **10a** ($CDCl_3$, 300 K)

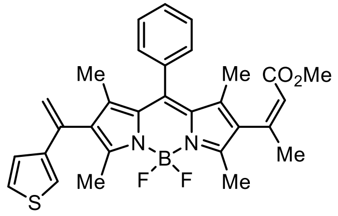

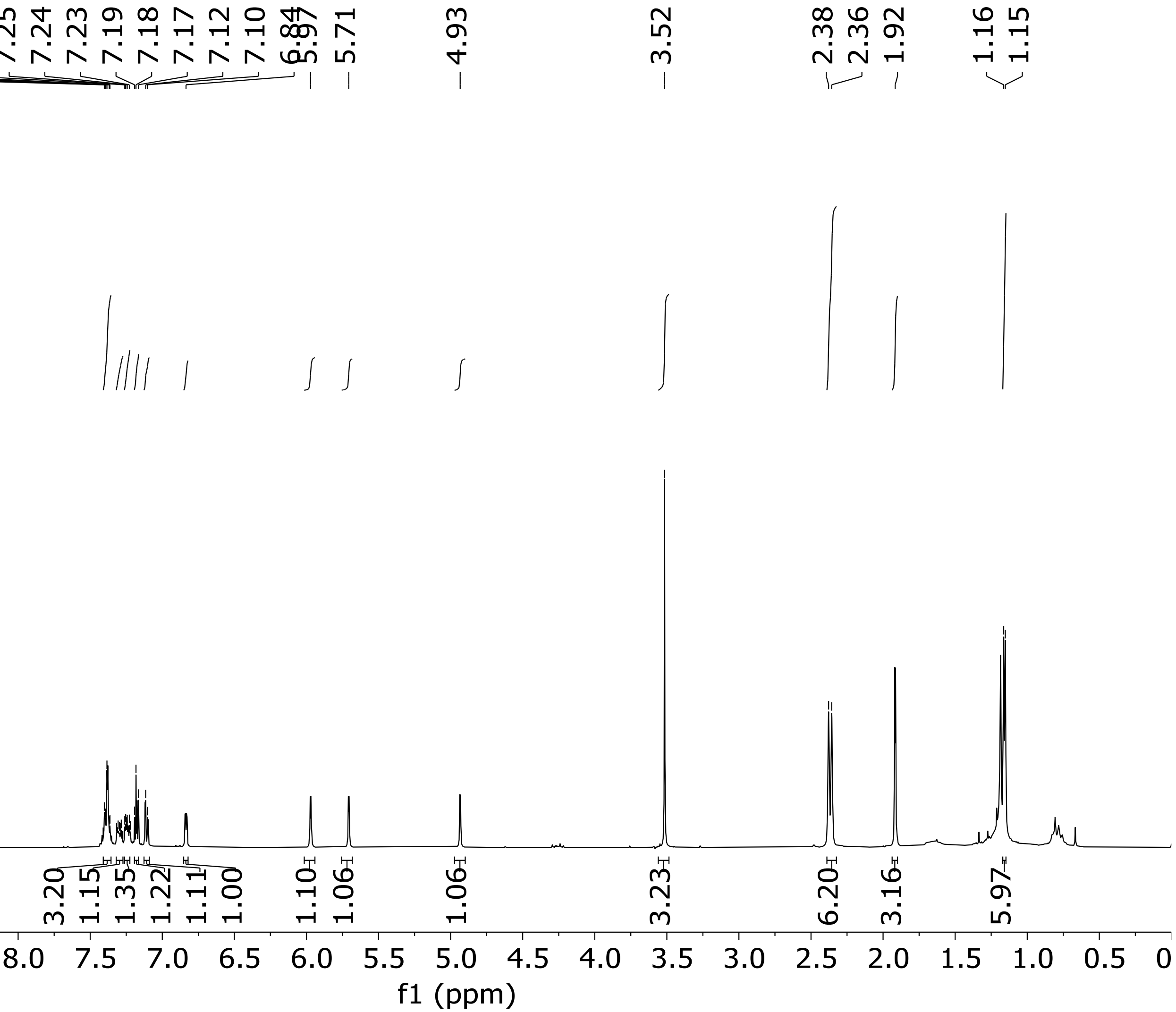

75 MHz $^{13}C\{^{1}H\}$-NMR Spectrum of compound **10a** ($CDCl_3$, 300 K)

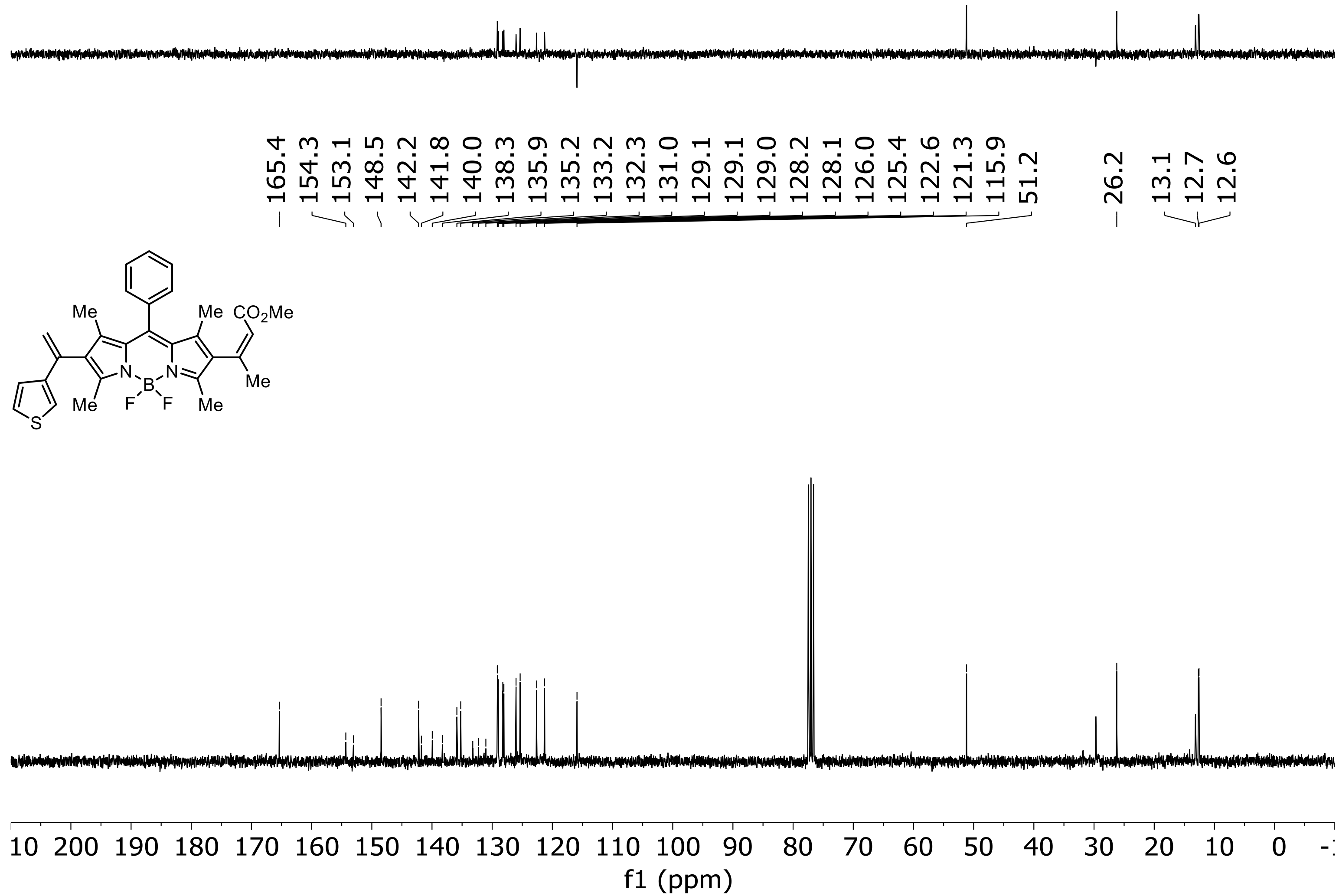

319 MHz ^{19}F-NMR Spectrum of compound **10a** ($CDCl_3$, 300 K)

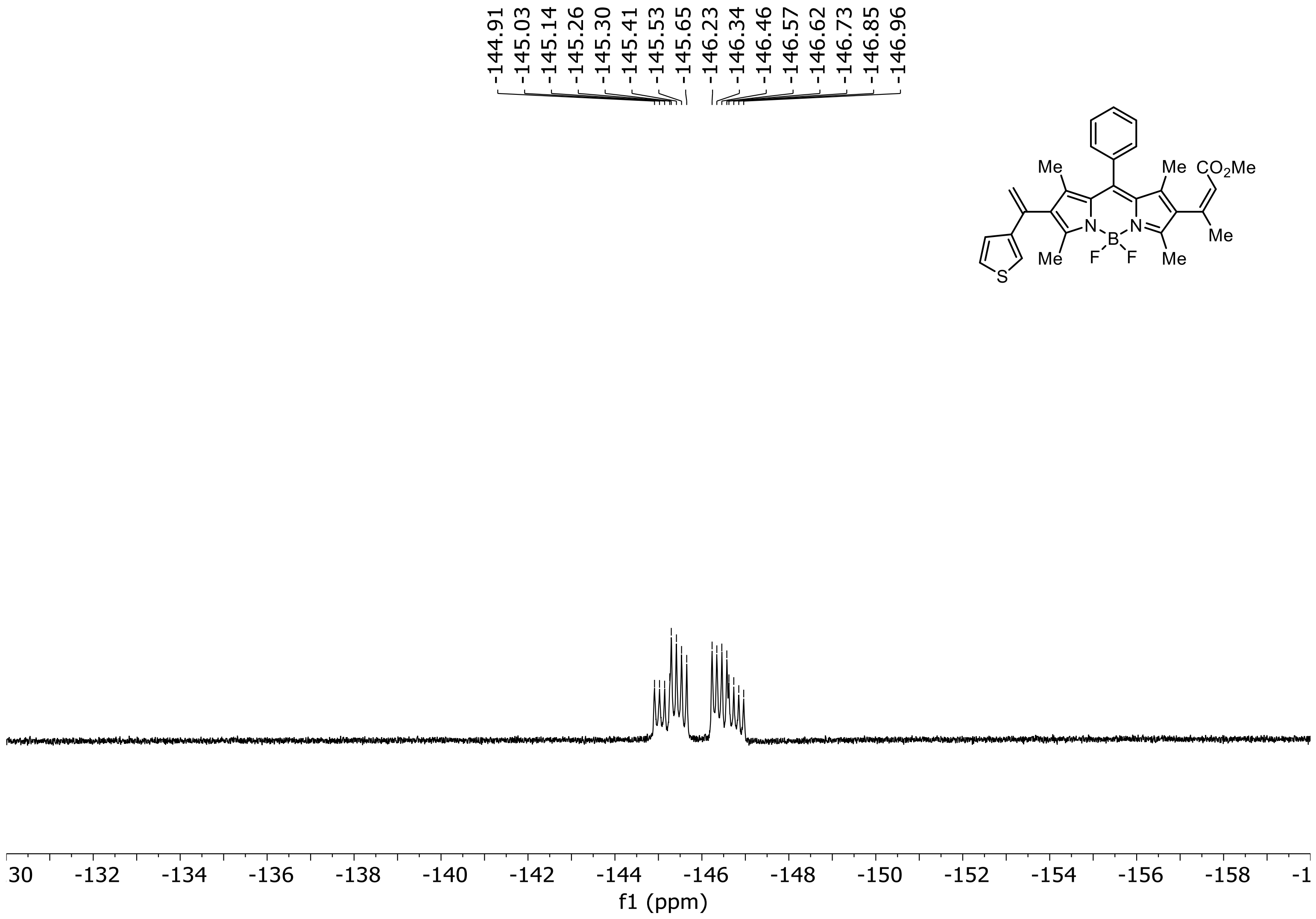

Supplement: Supplementary file 1 — jo3c02951_si_001.pdf [file jo3c02951_si_001.pdf]
